# Diazulenopentalene: Facile Synthesis of Linear Non-Alternant Molecular Carbons through Pt-Mediated Rearrangement

**DOI:** 10.1021/jacs.5c19316

**Published:** 2026-01-30

**Authors:** Zhaohang Lin, Chang Wang, Farshad Shiri, Zhenyang Lin, Junzhi Liu

**Affiliations:** † Department of Chemistry, 25809 The University of Hong Kong, Pokfulam Road, Hong Kong 999077, China; ⊥ Department of Chemistry, The Hong Kong University of Science and Technology, Hong Kong 999077, China; § State Key Laboratory of Synthetic Chemistry, HKU-CAS Joint Laboratory on New Materials and Shanghai-Hong Kong Joint Laboratory on Chemical Synthesis, The University of Hong Kong, Pokfulam Road, Hong Kong 999077, China; # Materials Innovation Institute for Life Sciences and Energy (MILES), HKU-SIRI, Shenzhen 518045, China

## Abstract

Pentalene is a classic 8π antiaromatic non-alternant compound that displays high reactivity. To stabilize pentalene and investigate its properties, benzene rings are typically fused to its core. Advances in metal-catalyzed chemistry have rapidly accelerated the development of pentalene-incorporated molecular carbons. However, fully non-alternant structures based on the pentalene skeleton remain underexplored due to synthetic challenges. Here, we report the synthesis of a novel linear non-alternant hydrocarbon, diazulenopentalene, achieved via a Pt-mediated rearrangement arylation, with the mechanism elucidated by computational studies. Single-crystal analysis, complemented by aromaticity calculations, revealed that diazulenopentalene comprises distinct pentalene and azulene units. The synthetic strategy presented here provides a promising platform for a deeper investigation into this unique linear molecular carbon.

## Graphical abstract

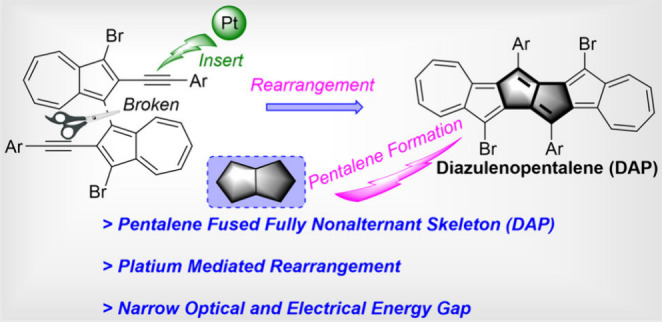

Pentalene, a classic antiaromatic non-alternant hydrocarbon with 8π electrons, exhibits significant dimerization activity even around −196 °C.^1–3^ The first isolated example incorporating bulky substituents dates back to 1973.^1^ Despite the synthetic challenges in obtaining complex pentalene derivatives, chemists have shown great interest in pentalene-embedded linear non-alternant molecular carbons due to their unique electronic structures, intriguing antiaromatic fragments, and tunable physicochemical properties. For instance, dibenzo­[*a,e*]­pentalene (Figure 1a) was first synthesized by Brand in 1912 and proven stable under ambient conditions.^4^ Since then, dibenzo­[*a,e*]­pentalene derivates were extensively studied.^5–8^ Interestingly, transforming the molecular geometry from dibenzo­[*a,e*]­pentalene to dibenzo­[*a,f*]­pentalene induces profound changes in magnetic properties, triggering the emergence of singlet open-shell diradical character (Figure 1a).^9^ More recently, dibenzo­[*a,e*]­pentalene has been employed in the construction of antiaromatic [*n*]­cycloparaphenylenes and covalent organic frameworks, highlighting its potential in advanced material applications.^10,11^ In recent years, larger π-extended pentalene derivates, including dinaphthalene-fused pentalenes, indacene dimers, and diazuleno-*s*-indacene, have been successively synthesized.^7,12–16^ The development of pentalene derivates thereafter can be sharply attributed to the significant advancements in synthetic chemistry and persistent scientific interest in this antiaromatic structural motif.^17–19^

**Figure 1:**
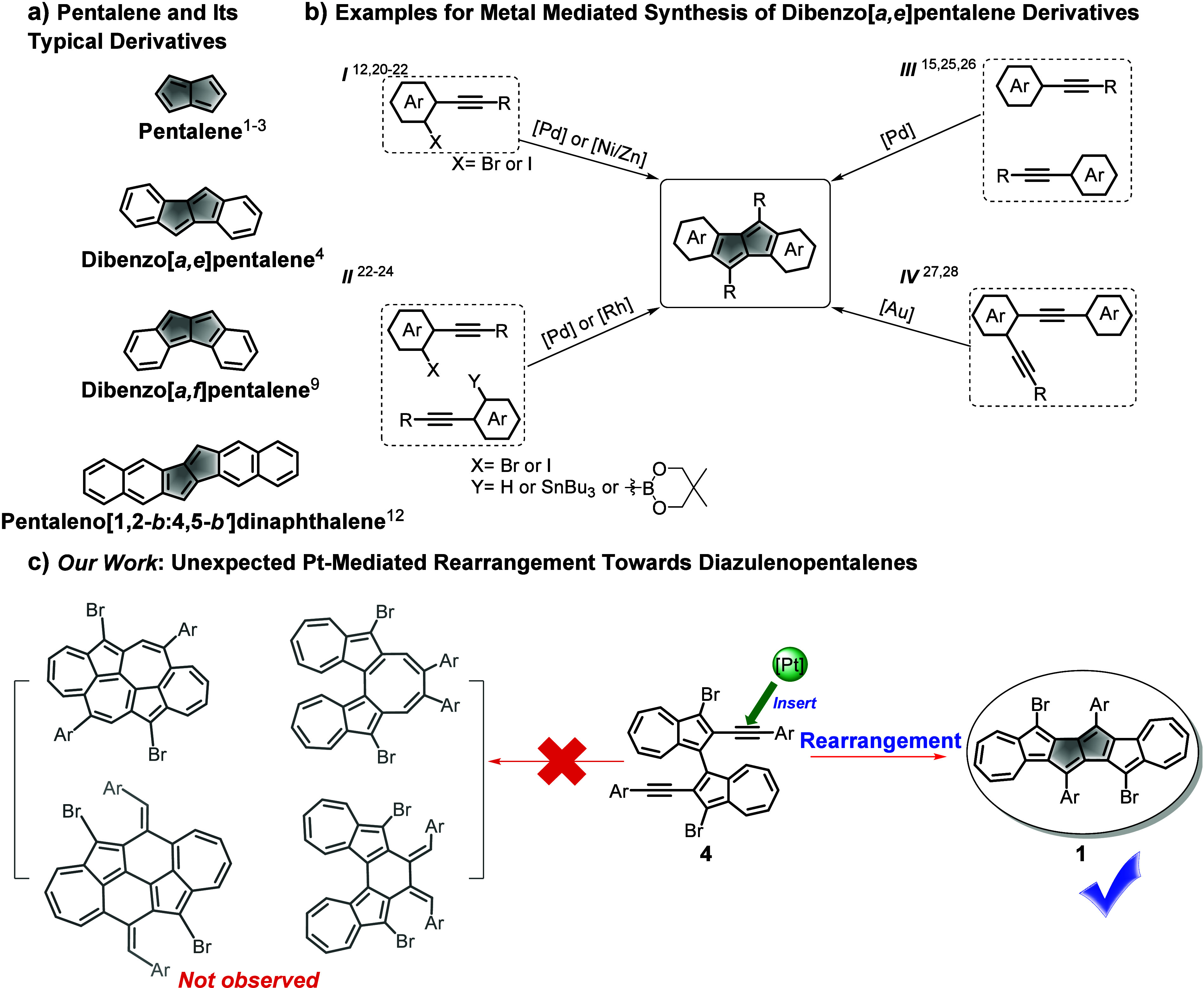
a) Structures of pentalene and some classical pentalene-embedded linear molecular carbons; b) diagram of different types of transition metal mediated coupling reaction to synthesize dibenzo­[*a,e*]­pentalene derivatives; c) facile synthesis of diazulenopentalenes through Pt-mediated rearrangement (this work).

Reviewing the synthesis of benzo-annulated pentalenes reveals a significant gap; the dibenzo­[*a,e*]­pentalene skeleton, first discovered in 1912, lacked a general synthetic route until Youngs reported one in 1999 using standard Sonogashira coupling conditions (Figure 1b, path I).^4,16,20^ In 2009, Kubo reported a similar homocoupling reaction (Figure 1b, path I), yielding dibenzopentalenes with moderate yield.^21^ In the same year, Tilley concurrently reported two palladium-catalyzed (Pd) routes, a homocoupling of aryl and alkenyl halides (Figure 1b, path I) and a cross-coupling of aryl bromides and aryl stannanes (Figure 1b, path II).^22^ In 2013, Jin reported a similar strategy, achieving moderate to high yields.^23^ Beyond Pd, rhodium (Rh) catalysts were later shown to enable cross-coupling reaction (Figure 1b, path II).^24^ In 2013, Itami pioneered a non-halide approach, constructing the dibenzopentalene skeleton through C–H activation (Figure 1b, path III).^25,26^ Alternatively, gold (Au) catalysis furnished the product in excellent yield via either intramolecular enediyne coupling (Figure 1b, path IV) or a pathway involving alkyne annulation with aryl migration.^27–31^ Despite these significant advances in synthetic chemistry, the precise bottom-up synthesis of linear fully non-alternant molecular carbons incorporating multiple nonbenzenoid units (e.g., azulene-pentalene hybrids) remains elusive.

In this work, we designed key precursor **4** featuring two azulene-alkyne units. To investigate whether pentalene formation can be achieved in nonbenzenoid systems, we employed platinum (Pt) catalysis, which has been extensively utilized for the cyclization of arylacetylene into five- and six-membered rings,^32–37^ on this diazulene-alkyne scaffold (Figure 1c). Intriguingly, **4** exhibited no five-/seven- or five-/six-/seven-membered ring nor underwent self-alkyne-coupling to produce five-/seven-/eight- or five-/six-/seven-membered systems (Figure 1c). Instead, **4** generated a novel linear non-alternant molecular carbon, pentaleno­[2,1-*a*:5,4-*a’*]­diazulene (DAP, **1**), containing exclusively five- and seven-membered rings through Pt-mediated rearrangement. To our knowledge, this represents the first reported Pt-mediated rearrangement forming azulene-fused pentalene derivates, overcoming the longstanding challenge of constructing fully non-alternant linear molecular carbons.

Scheme 1 outlines the synthetic route to DAP skeleton **1**, starting from 2-iodoazulene (**azu-I**). Compound **2** was obtained through intermolecular oxidative coupling of **azu-I** under −78 °C with a yield of 70%. Subsequent bromination of **2** with NBS yielded **3** in 78% yield. The key precursor **4** was formed after the selective Sonogashira coupling of **3** with arynes with a yield of 20–32%. Crucially, Pt-mediated arylation of **4** underwent rearrangement to afford the fully non-alternant molecular carbon **1** (diazulenopentalene) featuring fused azulene-pentalene units in 8–15% yields. To our knowledge, this underexplored Pt-mediated rearrangement reaction has rarely been investigated, offering a promising platform for probing the structure–property relationships in diazulenopentalene derivates. More notably, the rearrangement of precursor **4-Ib** also proceeded, affording product **1-Ib** in 15% yield, with a structure consistent with compounds reported by Chi and co-workers.^38^

**Scheme 1:**
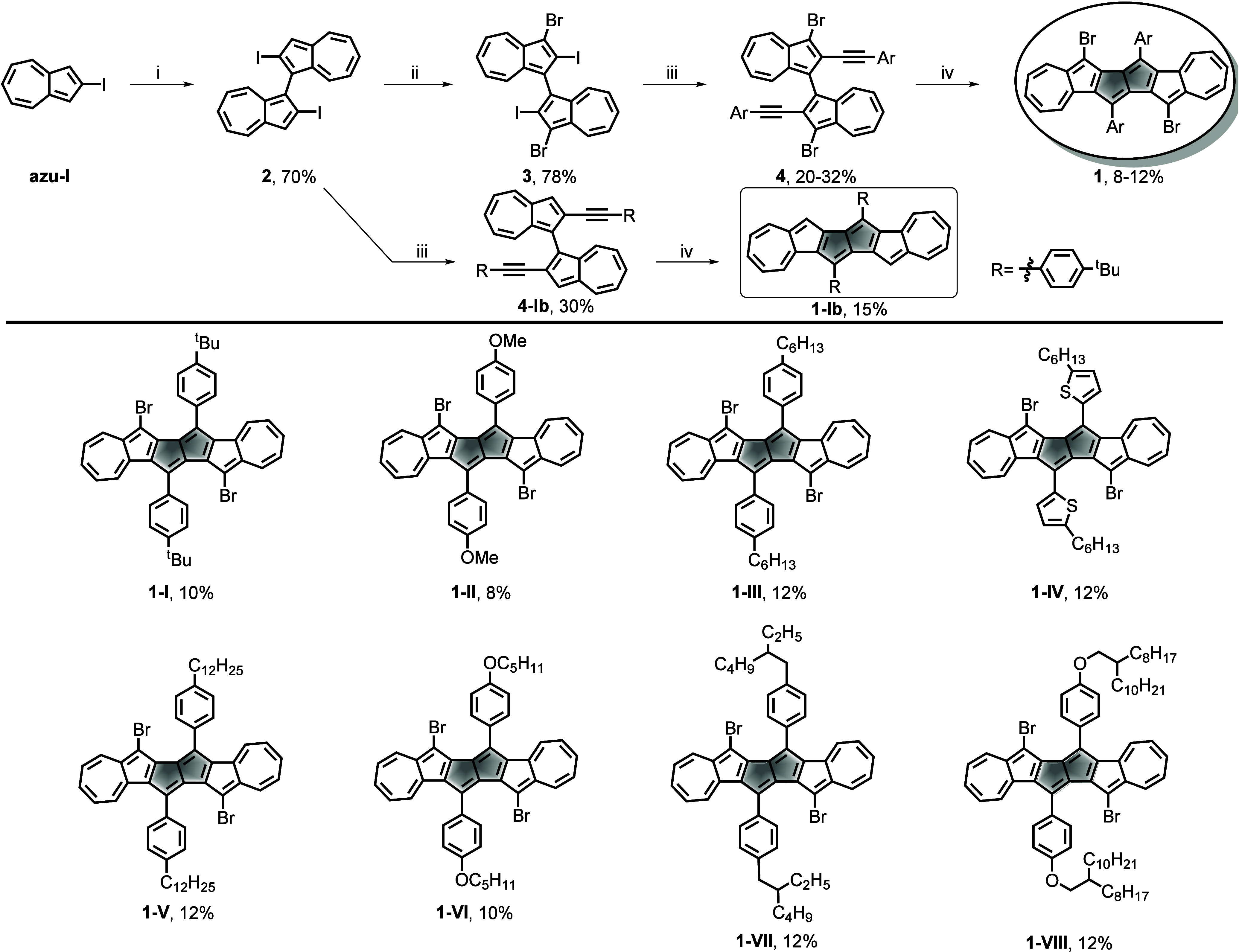
Synthetic Route to DAP Derivatives^a^

a (i) FeCl_3_, DCM, −78 °C, 30 min, 70%; (ii) NBS, DCM, 0 °C, 30 min, 78%; (iii) Pd­(PPh_3_)_2_Cl_2_, CuI, THF:Et_3_N (5:1), rt, 1 h, 20–32%; (iv) PtCl_2_, *o*-xylene, 120 °C, 4 h, 8–15%. Abbreviations: DCM, dichloromethane; NBS, *N*-bromosuccinimide; THF, tetrahydrofuran; Pd­(PPh_3_)_2_Cl_2_, bis­(triphenylphosphine)­palladium­(II) chloride; Et_3_N, triethylamine; PtCl_2_, platinum­(II) chloride.

To further investigate the mechanism of this interesting cyclization, we carried out density functional theory (DFT) calculations at the SMD/M06/def2-TZVP//SMD/M06/def2-SVP level of theory; we used **4-II** as the substrate to characterize the elementary steps of the transformation and thereafter identify the reaction pathway leading to the formation of diazulenopentalene **1-II**. Figure 2 and Figures S3 and S4 displayed the energy profiles calculated for the plausible rearrangement pathway of this reaction, in which initially PtCl_2_ coordinates with one side alkyne moiety, thereafter, undergoing *cis–trans* isomerization to form **IM2** with an exergonicity of 27.7 kcal/mol. It should be noted that **IM2** was essential to enable subsequent intramolecular cyclization. Subsequent nucleophilic attack by the azulene unit on the Pt-activated alkyne with a barrier of 4.5 kcal/mol yields the σ-vinylplatinum­(II) intermediate **IM3**. Two possible pathways were available for the reaction to proceed. Path a proceeded via a similar nucleophilic attack by another azulene unit on the nonactivated alkyne via **TS3** with a high barrier of 39.2 kcal/mol, which was thus inaccessible. On the other hand, path b proceeded via the second coordination of PtCl_2_ with the other alkyne, forming intermediate **IM5** again with an exergonicity of 27.7 kcal/mol. Similar to the formation of **IM3** from **IM2**, **IM6** was generated with a barrier of 4.8 kcal/mol from **IM5** via the nucleophilic attack. Skeletal rearrangement of the two central pentagon moieties in **IM6** into the octagon core of **IM7** via **TS6**, accompanied by cleavage of the central C–C bond, constitutes the rate-determining step (Δ*G* ^‡^ = 28.7 kcal/mol), consistent with the experimentally required temperature. Formation of the pentalene core from **IM7** occurs via **TS7** in a barrierless step, followed by expulsion of two PtCl_2_ molecules to achieve target compound **1-II**. The overall process was highly exergonic, providing strong thermodynamic driving force for the cascade cyclization.

**Figure 2:**
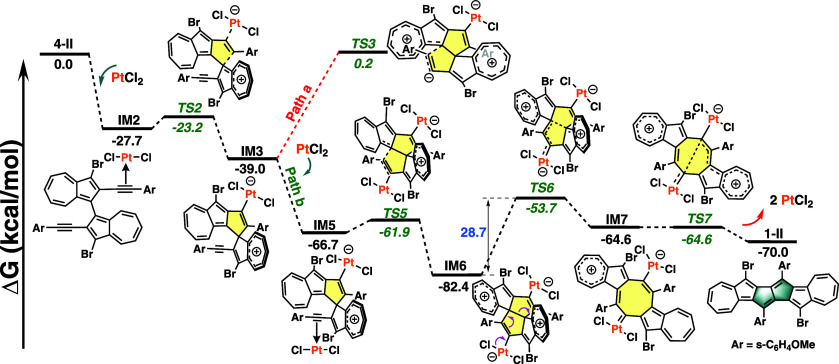
Gibbs free energy profiles for a plausible Pt-mediated rearrangement mechanism. Calculated at the SMD/M06/def2-TZVP//SMD/M06/def2-SVP level of theory.

A single crystal of **1-I** suitable for X-ray diffraction was obtained by slowly evaporating dichloromethane/methanol mixtures. As shown in Figure 3a, the DAP skeleton adopts a planar geometry with a length of 15.187 Å. The central pentalene unit (ring A/A′) displayed obvious bond-length alternation (1.377–1.476 Å), aligning with the intrinsic electronic structure of pristine pentalene. The peripheral azulene units (rings B+C/B′+C′) exhibit bond-length patterns and electron density distributions characteristic of pure azulene, preserving the 10π electronic configuration intrinsic to azulene systems. The molecular stacking behaviors were further investigated, as shown in Figure 3b and Figure S1. **1-I** exhibits a sheet-like packing style (or β packing type), dominated by a 3.358 Å π–π interaction between two peripheral heptagon units (Figure 3b).

**Figure 3:**
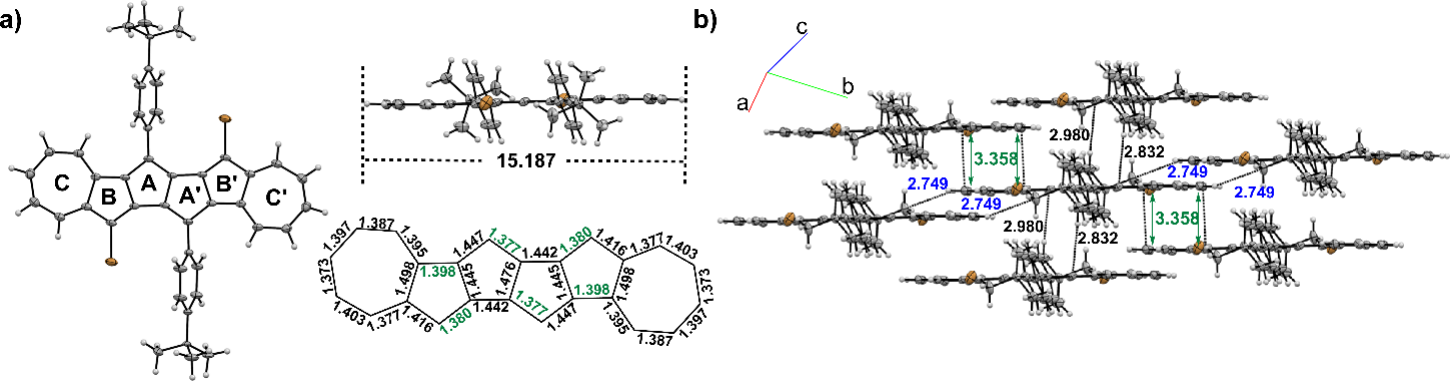
Single-crystal X-ray structures of compound **1-I** with ellipsoids at the 30% probability level. a) Top and side views of **1-I** and bond length of the DAP skeleton. b) The molecular packing for one molecule of **1-I** in a side view. (length unit: Å)

The optical properties of DAPs were investigated by UV–vis absorption spectroscopy. As shown in Figure 4a, phenyl-substituted derivatives **1-I–III** displayed nearly identical absorption onsets with the longest-wavelength absorption peak at 505 nm, which could be assigned to HOMO → LUMO along with HOMO–1 → LUMO+1 transitions (Table S4 and Figure S69). Thiophene-substituted compound **1-IV** showed similar absorption behaviors with the longest-wavelength absorption peak at 510 nm, which slightly bathochromically shifts compared to phenyl-substituted derivatives. When the alkyl chain increased, phenyl-substituted derivatives **1-V–VIII** also displayed almost identical absorption behaviors, with a longest-wavelength peak at around 505 nm as depicted in Figure 4a. **1-Ib** exhibited a comparable absorption peak at around 500 nm compared with **1-I**, except for the additional peak at 365 nm. All the diazulenopentalene derivates exhibited extended absorption tails reaching into the near-infrared (NIR) region, characteristic of azulene-containing systems.

**Figure 4:**
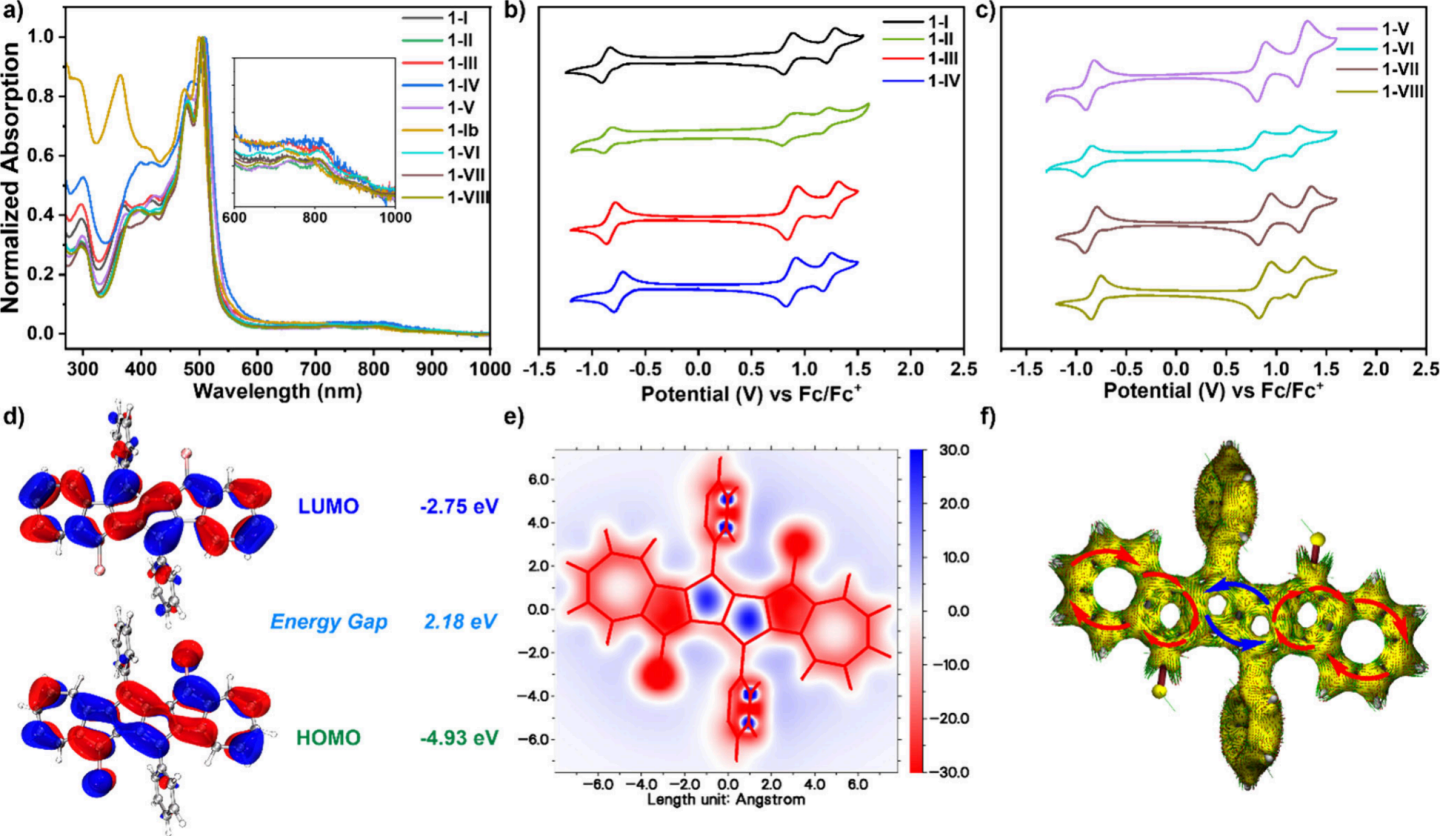
a) Normalized UV–vis absorption spectra of diazulenopentalenes **1-I** to **1-VIII** in DCM. CVs of diazulenopentalenes **1-I** to **1-IV** (b) and **1-V** to **1-VIII** (c) in DCM (0.1 M *n*-Bu_4_NPF_6_/DCM, scan rate: 100 mV/s for CV, room temperature). d) Frontier molecular orbital profiles and energy gaps of **1-I** (isovalue = 0.02) at the B3LYP/6-311G­(d,p) level of theory; the alkyl chain was removed to simplify the calculation. e) 2D NICS_*zz*_ of **1-I** at the GIAO-B3LYP/6-311+G­(2d,p) level of theory. f) ACID plot of **1-I** at the CSGT-B3LYP/6-311G* level of theory. Diamagnetic (clockwise) and paramagnetic (counterclockwise) ring currents under a magnetic field parallel to the *z*-axis are highlighted by red and blue arrows, respectively.

The electrochemical properties of diazulenopentalenes were investigated by cyclic voltammograms (CV). CV measurements were recorded in dichloromethane (Figure 4b and 4c). All of the diazulenopentalene derivatives displayed two reversible oxidation waves and one reversible reduction wave, consistent with the diazulenopentalene core structure. Specifically, **1-I** exhibited reversible oxidation waves at 0.77 and 1.15 V, as well as one reversible oxidation wave at −0.68 V. According to the oxidation and reduction waves of derivatives, the electrical energy levels for the highest occupied molecular orbital/the lowest unoccupied molecular orbitals (HOMO/LUMO) were summarized in Table S3.

Time-dependent density functional theory (TD-DFT) calculations were carried out to investigate the electronic characteristics of the DAP skeleton. We selected **1-I** (benzene substituted) and **1-IV** (thiophene substituted) as model compounds and removed their alkyl chains to simplify the calculations. To compare the effect of non-alternant and alternant, pentaleno­[1,2-*b*:4,5-*b′*]­dinaphthalene derivative **5** was employed for comparison with derivatives **1-I** and **1-IV**. Geometry optimization was performed using the Gaussian16 package, and further graphic processing was finished via the Multiwfn and VMD visualization program.^39,40^ HOMOs of **1-I** and **1-IV** were mainly delocalized on double bonds at the central pentalene unit (ring A+A′) as well as peripheral azulene units (ring B+C/B′+C′), as shown in Figure 4d and Figure S68. LUMOs of **1-I** and **1-IV** exhibit a key distinction from the HOMO, with pronounced localization on the single bonds within the central pentalene unit (rings A/A′), contrasting with HOMO delocalization across double bonds. The energy gap of the DAP skeleton was thereafter calculated to be around 2.18 eV for **1-I** and 2.12 eV for **1-IV**, both smaller than that of **5** (2.83 eV, Figure S68).

The local aromaticity of **1** and **5** was investigated based on the nucleus-independent chemical shift (NICS) calculated at the GIAO-B3LYP/6-311+G­(2d,p) level (Figure 4e and Figures S67 and S68). Specifically, the 2D NICS map of **1-I** and **1-IV** exhibited that ring B/B′ displayed a strong aromatic property, whereas ring C/C′ (seven-membered ring) showed weak aromatic character. However, the central pentalene unit (ring A+A′) was calculated to be pronouncedly antiaromatic, which was identical with pentalene (Figure 4e). The local aromatic characters of **1** and **5** were further corroborated by anisotropy of the induced current density (ACID) plots based on the CSGT-B3LYP/6-311G* level, revealing the matched ring currents with the results from NICS calculations (Figure 4f and Figure S68). The above results revealed the localized aromaticity on azulene moieties along with the localized antiaromaticity on the central pentalene unit in the whole DAP backbone. Collectively, these results demonstrate that the electronic structure of the DAP skeleton emerges directly from the fusion of antiaromatic pentalene and aromatic azulene units, which is divergent from other reported skeletons containing odd-membered rings as shown in Figure S71.^14,15,41–46^

In conclusion, we report the synthesis of a novel linear non-alternant molecular carbon, diazulenopentalene (**1**), featuring exclusively five- and seven-membered rings, achieved through a Pt-mediated rearrangement with up to 9 examples. A plausible mechanistic pathway for this rearrangement was proposed and supported through a DFT calculation. The chemical structure of **1** was unambiguously confirmed by single-crystal X-ray diffraction. Critical bond-length alternation and pronounced antiaromaticity in the central pentalene unit (NICS = 27.05) mirror those of pristine pentalene, demonstrating that **1** electronically integrates distinct antiaromatic pentalene and aromatic azulene domains. This synthetic strategy establishes a versatile platform for developing extended linear molecular carbon with tailored non-benzenoid topologies.

## Supplementary Material



## Notes

Experimental section, NMR spectra, high-resolution mass spectrometry (HRMS) spectroscopic data, DFT calculations, and additional supporting figures/tables (PDF)
