# Supplementary material for: Diazulenopentalene: Facile Synthesis of Linear Non-Alternant Molecular Carbons through Pt-Mediated Rearrangement
Source: J Am Chem Soc. 2026 Jan 30;148(5):4844–50. doi: 10.1021/jacs.5c19316 (PMC12903861; doi:10.1021/jacs.5c19316)
Supplement: Supplementary file 1 [file ja5c19316_si_001.pdf]

Supporting Information

Diazulenopentalene: Facile Synthesis of Linear Non-Alternant Molecular Carbons Through Pt-Mediated Rearrangement

Zhaohang Lin,[†,‡] Chang Wang,[†,‡] Farshad Shiri,[⊥] Zhenyang Lin[⊥] and Junzhi Liu[†,§,#,*]

[†]Department of Chemistry, The University of Hong Kong, Pokfulam Road, Hong Kong 999077, China.

[⊥]Department of Chemistry, The Hong Kong University of Science and Technology, Hong Kong 999077, China

[§]State Key Laboratory of Synthetic Chemistry, HKU-CAS Joint Laboratory on New Materials and Shanghai-Hong Kong Joint Laboratory on Chemical Synthesis, The University of Hong Kong, Pokfulam Road, Hong Kong 999077, China.

[#]Materials Innovation Institute for Life Sciences and Energy (MILES), HKU-SIRI, Shenzhen 518045, China.

[‡]These authors contributed equally: Zhaohang Lin & Chang Wang

E-mail: juliu@hku.hk

Table of Contents

1. Experimental section 3
2. X-ray crystallographic analysis 12
3. Mechanism 16
4. Additional CV 18
5. NMR spectra and Mass spectra 19
6. Theoretical calculations 50
7. Others 56
8. References 57

1. Experimental Section

All reagents and starting materials were purchased from commercial suppliers and used without further purification unless otherwise noted. All reaction conditions dealing with air- and/or moisture-sensitive compounds were carried out under nitrogen atmosphere. Preparative thin layer chromatography (P02015, Analtech Brand Silica Gel GF TLC Plates 2000 μm 20x20 cm) was bought form Miles Scientific company and used as received. The ^{1}H Nuclear Magnetic Resonance (NMR) and ^{13}C NMR were recorded on Advance DRX Bruker 400MHz and/or 600MHz FTNMR spectrometer. ^{1}H and ^{13}C NMR chemical shifts (δ) are expressed in ppm relative to the residual non-deuterated solvent reference ($CDCl_3$: ^{1}H 7.26 ppm, ^{13}C 77.16 ppm; THF-d_8: ^{1}H 1.72, 3.58 ppm, ^{13}C 67.31, 25.31 ppm; $(CD_3)_2CO$: 2.05 ppm, 29.84, 206.26 ppm).[1] The following abbreviations explained the multiplicities: s = singlet, d = doublet, t = triplet, m = multiplet. High resolution mass spectra were obtained on a Bruker Q-Tof Maxis II mass spectrometer and a DFS high resolution magnetic sector mass spectrometer. Accurate masses from high-resolution mass spectra were reported for the molecular ion $[M]^+$ and $[M+H]^+$. UV-vis-NIR absorption were recorded on an Agilent Cary 69 UV-vis-NIR spectrophotometer using 10 mm optical-path quartz cell at room temperature. Cyclic voltammetry measurements were analyzed on a CHI660E (CH Instruments, China) in a three-electrode cell. Electrolyte solutions were prepared by dissolving 0.1 M tetrabutylammoniumhexafluorophosphate ($n\mathrm{Bu_4NPF_6}$) and samples in anhydrous tetrahydrofuran (THF) and anhydrous dichloromethane (DCM), the experiments were performed with a scan rate of 100 mVs^{-1} at room temperature in nitrogen atmosphere. A glassy carbon and a Pt-sheet were used as working and counter electrode respectively. A silver wire was used as pseudo reference electrode. All potentials are given against ferrocenium/ferrocene (Fc^+/Fc) redox couple. Bis(triphenylphosphine)palladium(II) dichloride ($Pd(PPh_3)_2Cl_2$) were purchased from Energy-Chemical. Platinum(II) chloride ($PtCl_2$) were purchased from Macklin Reagent Inc. (Shanghai, China). Iron(Ⅲ) trichloride ($FeCl_3$) were purchased from Aladdin Scientific Corp. 1-ethynyl-4-hexylbenzene, 2-ethynyl-5-hexylthiophene was synthesized according to the reported procedures.[2,3] Other chemicals were purchased and used without further purification.

Synthetic details:

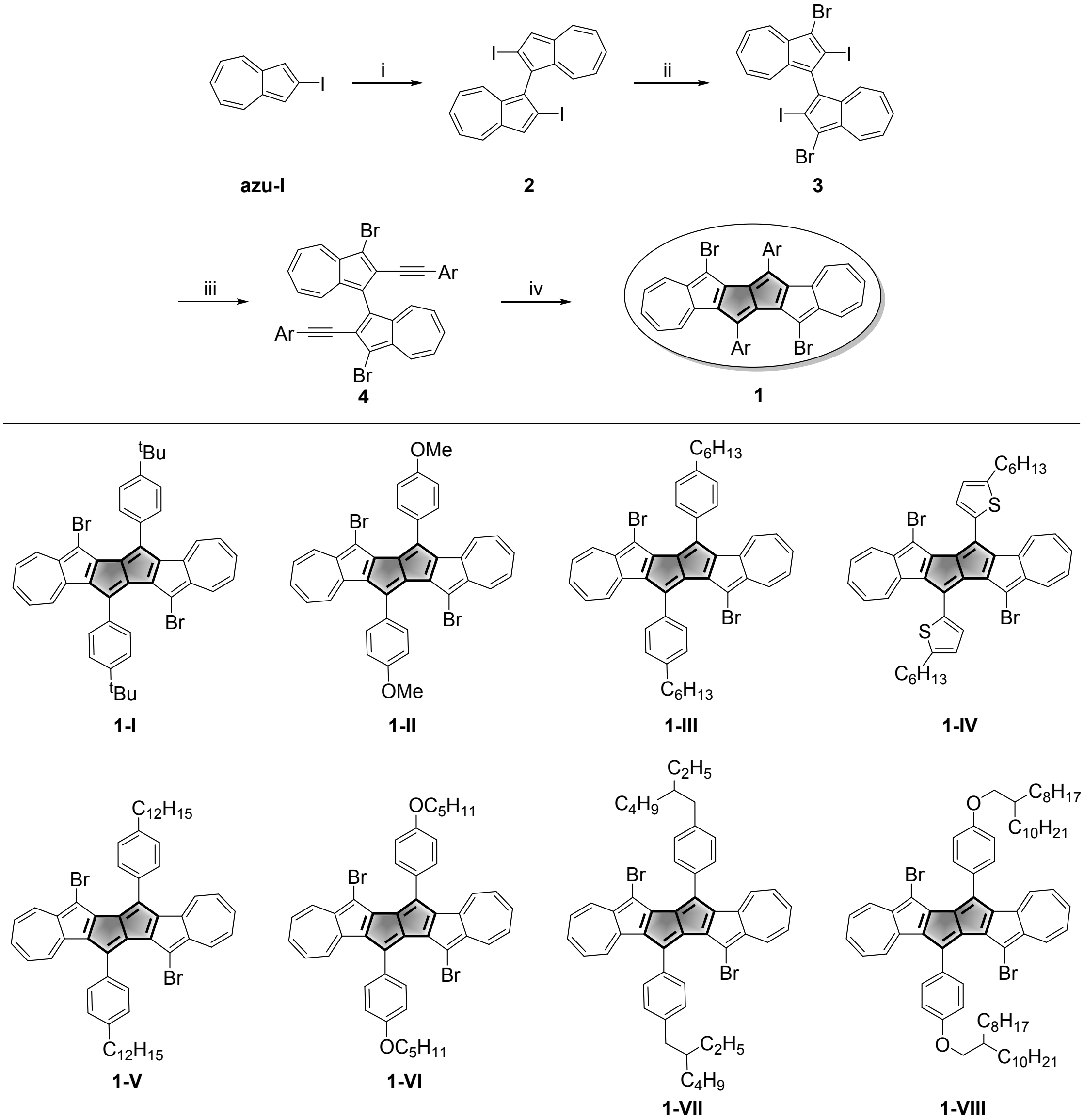

Scheme S1. The synthetic route to DAP derivatives. i) $FeCl_3$, DCM, -78 ^oC,30 min, 70%; ii) NBS, DCM, 0 ^oC, 30 min, 78%; iii) $Pd(PPh_3)_2Cl_2$, CuI, THF: Et_3N (5:1), rt, 1 h, 20-32%; iv) $PtCl_2$, o-xylene, 120 ^oC,4h, 8-12%. Abbreviations: DCM, Dichloromethane; NBS, N-Bromosuccinimide; THF, Tetrahydrofuran; Et_3N, Triethylamine; $Pd(PPh_3)_2Cl_2$, Bis(triphenylphosphine)palladium (II) chloride; $PtCl_2$, Platinum(II) chloride.

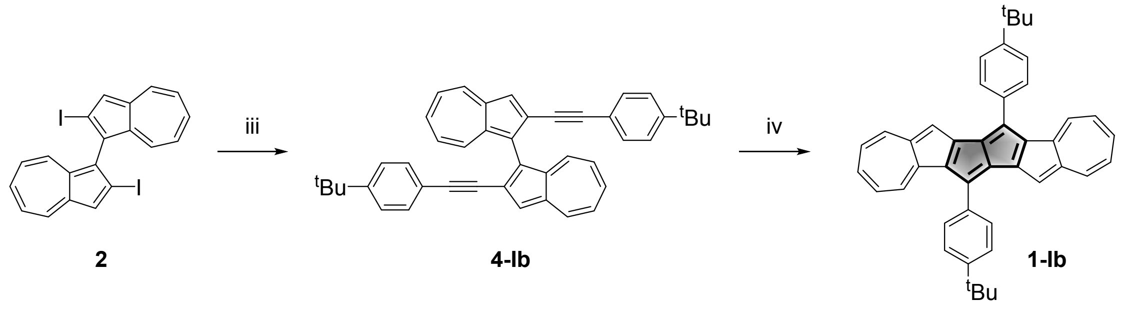

Scheme S2. The synthetic route to **1-Ib**. iii) $Pd(PPh_3)_2Cl_2$, CuI, THF: Et_3N (5:1), rt, 1 h, 30%; iv) $PtCl_2$, o-xylene, 120 ^oC,4h, 15%. Abbreviations: THF, Tetrahydrofuran; Et_3N, Triethylamine; $Pd(PPh_3)_2Cl_2$, Bis(triphenylphosphine)palladium (II) chloride; $PtCl_2$, Platinum(II) chloride.

Synthesis of compound **2**:

2-Iodoazulene (**azu-I**, 200.00 mg, 0.78 mmol) was dissolved in anhydrous DCM (20 mL) in a 100 mL Schlenk flask then the solution was cooled down to -78 °C. The solution of anhydrous iron (III) chloride (638.39 mg, 3.94 mmol) in dry nitromethane (2.5 mL) was added dropwise through a syringe at -78 °C in 5 min and the solution was stirred at -78 °C for 30 min under continuous nitrogen flow protection. Under the protection of nitrogen flow, methanol was added to the reaction solution for quenching and the solution was stirred for another 30 min. Poured into water and extracted with DCM. After drying with Na_2SO_4, the organic phase was evaporated under the reduced pressure. Then, the crude product was purified by silica gel column chromatography (hexane/DCM = 8/1) to obtain compound **2** 140mg (yield: 70%) as a blue solid.

^{1}H NMR (400 MHz, Chloroform-*d*) δ 8.37 (d, J = 9.6 Hz, 1H), 7.78 (s, 1H), 7.72 (d, J = 9.7 Hz, 1H), 7.65 (t, J = 9.8 Hz, 1H), 7.35 - 7.23 (m, 1H), 7.07 (t, J = 9.8 Hz, 1H).

^{13}C NMR (101 MHz, Chloroform-*d*) δ 140.99, 138.21, 138.08, 135.59, 135.32, 129.55, 124.62, 124.53, 124.24, 106.71.

MS (Maldi-TOF): $[M]^+$ calcd 505.90, found 505.94.

Synthesis of compound **3**:

Compound **2** (200 mg, 0.40 mmol) was dissolved in anhydrous DCM (10 mL) in a Schlenk bottle (100 mL), then the solution was cooled down to 0°C. N-Bromosuccinimide (NBS, 154.73 mg, 0.87 mmol) was added in portions under 0°C. The resulting mixture was stirred for 30 min at room temperature. After reaction, the mixture was washed with brine and water. The organic layer was dried over with anhydrous Na_2SO_4 and evaporated. The residue was purified by silica gel column

chromatography (hexane/DCM = 7/1) to give the compound **3** 205 mg (yield: 78 %) as green solid.

^{1}H NMR (400 MHz, Chloroform-*d*) δ 8.54 (dd, J = 9.9, 1.0 Hz, 1H), 7.76 - 7.63 (m, 2H), 7.42 - 7.32 (m, 1H), 7.17 - 7.07 (m, 1H).

^{13}C NMR (101 MHz, Chloroform-*d*) δ 139.17, 137.98, 136.36, 136.31, 136.04, 128.97, 125.30, 125.26, 112.28, 111.04.

MS (Maldi-TOF): $[M]^+$ calcd 663.72, found 663.75.

General Synthesis of compounds **4-I** to **4-VIII**:

To a Schlenk bottle (50 mL) was added compound **3** (100 mg, 0.15 mmol), ethynyl-Ar (0.75 mmol), Copper(I) iodide (CuI, 0.03 mmol). The resulting mixture was dissolved in anhydrous THF (10 mL) then degassed with N_2 for 0.5 h. Then Bis(triphenylphosphine)palladium(II) dichloride ($Pd(PPh_3)_2Cl_2$, 0.015 mmol) and Triethylamine (Et_3N, 2 mL) were added. The resulting mixture was stirred at room temperature under N_2 for 1 h. After reacting, the mixture was extracted with EtOAc and washed with brine and water. The organic layer was dried over with anhydrous Na_2SO_4 and evaporated. The residue was purified by silica gel column chromatography to give compound **4-I** to **4-VIII** as light green to yellowish green solid.

4-I: ^{1}H NMR (400 MHz, Chloroform-*d*) δ 8.42 (dd, J = 9.9, 0.9 Hz, 1H), 8.13 (dd, J = 9.8, 0.9 Hz, 1H), 7.58 (t, J = 9.8 Hz, 1H), 7.32 (t, J = 9.8 Hz, 1H), 7.22 (d, J = 8.5 Hz, 2H), 7.18 - 7.12 (m, 3H), 1.26 (s, 9H).

^{13}C NMR (101 MHz, Chloroform-*d*) δ 150.95, 137.90, 136.66, 136.25, 135.63, 135.21, 130.88, 130.45, 124.14, 124.05, 123.69, 123.54, 118.94, 106.20, 101.40, 85.28, 33.76, 30.07, 28.68.

HR-MS (Maldi-TOF): $[M]^+$ calcd 722.1178, found 722.1226.

4-II: ^{1}H NMR (400 MHz, Acetone-*d6*) δ 8.32 (dd, J = 9.9, 0.9 Hz, 1H), 8.02 (dd, J = 9.7, 0.9 Hz, 1H), 7.62 (tt, J = 9.9, 1.1 Hz, 1H), 7.35 (t, J = 9.8 Hz, 1H), 7.19 (t, J = 9.8 Hz, 1H), 7.03 - 6.97 (m, 2H), 6.71 - 6.66 (m, 2H), 3.66 (s, 3H).

^{13}C NMR (101 MHz, Acetone-*d6*) δ 160.42, 139.49, 137.62, 137.30, 136.66, 136.13, 133.13, 131.96, 125.30, 125.21, 124.79, 114.77, 114.15, 106.47, 102.62, 85.87, 54.89, 30.99.

HR-MS (Maldi-TOF): $[M]^+$ calcd 670.0138, found 670.0144.

4-III: ^{1}H NMR (400 MHz, Acetone-*d*6) *δ* 8.61 (dd, *J* = 9.8, 1.0 Hz, 1H), 8.31 (dd, *J* = 9.8, 0.9 Hz, 1H), 7.90 (tt, *J* = 10.0, 1.1 Hz, 1H), 7.62 (t, *J* = 9.8 Hz, 1H), 7.46 (t, *J* = 9.8 Hz, 1H), 7.29 - 7.22 (m, 4H), 2.76 (d, *J* = 7.8 Hz, 2H), 1.81 - 1.72 (m, 2H), 1.49 (s, 6H), 1.10 - 1.06 (m, 3H).

^{13}C NMR (101 MHz, Acetone-*d*6) *δ* 144.09, 139.59, 137.82, 137.39, 136.74, 136.42, 131.90, 131.74, 128.60, 125.32, 125.19, 125.05, 120.25, 107.24, 102.78, 86.73, 36.12, 32.02, 31.49, 30.07, 22.99, 14.15.

HR-MS (Maldi-TOF): $[M]^+$ calcd 778.1804, found 778.1754.

4-IV: ^{1}H NMR (400 MHz, Acetone-*d*6) *δ* 8.39 (dd, *J* = 10.0, 1.0 Hz, 1H), 8.06 (dd, *J* = 9.8, 1.0 Hz, 1H), 7.72 (tt, *J* = 9.8, 1.1 Hz, 1H), 7.44 (t, *J* = 9.7 Hz, 1H), 7.29 (t, *J* = 9.8 Hz, 1H), 6.82 (d, *J* = 3.7 Hz, 1H), 6.64 (dt, *J* = 3.6, 1.0 Hz, 1H), 2.72 - 2.66 (m, 2H), 1.52 (q, *J* = 7.2 Hz, 2H), 1.22 (d, *J* = 2.8 Hz, 6H), 0.80 (m, 3H).

^{13}C NMR (101 MHz, Acetone-*d*6) *δ* 149.91, 139.79, 137.69, 137.33, 136.71, 136.33, 132.97, 131.22, 125.56, 125.41, 124.93, 124.50, 120.04, 106.07, 95.89, 90.18, 31.52, 29.95, 22.56, 13.67.

HR-MS (Maldi-TOF): $[M]^+$ calcd 790.0933, found 790.0906.

4-V: ^{1}H NMR (500 MHz, Acetone-d_6) δ 8.48 (d, *J* = 9.4 Hz, 1H), 8.17 (d, *J* = 9.4 Hz, 1H), 7.78 (t, *J* = 9.8 Hz, 1H), 7.50 (t, *J* = 9.8 Hz, 1H), 7.35 (t, *J* = 9.8 Hz, 1H), 7.14 - 7.09 (m, 4H), 2.61 - 2.55 (m, 2H), 1.57 (t, *J* = 7.5 Hz, 2H), 1.30 (d, *J* = 9.9 Hz, 18H), 0.90 (t, *J* = 6.6 Hz, 3H).

^{13}C NMR (126 MHz, Acetone-d_6) δ 144.27, 139.77, 137.81, 137.31, 136.61, 136.36, 131.63, 131.45, 128.55, 125.38, 125.28, 119.98, 106.37, 102.26, 86.21, 35.63, 31.86, 31.16, 22.59, 13.62.

HR-MS (Maldi-TOF): $[M]^+$ calcd 948.3670, found 948.3655.

4-VI: ^{1}H NMR (500 MHz, Methylene Chloride-d_2) δ 8.46 (d, *J* = 10.8 Hz, 1H), 8.15 (d, *J* = 9.7 Hz, 1H), 7.65 (t, *J* = 9.9 Hz, 1H), 7.39 (t, *J* = 9.8 Hz, 1H), 7.21 (t, *J* = 9.8 Hz, 1H), 7.17 - 7.12 (m, 2H), 6.79 - 6.74 (m, 2H), 3.95 (t, *J* = 6.6 Hz, 2H), 1.82 - 1.74 (m, 2H), 1.42 (ddtd, *J* = 21.7, 14.9, 7.5, 7.1, 2.6 Hz, 4H), 0.95 (t, *J* = 7.0 Hz, 3H).

^{13}C NMR (126 MHz, Methylene Chloride-d_2) δ 159.84, 139.01, 137.50, 137.25, 136.65, 136.07, 133.12, 131.86 124.85, 124.79, 114.42, 106.56, 102.53, 85.64, 68.10, 28.80, 28.09, 22.41, 13.75.

HR-MS (Maldi-TOF): $[M]^+$ calcd 784.1374, found 784.1379.

4-VII: ^{1}H NMR (500 MHz, Acetone-d_6) δ 8.47 (d, J = 9.9 Hz, 2H), 8.17 (d, J = 9.9 Hz, 2H), 7.79 (t, J = 10.0 Hz, 2H), 7.51 (t, J = 9.8 Hz, 2H), 7.36 (t, J = 9.8 Hz, 2H), 7.12 (d, J = 8.3 Hz, 4H), 7.09 (d, J = 8.4 Hz, 4H), 2.50 (d, J = 7.1 Hz, 4H), 1.54 (p, J = 6.4, 5.8 Hz, 2H), 1.29 - 1.20 (m, 16H), 0.86 - 0.82 (m, 12H).

^{13}C NMR (151 MHz, Acetone-d_6) δ 144.30, 140.76, 138.77, 138.21, 137.47, 137.28, 132.42, 132.13, 130.23, 126.36, 126.24, 125.65, 120.77, 102.96, 41.78, 40.61, 32.98, 26.10, 23.63, 14.34, 11.00.

HR-MS (Maldi-TOF): $[M]^+$ calcd 836.2416, found 836.2535.

4-VIII: ^{1}H NMR (400 MHz, Acetone-d_6/CS_2 = 1/1) δ 8.45 (d, J = 9.8 Hz, 2H), 8.14 (d, J = 9.8 Hz, 2H), 7.76 (t, J = 9.8 Hz, 2H), 7.49 (t, J = 9.8 Hz, 2H), 7.33 (t, J = 9.8 Hz, 2H), 7.12 (d, J = 8.8 Hz, 4H), 6.82 (d, J = 8.8 Hz, 4H), 3.88 (d, J = 5.6 Hz, 4H), 1.76 (d, J = 7.9 Hz, 2H), 1.45 – 1.24 (m, 64H), 0.87 (t, J = 5.7 Hz, 12H).

^{13}C NMR (151 MHz, Acetone-d_6/CS_2 = 1/1) δ 161.04, 140.38, 138.44, 138.17, 136.94, 133.92, 126.19, 126.10, 115.55, 71.62, 38.67, 38.63, 32.68, 32.03, 30.74, 30.40, 27.51, 23.39, 14.43.

HR-MS (Maldi-TOF): $[M]^+$ calcd 1204.6077, found 1204.6141.

Synthesis of compounds **4-Ib**:

To a Schlenk bottle (50 mL) was added compound **2** (76 mg, 0.15 mmol), 4-tert-Butylphenylacetylene (0.75 mmol), Copper(I) iodide (CuI, 0.03 mmol). The resulting mixture was dissolved in anhydrous THF (10 mL) then degassed with N_2 for 0.5 h. Then Bis(triphenylphosphine)palladium(II) dichloride ($Pd(PPh_3)_2Cl_2$, 0.015 mmol) and Triethylamine (Et_3N, 2 mL) were added. The resulting mixture was stirred at room temperature under N_2 for 1 h. After reacting, the mixture was extracted with EtOAc and washed with brine and water. The organic layer was dried over with anhydrous Na_2SO_4 and evaporated. The residue was purified by silica gel column chromatography to give compound **4-Ib** as light green, yield:30%.

^{1}H NMR (500 MHz, Methylene Chloride-d_2) δ 8.41 (d, J = 9.2 Hz, 1H), 8.18 (d, J = 9.8 Hz, 1H), 7.72 (s, 1H), 7.61 (t, J = 9.8 Hz, 1H), 7.33 - 7.23 (m, 3H), 7.21 - 7.13 (m, 1H), 7.16 - 7.09 (m, 2H), 1.29 (s, 9H).

^{13}C NMR (126 MHz, Methylene Chloride-d_2) δ 151.78, 140.93, 138.05, 137.54, 137.22, 136.85, 131.51, 131.08, 125.24, 124.13, 123.97, 120.22, 120.00, 98.32, 87.88, 34.63, 30.81.

HR-MS (Maldi-TOF): $[M]^+$ calcd 566.2968, found 566.2956.

General Synthesis of compounds **1-I** to **1- VIII**:

To a Schlenk bottle (50 mL) was added compound **4-I** to **4- VIII** (0.10 mmol) and dissolved in anhydrous *o*-xylene (10 mL) then degassed with N_2 for 0.5 h. Then Platinum(II) chloride ($PtCl_2$, 0.60 mmol) and were added. The resulting mixture was stirred at 120℃ under N_2 for 4 h. After reacting, the mixture was filtered over Celite to remove Metal and concentrated in vacuo. The residue was purified by preparative TLC to give compound **1-I** to **1- VIII** as brown to orange solid.

1-I: ^{1}H NMR (400 MHz, Methylene Chloride-d_2) δ 7.62 (d, *J* = 8.5 Hz, 2H), 7.57 (d, *J* = 8.2 Hz, 2H), 7.53 (d, *J* = 9.4 Hz, 1H), 7.30 (d, *J* = 10.1 Hz, 1H), 6.95 (t, *J* = 9.8 Hz, 1H), 6.72 (t, *J* = 9.9 Hz, 1H), 6.50 (t, *J* = 10.0 Hz, 1H), 1.45 (s, 9H). The ^{13}C NMR spectrum could not be obtained due to the limited solubility (Figure S17).

HR-MS (Maldi-TOF): $[M]^+$ calcd 722.1178, found 722.1140.

1-II: ^{1}H NMR δ 7.61 – 7.54 (m, 4H), 7.49 (dd, *J* = 7.5, 3.9 Hz, 2H), 7.36 (d, *J* = 8.3 Hz, 1H), 7.26 (d, *J* = 8.1 Hz, 1H), 7.05 (d, *J* = 6.6 Hz, 4H), 6.96 – 6.89 (m, 2H), 6.73 – 6.65 (m, 2H), 6.50 – 6.40 (m, 2H), 3.90 (d, *J* = 1.6 Hz, 6H). The ^{13}C NMR spectrum could not be obtained due to the limited solubility (Figure S19).

HR-MS (Maldi-TOF): $[M]^+$ calcd 670.0138, found 670.0064.

1-III: ^{1}H NMR (400 MHz, Methylene Chloride-*d*2) *δ* 7.60 (dd, *J* = 8.2, 2.0 Hz, 2H), 7.58 - 7.50 (m, 1H), 7.41 - 7.33 (m, 3H), 6.95 (t, *J* = 9.8 Hz, 1H), 6.70 (t, *J* = 9.8 Hz, 1H), 6.48 (t, *J* = 9.9 Hz, 1H), 2.82 - 2.73 (m, 2H), 1.75 (p, *J* = 7.6 Hz, 2H), 1.42 (d, *J* = 14.4 Hz, 8H), 1.00 - 0.91 (m, 2H).

^{13}C NMR (151 MHz, THF-*d*8) *δ* 146.06, 143.41, 141.46, 140.00, 139.98, 137.78, 133.03, 132.97, 132.46, 132.25, 129.99, 127.53, 126.51, 126.25, 111.16, 35.69, 31.73, 31.27, 29.66, 28.88, 22.57, 13.46.

HR-MS (Maldi-TOF): $[M]^+$ calcd 778.1804, found 778.1792.

1-IV: ^{1}H NMR (600 MHz, THF-d_8) *δ* 7.64 (d, *J* = 10.3 Hz, 1H), 7.53 (d, *J* = 9.4 Hz, 1H), 7.16 (d, *J* = 3.4 Hz, 1H), 6.96 (t, *J* = 9.7 Hz, 1H), 6.89 (d, *J* = 3.5 Hz, 1H), 6.71 (t, *J* = 9.8 Hz, 1H), 6.53 (t, *J* = 9.9 Hz, 1H), 2.92 (t, *J* = 7.5 Hz, 2H), 1.79 - 1.75 (m, 2H), 1.45 (t, *J* = 7.5 Hz, 2H), 1.37 (td, *J* = 7.3, 6.4, 2.8 Hz, 4H), 0.94 - 0.91 (m, 3H).

^{13}C NMR (151 MHz, THF-d_8) *δ* 148.31, 146.91, 143.30, 141.79, 140.43, 138.90, 134.76, 134.19, 134.14, 133.34, 133.03, 130.92, 127.53, 127.33, 124.89, 112.38, 49.67, 32.44, 32.38, 30.70, 30.45, 29.45, 23.36, 14.25.

HR-MS (Maldi-TOF): $[M]^+$ calcd 790.0933, found 790.0996.

1-V: ^{1}H NMR (500 MHz, Acetone-d_6/CS_2 = 1/1) δ 7.58 (dd, J = 10.3, 7.7 Hz, 2H), 7.55 - 7.47 (m, 1H), 7.37 (dd, J = 9.3, 3.4 Hz, 3H), 7.00 (t, J = 9.8 Hz, 1H), 6.76 (q, J = 10.3, 9.9 Hz, 1H), 6.52 (q, J = 11.0, 9.8 Hz, 1H), 2.80 (t, J = 7.6 Hz, 2H), 1.79 (t, J = 7.4 Hz, 2H), 1.34 - 1.37 (m, 15H), 0.94 (t, J = 6.3 Hz, 6H).

^{13}C NMR (151 MHz, Acetone-d_6/CS_2 = 1/1) δ 147.88, 146.23, 143.51, 143.39, 141.68, 140.16, 138.12, 133.40, 133.29, 132.75, 132.10, 130.26, 127.79, 126.86, 126.66, 111.81, 36.14, 32.26, 31.71, 23.09, 14.19.

HR-MS (Maldi-TOF): $[M]^+$ calcd 948.3670, found 948.3756.

1-VI: ^{1}H NMR (600 MHz, Methylene Chloride-d_2) δ 7.61 (dd, J = 8.2, 6.6 Hz, 2H), 7.54 (d, J = 9.4 Hz, 1H), 7.34 (dd, J = 60.4, 10.2 Hz, 1H), 7.06 (d, J = 8.2 Hz, 2H), 6.95 (t, J = 9.8 Hz, 1H), 6.70 (t, J = 9.7 Hz, 1H), 6.49 (t, J = 9.8 Hz, 1H), 4.12 (t, J = 6.5 Hz, 2H), 2.22 (t, J = 7.6 Hz, 1H), 2.07 (q, J = 6.5 Hz, 2H), 1.93 (q, J = 7.1 Hz, 2H), 1.67 - 1.55 (m, 4H), 1.33 (d, J = 6.8 Hz, 17H), 1.05 (t, J = 7.2 Hz, 3H), 0.97 - 0.89 (m, 3H).

^{13}C NMR (151 MHz, Methylene Chloride-d_2) δ 159.81, 146.14, 143.49, 141.32, 140.37, 139.76, 137.70, 133.22, 133.20, 132.64, 131.71, 129.95, 126.84, 126.55, 126.40, 113.59, 111.68, 68.12, 35.78, 32.19, 30.07, 30.01, 29.92, 29.82, 29.66, 29.61, 29.51, 29.35, 28.56, 27.45, 25.70, 23.05, 22.90, 14.24, 14.19.

HR-MS (Maldi-TOF): $[M]^+$ calcd 784.1374, found 784.1455.

1-VII: ^{1}H NMR (500 MHz, Acetone-d_6/CS_2 = 1/1) δ 7.54 (d, J = 7.9 Hz, 4H), 7.47 (d, J = 9.4 Hz, 2H), 7.33 (d, J = 8.0 Hz, 4H), 7.23 (d, J = 10.2 Hz, 2H), 6.97 (t, J = 9.7 Hz, 2H), 6.75 (t, J = 9.8 Hz, 2H), 6.46 (t, J = 9.9 Hz, 2H), 2.71 (dq, J = 13.5, 6.3 Hz, 4H), 1.77 - 1.69 (m, 2H), 1.44 - 1.33 (m, 16H), 0.97 (dt, J = 13.5, 7.1 Hz, 12H).

^{13}C NMR (151 MHz, Acetone-d_6/CS_2 = 1/1) δ 148.56, 147.03, 143.02, 142.80, 142.23, 141.23, 138.71, 135.74, 134.17, 133.61, 133.03, 130.83, 129.26, 127.82, 127.47, 100.78, 42.14, 40.96, 33.35, 26.71, 24.17, 14.90, 11.69.

HR-MS (Maldi-TOF): $[M]^+$ calcd 836.2416, found 836.2415.

1-VIII: ^{1}H NMR (400 MHz, Acetone-d_6/CS_2 = 1/1) δ 7.62 – 7.51 (m, 4H), 7.53 – 7.46 (m, 2H), 7.38 (d, J = 10.2 Hz, 1H), 7.28 (d, J = 10.2 Hz, 1H), 7.08 – 7.01 (m, 4H), 7.01 – 6.93 (m, 2H), 6.79 – 6.68 (m, 2H), 6.50 (p, J = 9.8 Hz, 2H), 3.99 (dd, J = 5.6, 1.5 Hz, 4H), 1.87 (q, J = 5.9 Hz, 2H), 1.65 – 1.22 (m, 64H), 0.94 (td, J = 6.7, 4.0 Hz, 12H).

^{13}C NMR (101 MHz, Acetone-d_6) δ 160.68, 160.56, 148.47, 146.79, 144.20, 142.86, 142.06, 141.97, 141.00, 140.82, 138.59, 138.48, 135.58, 133.86, 133.56, 133.36,

132.36, 127.67, 127.37, 127.25, 114.32, 112.33, 100.84, 71.48, 38.99, 32.94, 32.40, 31.12, 30.75, 30.73, 30.71, 30.69, 27.96, 27.95, 23.78, 14.91, 14.89.

HR-MS (Maldi-TOF): $[M]^+$ calcd 1204.6077, found 1204.6159.

Synthesis of compounds **1-Ib**:

To a Schlenk bottle (50 mL) was added compound **4-Ib** (0.10 mmol) and dissolved in anhydrous *o*-xylene (10 mL) then degassed with N_2 for 0.5 h. Then Platinum(II) chloride ($PtCl_2$, 0.60 mmol) and were added. The resulting mixture was stirred at 120°C under N_2 for 4 h. After reacting, the mixture was filtered over Celite to remove Metal and concentrated in vacuo. The residue was purified by preparative TLC to give compound **1-Ib** as brown reddish solid, yield:15%.

^{1}H NMR (500 MHz, Methylene Chloride-d_2) δ 7.83 (d, *J* = 8.0 Hz, 2H), 7.73 (d, *J* = 10.3 Hz, 1H), 7.65 (d, *J* = 8.0 Hz, 2H), 7.57 (d, *J* = 9.3 Hz, 1H), 7.00 - 6.94 (m, 2H), 6.70 (t, *J* = 9.7 Hz, 1H), 6.61 (t, *J* = 9.8 Hz, 1H), 1.48 (s, 9H).

^{13}C NMR (151 MHz, Methylene Chloride-d_2) δ 152.60, 152.05, 148.40, 141.57, 141.09 , 140.58, 136.18, 135.18, 132.78, 132.74, 132.20, 128.61, 126.50, 125.59, 111.15, 34.83, 31.09, 29.68.

HR-MS (Maldi-TOF): $[M]^+$ calcd 566.2968, found 566.2996.

2. X-ray crystallographic analysis

Single crystals of **1-I** suitable for X-ray analysis were obtained by slow diffusion of methanol to its dichloromethane solution. These strong interactions further facilitate the formation of a 1D ladder-like stacking with around 3.05 Å between adjacent molecules (Figures S1). In addition to π-π interaction, each molecule **1-I** also engaged with four adjacent molecules through C-H·π interaction with distance around 2.83-2.98 Å and intermolecular van der Waals dispersion forces (*vdW* forces) with distance of 2.75 Å, eventually forming 2D network as shown in Figures S1.

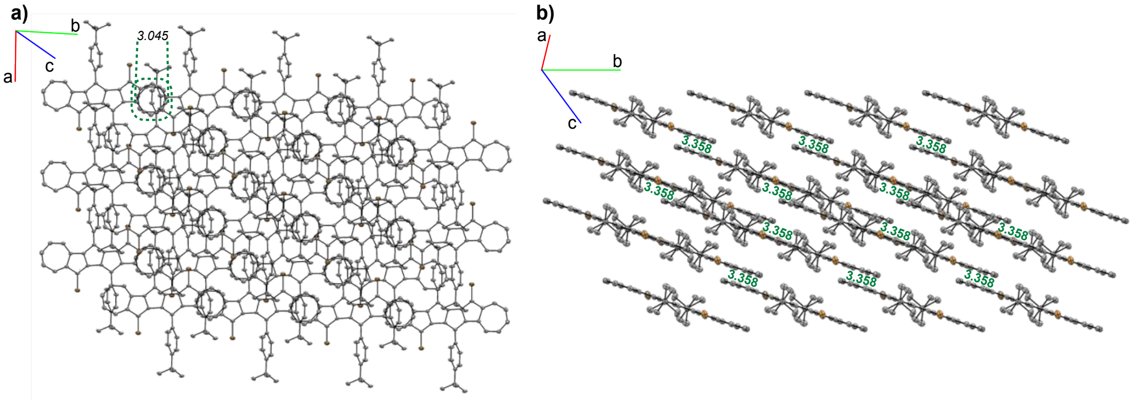

Figure S1. Single-crystal X-ray structures of compound **1-I** with ellipsoids at the 30% probability level. a) The packing styles of **1-I** at top view. b) The packing styles of **1-I** at side view.

Table S1. Crystallographic data and details of the structural refinements of **1-I**

	**1-I**
CCDC	2470360
Empirical formula	C44H36Br2
Formula weight	724.55
Temperature/K	223.00
Crystal system	triclinic
Space group	P-1
a/Å	8.8449(2)
b/Å	10.4576(3)

c/Å	10.6308(3)
α/°	61.4610(10)
β/°	78.041(2)
γ/°	84.189(2)
Volume/Å3	845.08(4)
Z	1
ρcalcg/cm3	1.424
μ/mm-1	3.263
F(000)	370.0
Crystal size/mm3	0.15 × 0.13 × 0.11
Radiation	CuKα (λ = 1.54178)
2Θ range for data collection/°	9.626 to 136.69
Index ranges	-10 ≤ h ≤ 10, -12 ≤ k ≤ 12, -12 ≤ l ≤ 11
Reflections collected	11377
Independent reflections	3089 [Rint = 0.0350, Rsigma = 0.0299]
Data/restraints/parameters	3089/108/287
Goodness-of-fit on F2	1.082
Final R indexes [I>=2σ (I)]	R1 = 0.0524, wR2 = 0.1619
Final R indexes [all data]	R1 = 0.0574, wR2 = 0.1680
Largest diff. peak/hole / e Å-3	0.63/-0.60

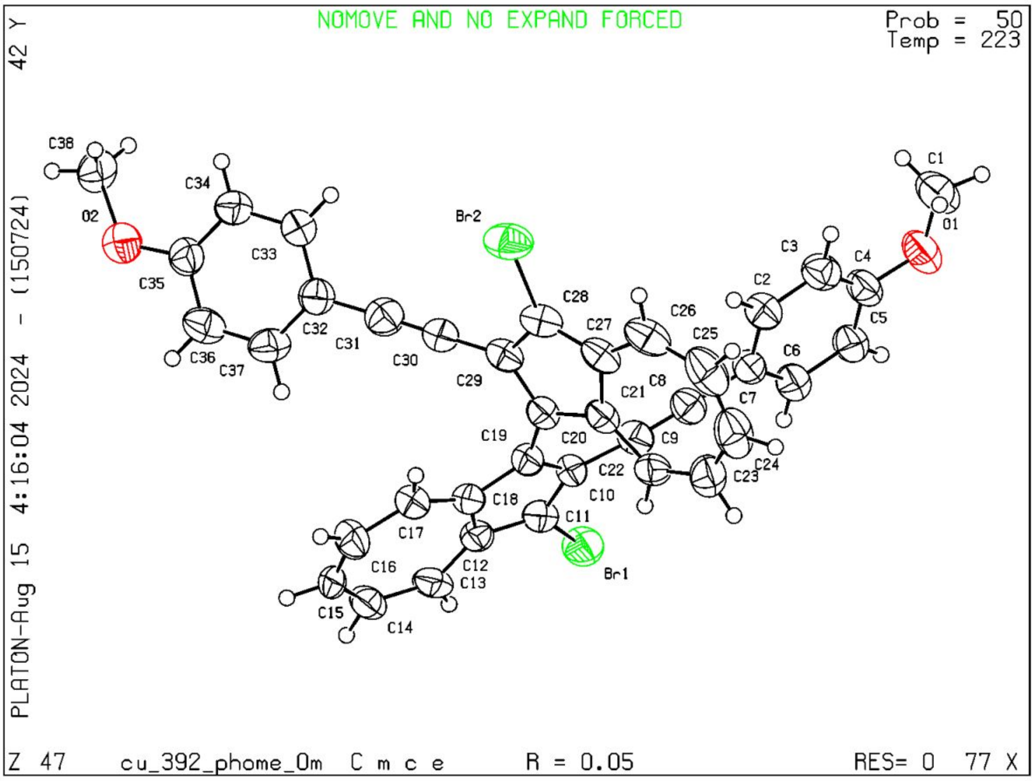

Figure S2. Single-crystal X-ray structures of compounds **4-II**.

Table S2. Crystallographic data and details of the structural refinements of **4-II**.

	**4-II**
CCDC	2470363
Empirical formula	C38H24Br2O2
Formula weight	672.39
Temperature/K	223.00
Crystal system	orthorhombic
Space group	Cmce
a/Å	6.9390(6)
b/Å	24.0422(18)

c/Å	35.635(2)
α/°	90
β/°	90
γ/°	90
Volume/Å3	5944.9(8)
Z	8
ρcalcg/cm3	1.502
μ/mm-1	3.719
F(000)	2704.0
Crystal size/mm3	0.13 × 0.12 × 0.11
Radiation	CuKα (λ = 1.54178)
2Θ range for data collection/°	4.96 to 137.666
Index ranges	0 ≤ h ≤ 8, 0 ≤ k ≤ 28, 0 ≤ l ≤ 43
Reflections collected	2991
Independent reflections	2991 [Rint = ?, Rsigma = 0.0537]
Data/restraints/parameters	2991/86/366
Goodness-of-fit on F2	0.989
Final R indexes [I>=2σ (I)]	R1 = 0.0472, wR2 = 0.1201
Final R indexes [all data]	R1 = 0.0701, wR2 = 0.1361
Largest diff. peak/hole / e Å-3	0.70/-0.37

3. Mechanism

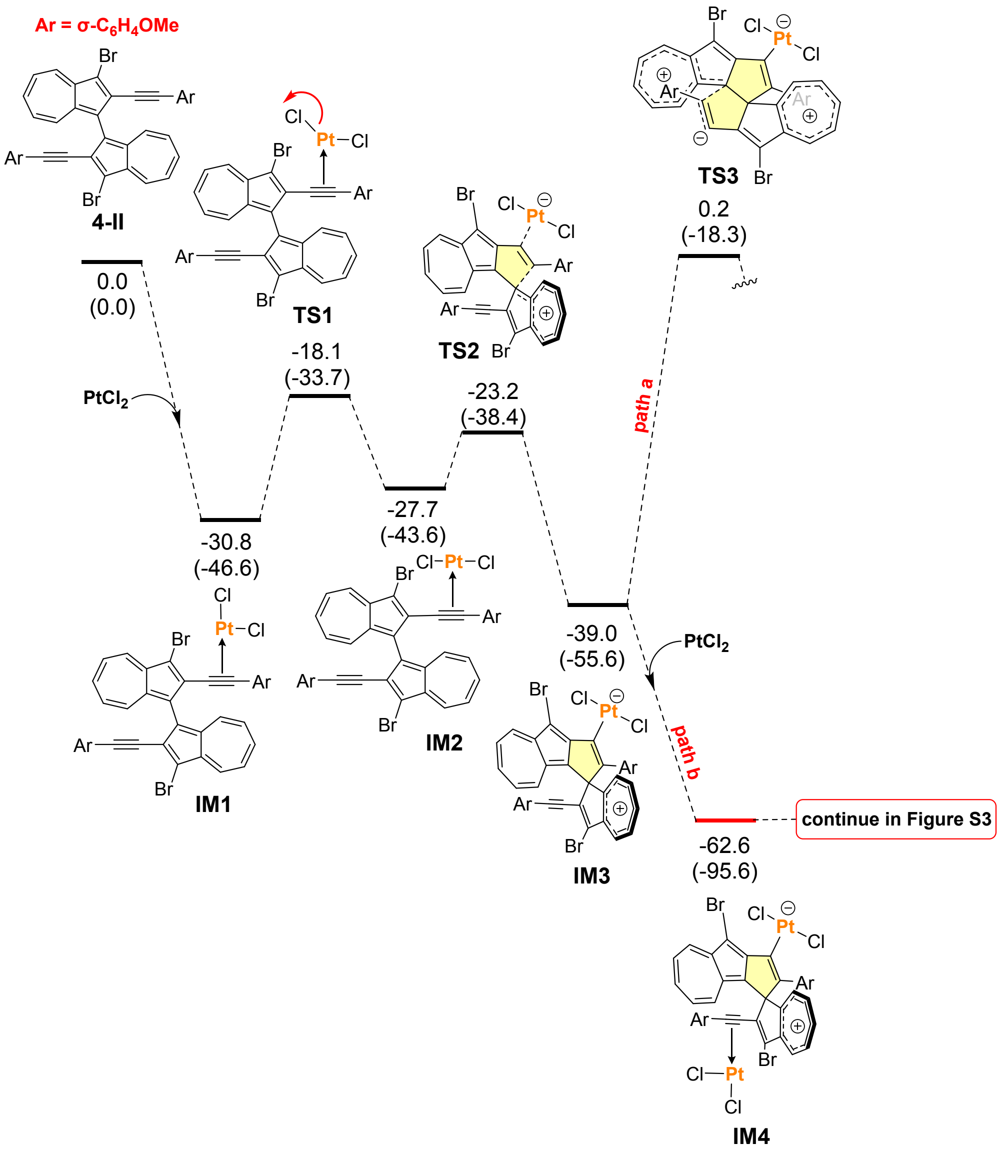

Figure S3. Detailed energy profile for the first cyclization step of the platinum(II)-catalyzed cascade reaction for substrate **4-II**. Relative Gibbs free energies and relative electronic energies (in parenthesis) are given in kcal/mol.

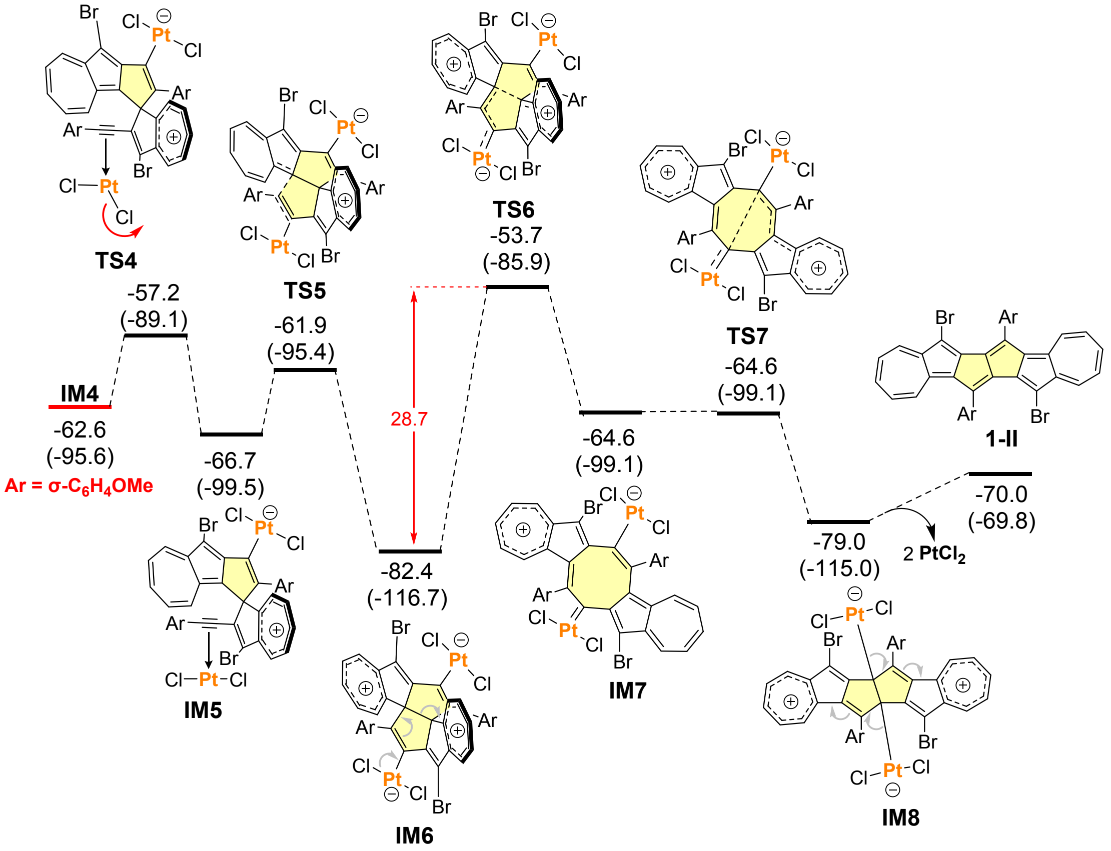

Figure S4. Detailed energy profile for the second cyclization step of the platinum(II)-catalyzed cascade reaction for substrate **4-II** starting from **IM4** to final product **1-II**. Relative Gibbs free energies and relative electronic energies (in parenthesis) are given in kcal/mol.

4. Additional CV

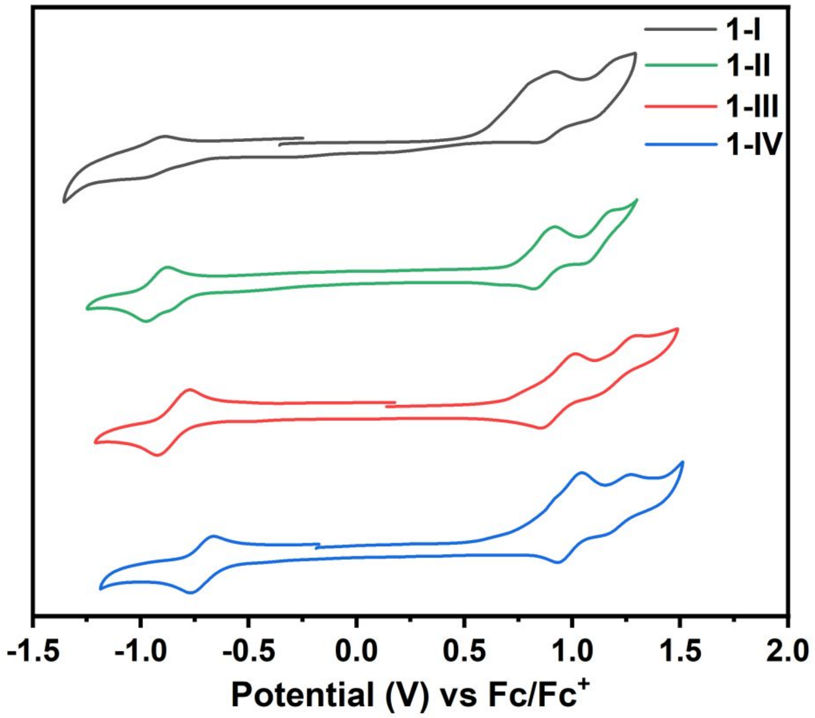

Figure S5. CV of diazulenopentalenes **1-I**, **1-II**, **1-III** and **1-IV** in THF.

Table S3. Summary of the electrochemical properties (obtained by CV) of derivatives **1I-1VIII**.

**Compound**	E_{LUMO}[a] (eV)	E_{HOMO}[b] (eV)	E_g^{CV}[c] (eV)
**1I**	–4.12	-5.57	1.45
**1II**	-4.20	-5.58	1.38
**1III**	-4.05	-5.60	1.55
**1IV**	-4.23	-5.58	1.35
**1V**	-4.05	-5.57	1.52
**1VI**	-4.01	-5.55	1.54
**1VII**	-4.00	-5.60	1.60
**1VIII**	-4.07	-5.59	1.52

[a] Calculated from E_{LUMO}= - (4.80+$E^{red}_{onset} - E_{Fc}$) (eV). [b] Calculated from E_{HOMO}= - (4.80+$E^{oxd}_{onset} - E_{Fc}$) (eV). [c] Calculated from $E^{gV} = E_{LUMO} - E_{HOMO}$ (eV).

5. NMR spectra and Mass spectra

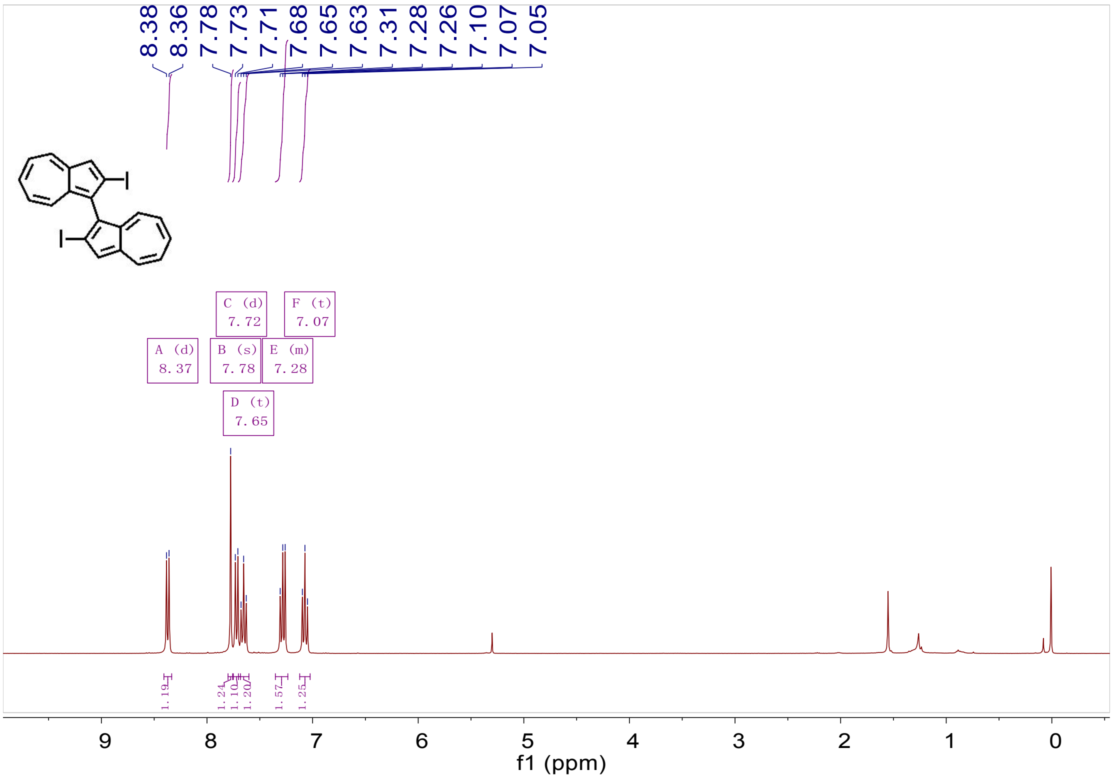

Figure S6. ^{1}H NMR of compound **2** in $CDCl_3$ at room temperature.

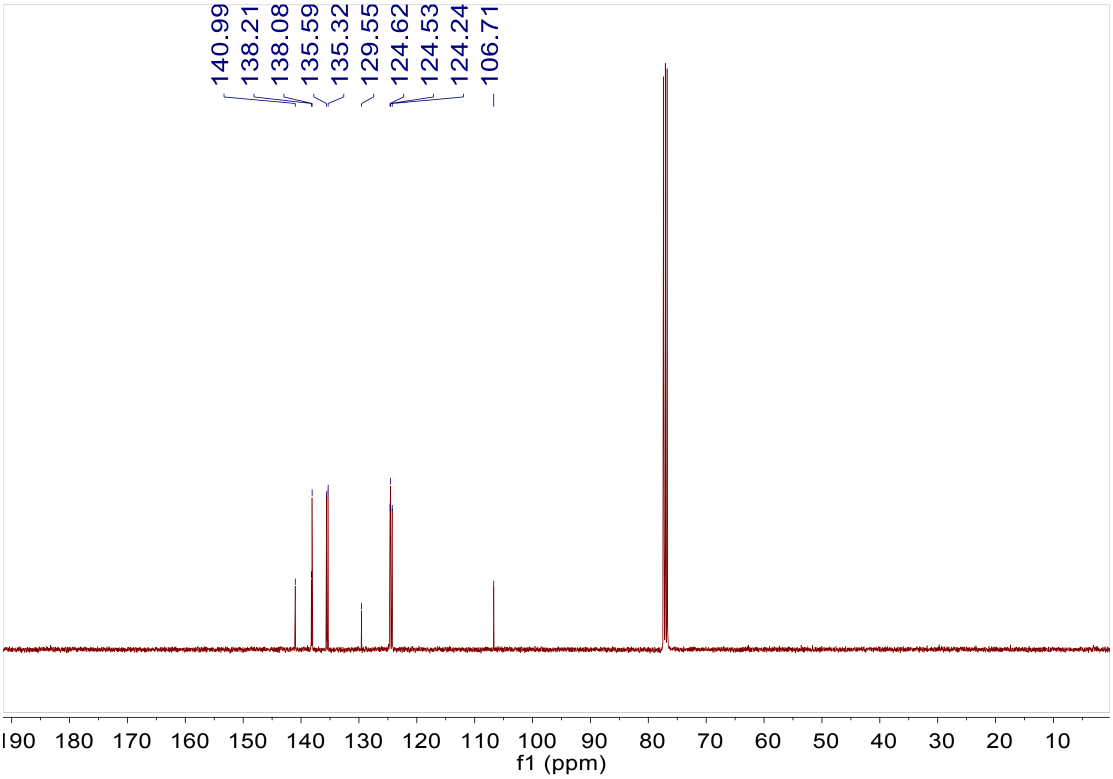

Figure S7. ^{13}C NMR of compound **2** in $CDCl_3$ at room temperature.

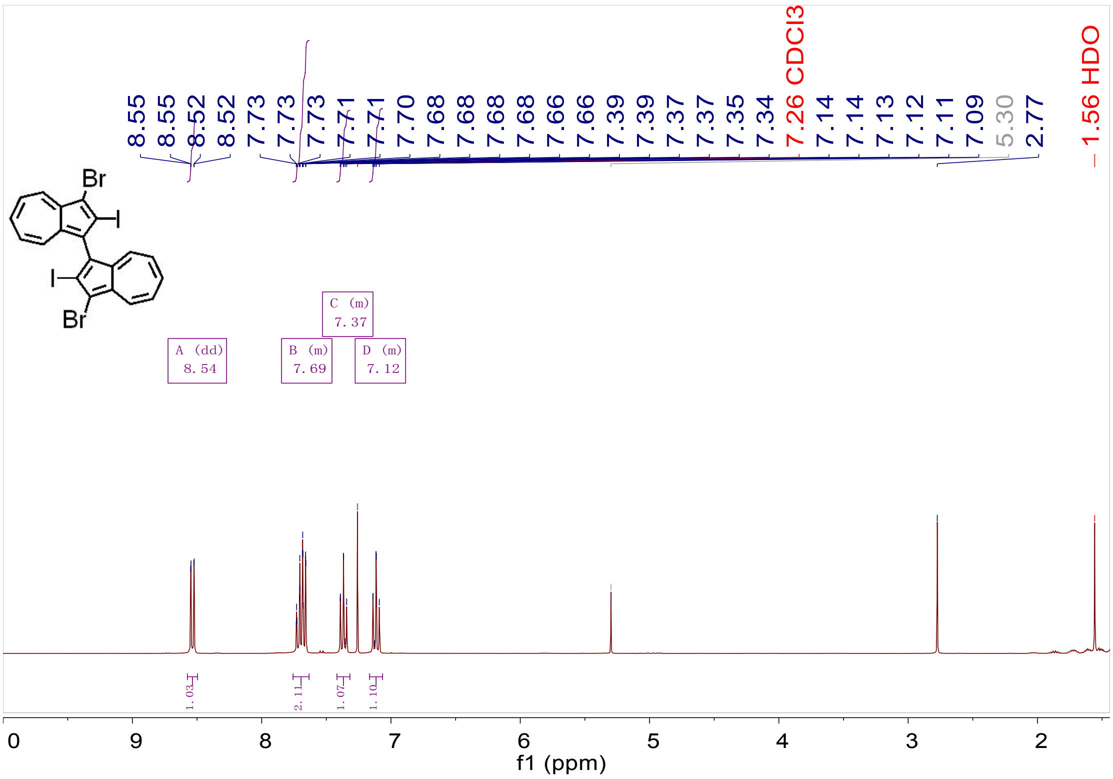

Figure S8. ^{1}H NMR of compound **3** in $CDCl_3$ at room temperature.

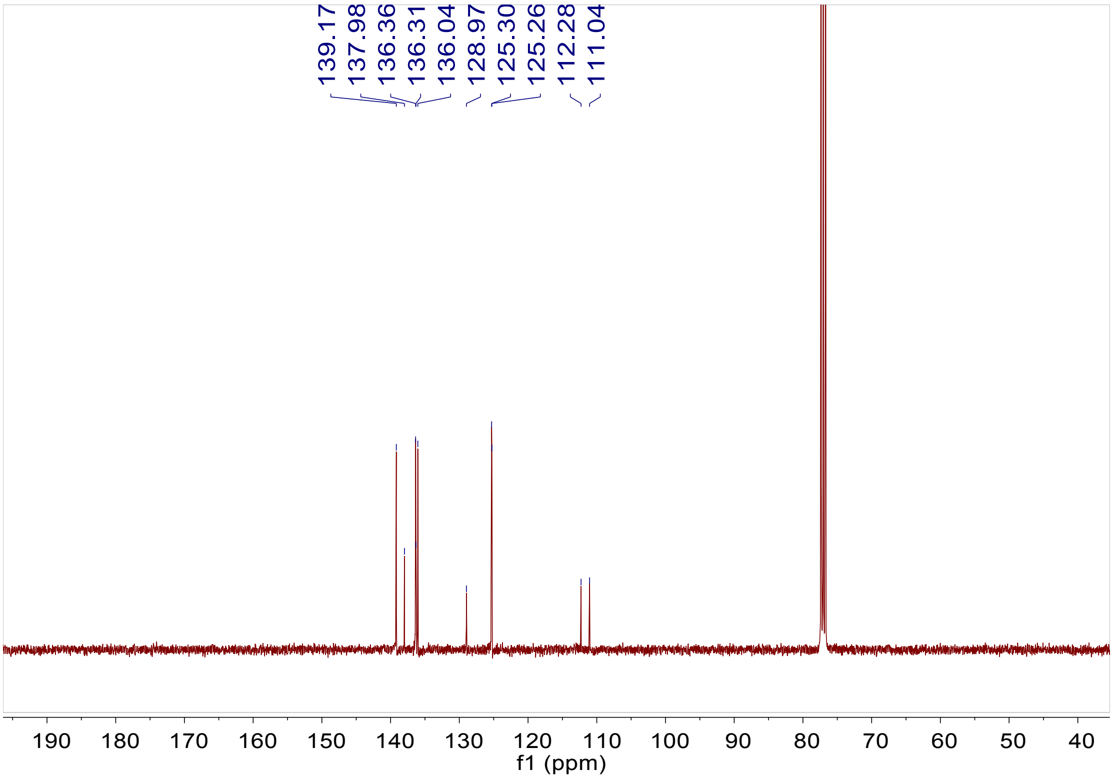

Figure S9. ^{13}C NMR of compound **3** in $CDCl_3$ at room temperature.

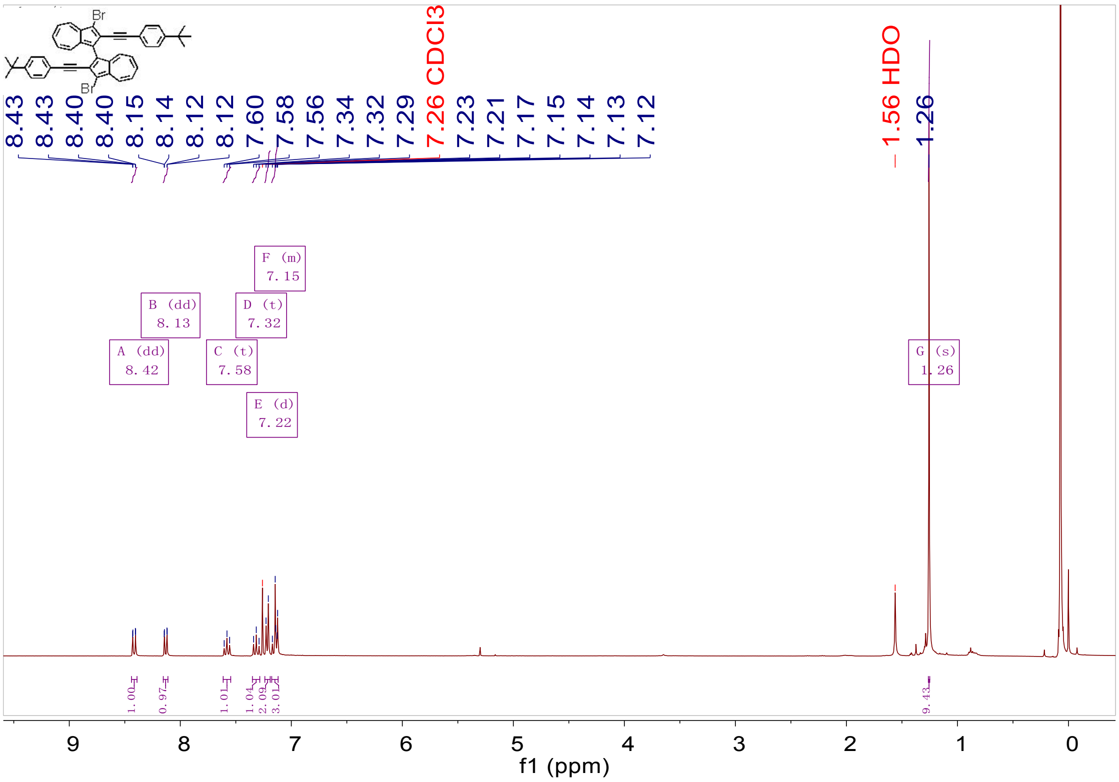

Figure S10. ^{1}H NMR of compound **4-I** in $CDCl_3$ at room temperature.

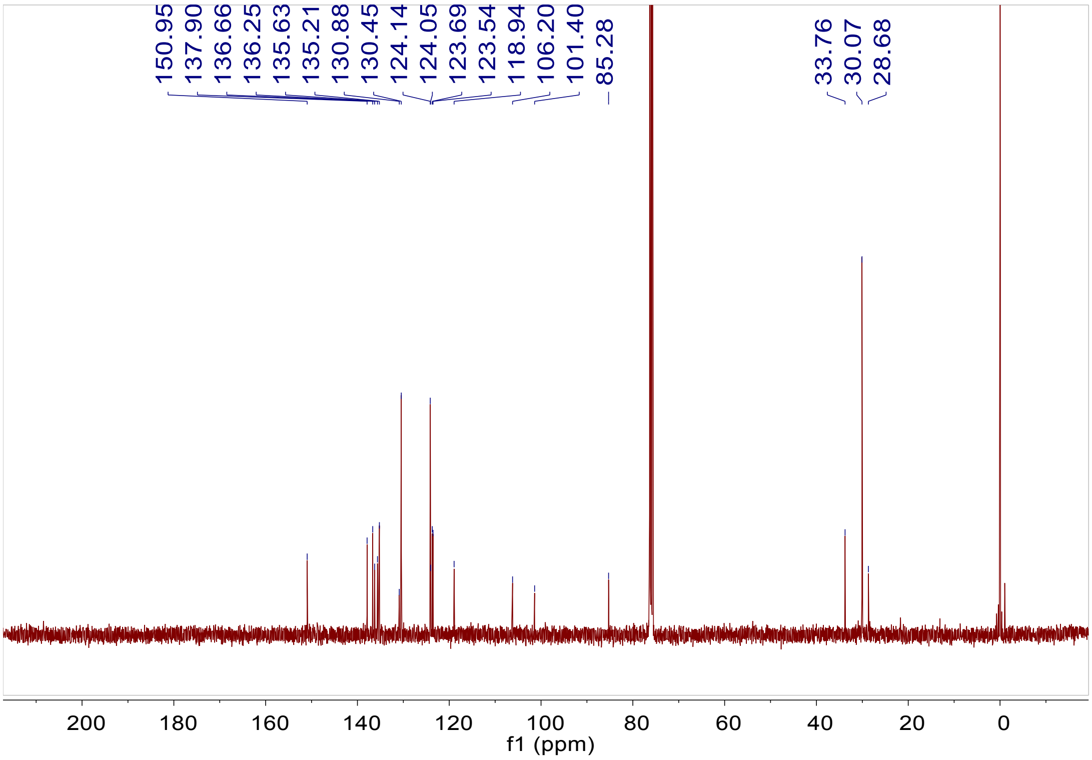

Figure S11. ^{13}C NMR of compound **4-I** in $CDCl_3$ at room temperature.

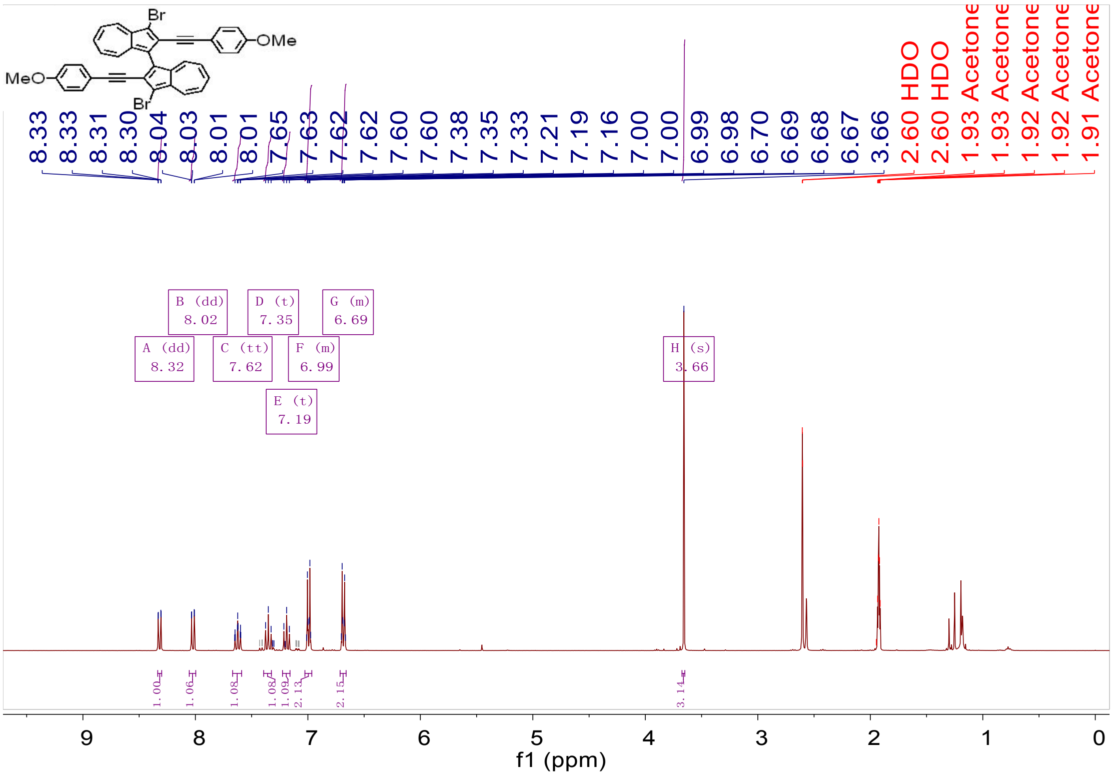

Figure S12. ^{1}H NMR of compound **4-II** in Acetone-d_6 at room temperature.

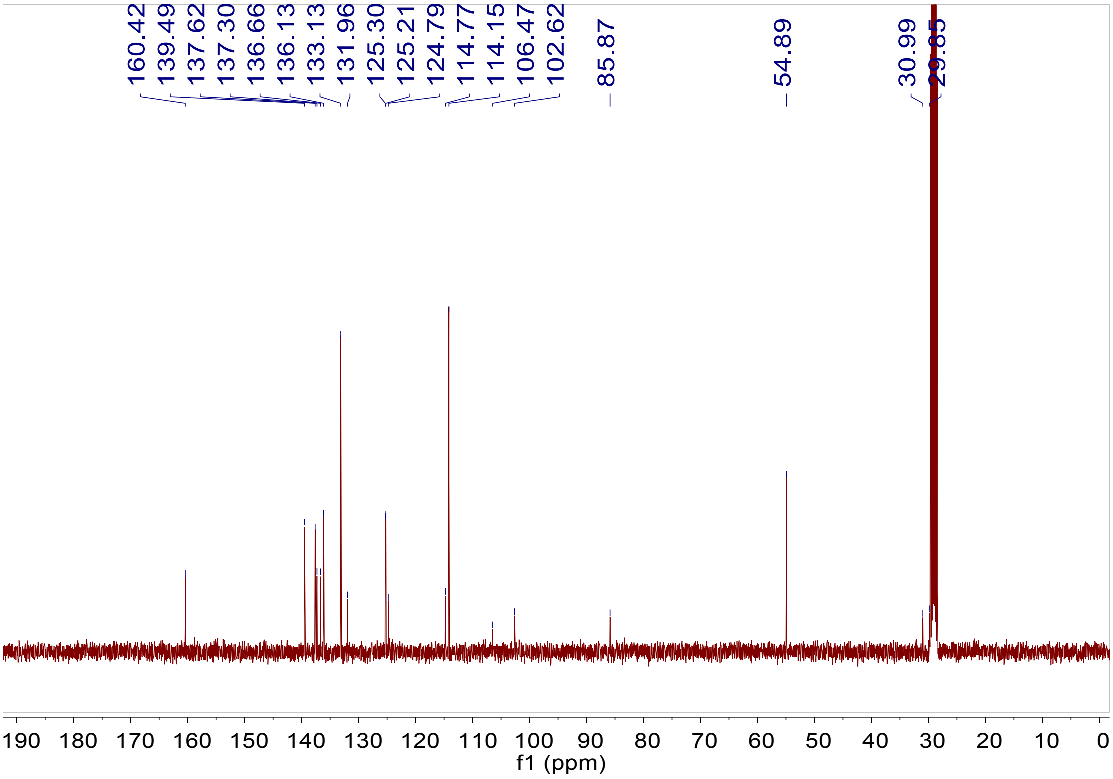

Figure S13. ^{13}C NMR of compound **4-II** in Acetone-d_6 at room temperature.

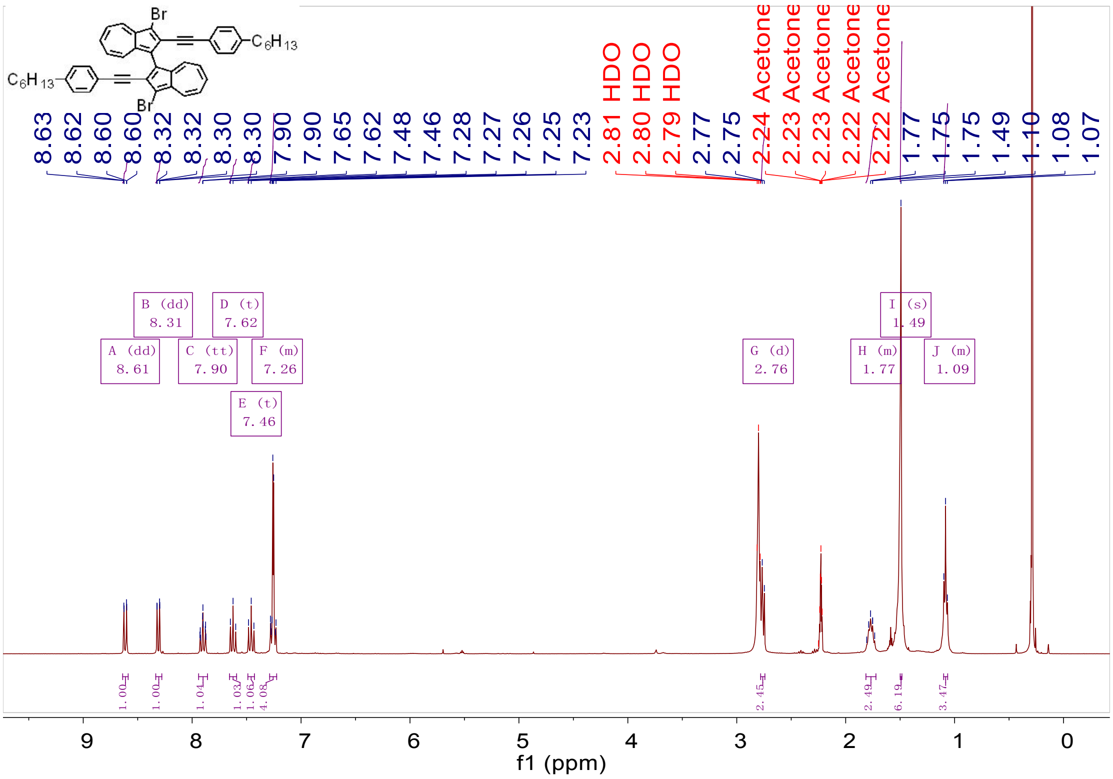

Figure S14. ^{1}H NMR of compound **4-III** in Acetone-d_6 at room temperature.

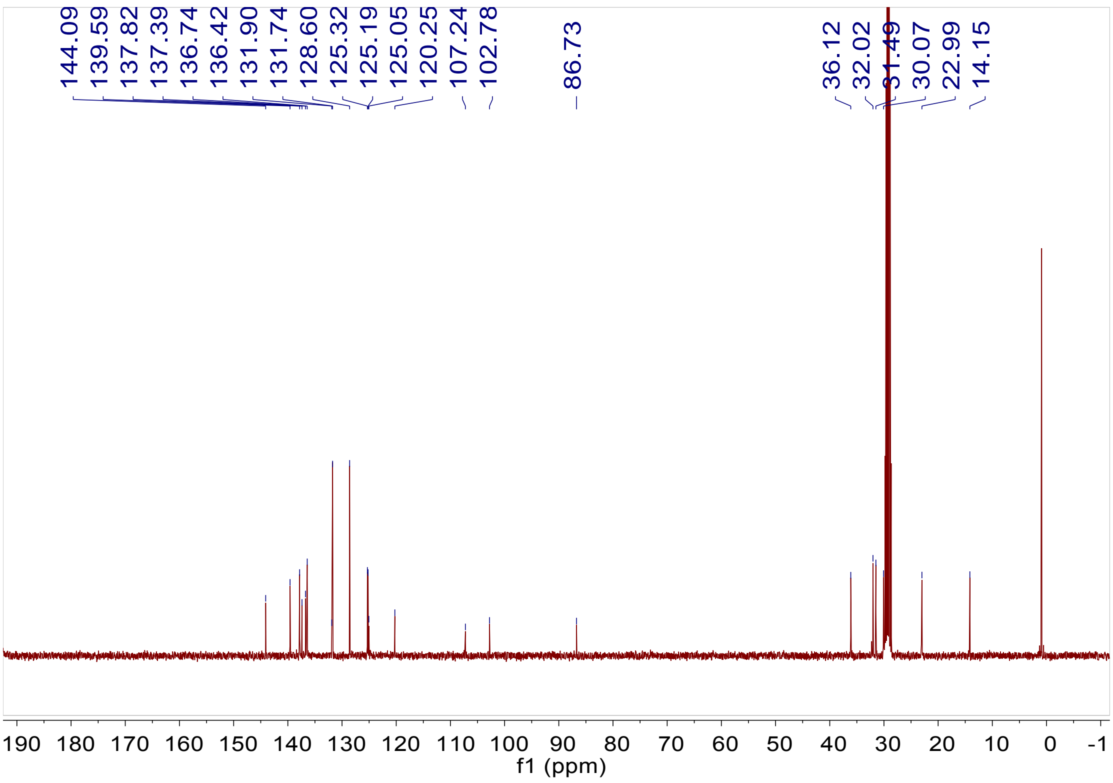

Figure S15. ^{13}C NMR of compound **4-III** in Acetone-d_6 at room temperature.

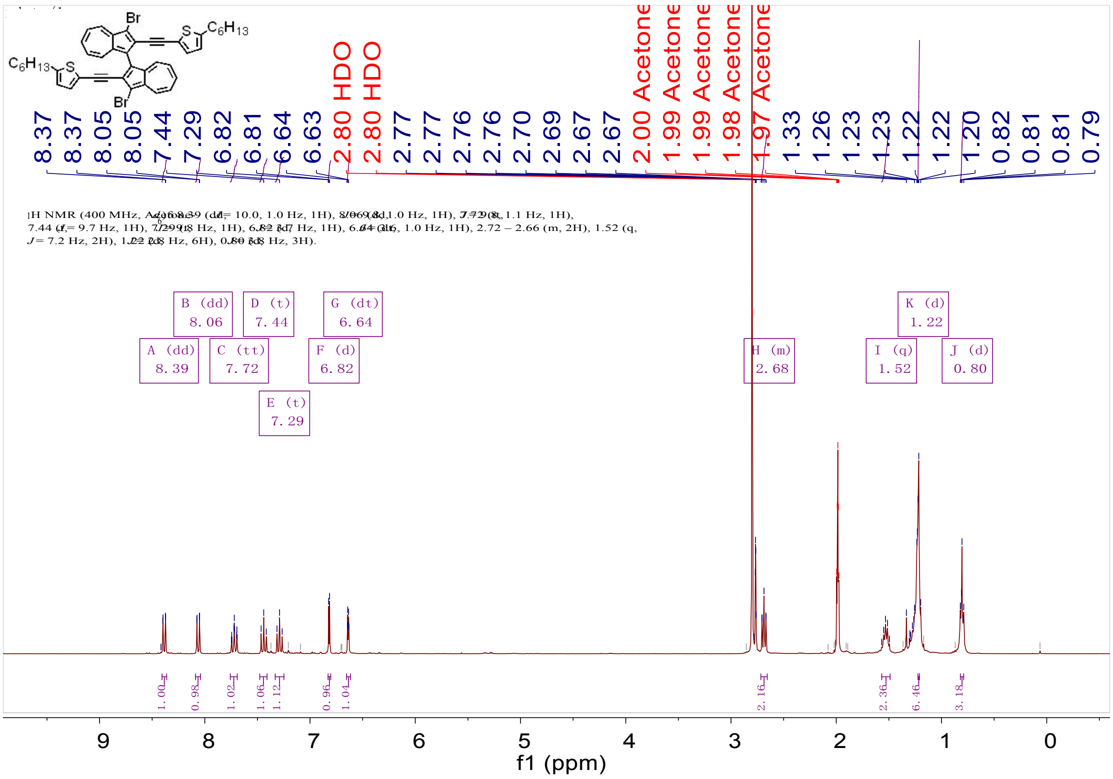

Figure S16. ^{1}H NMR of compound **4-IV** in Acetone-d_6 at room temperature**.**

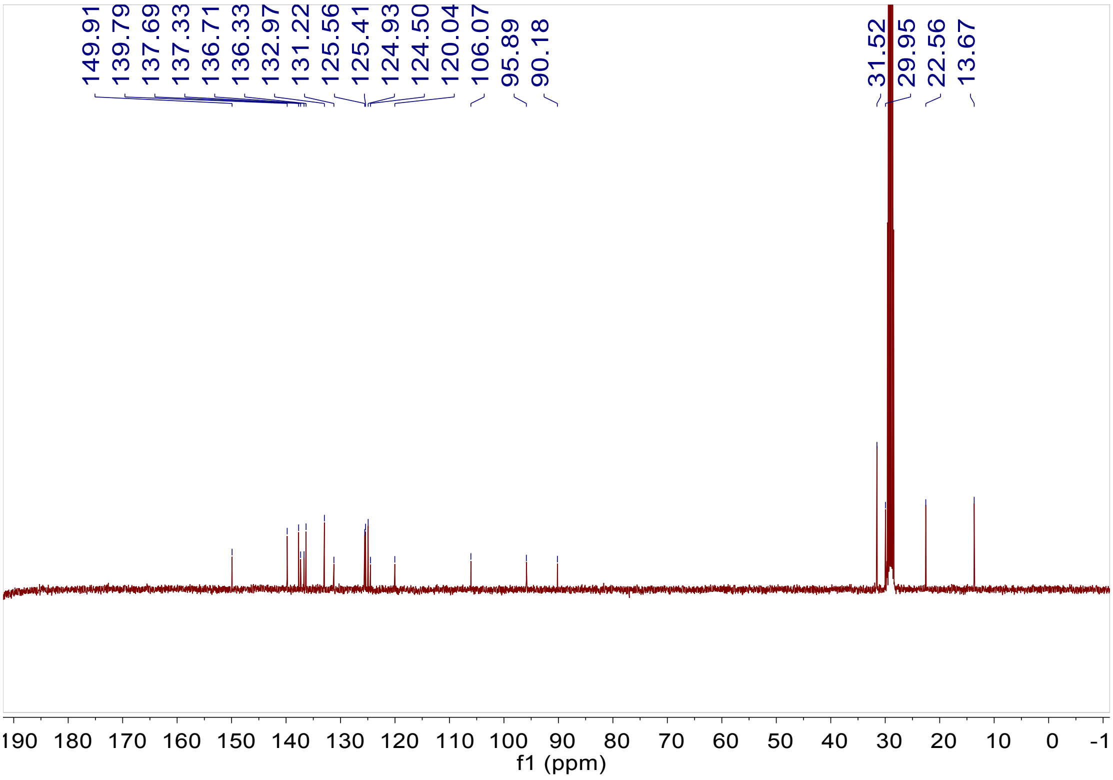

Figure S17. ^{13}C NMR of compound **4-IV** in Acetone-d_6 at room temperature.

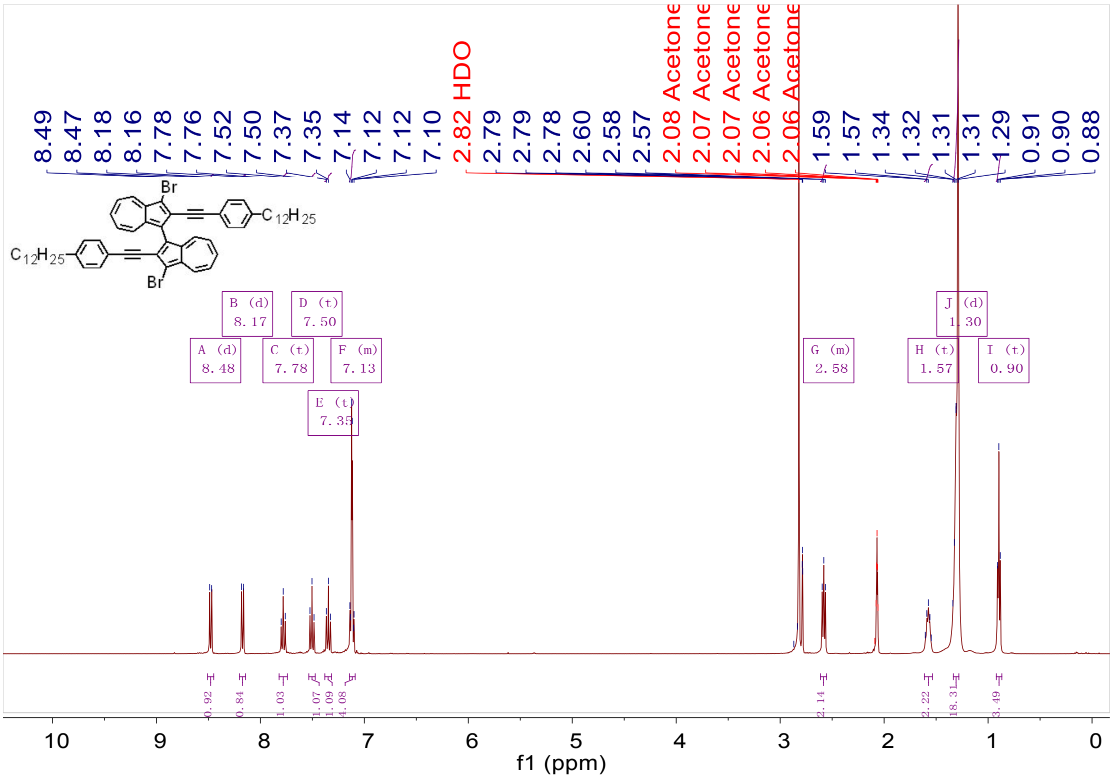

Figure S18. ^{1}H NMR of compound **4-V** in Acetone-d_6 at room temperature**.**

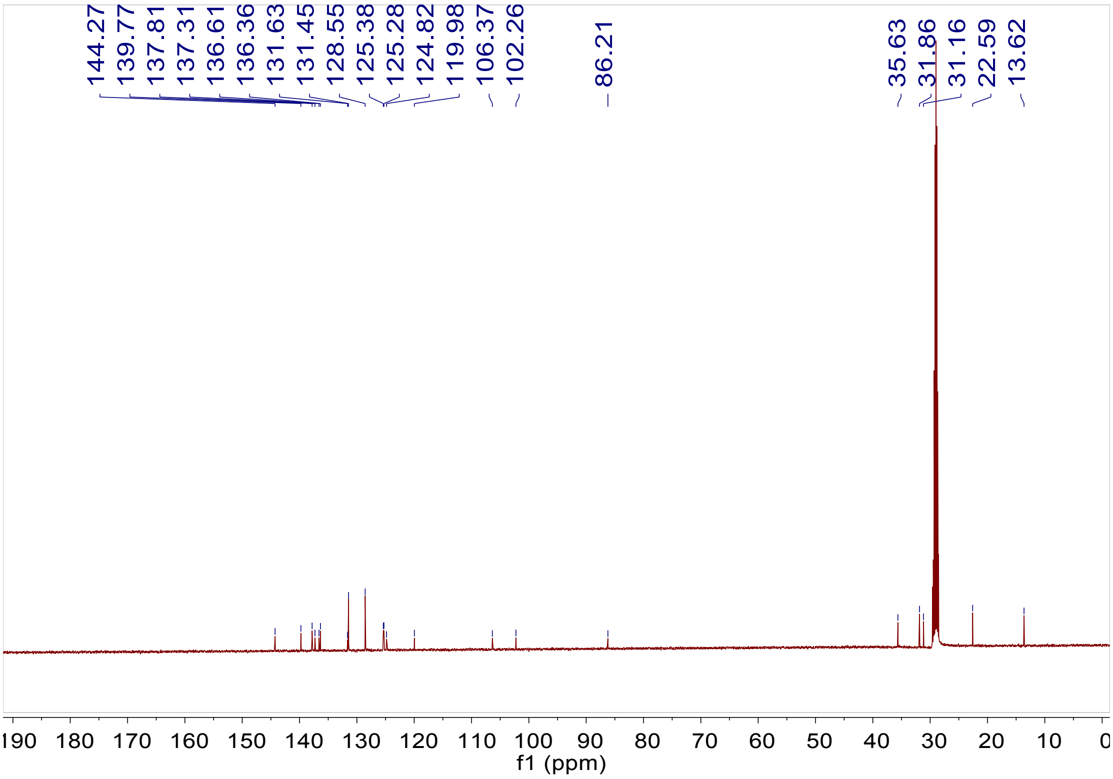

Figure S19. ^{13}C NMR of compound **4-V** in Acetone-d_6 at room temperature.

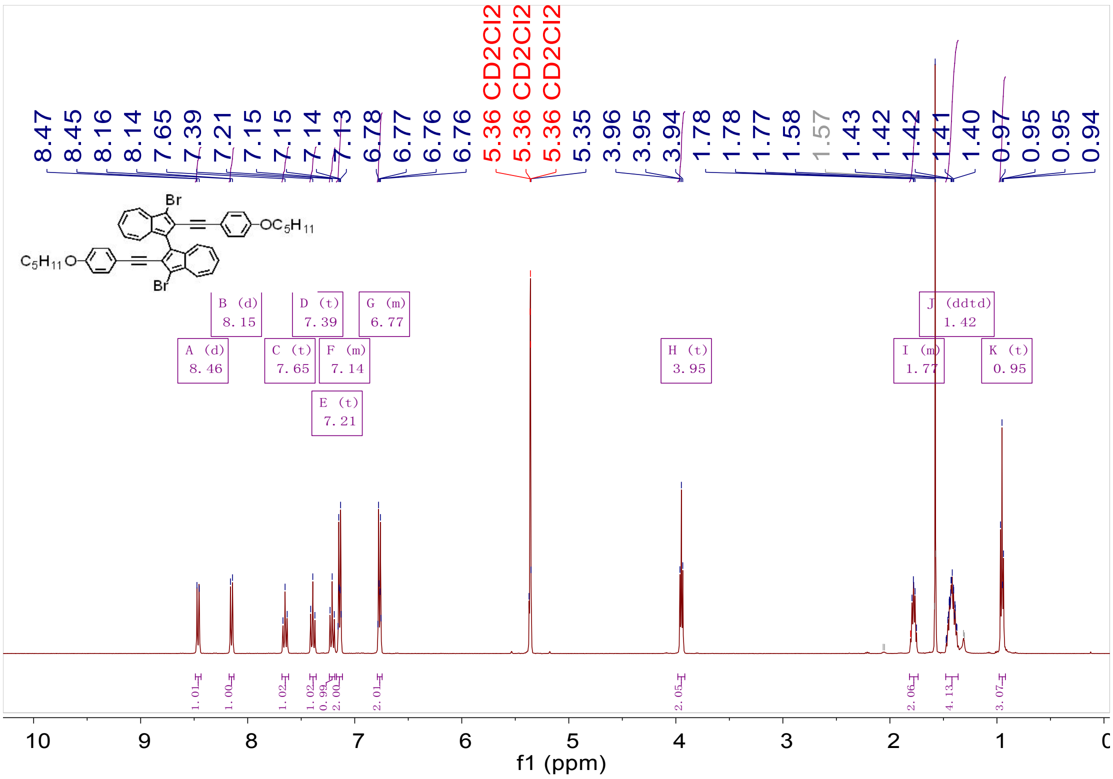

Figure S20. ^{1}H NMR of compound **4-VI** in CD_2Cl_2 at room temperature.

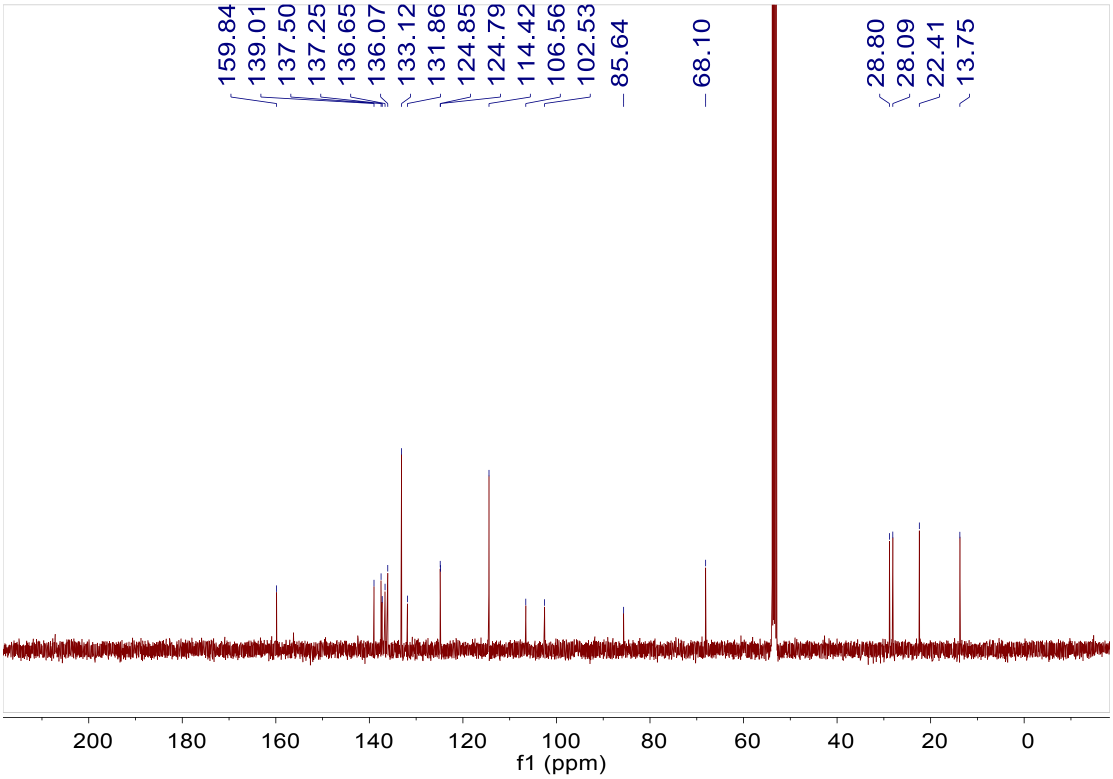

Figure S21. ^{13}C NMR of compound **4-VI** in CD_2Cl_2 at room temperature.

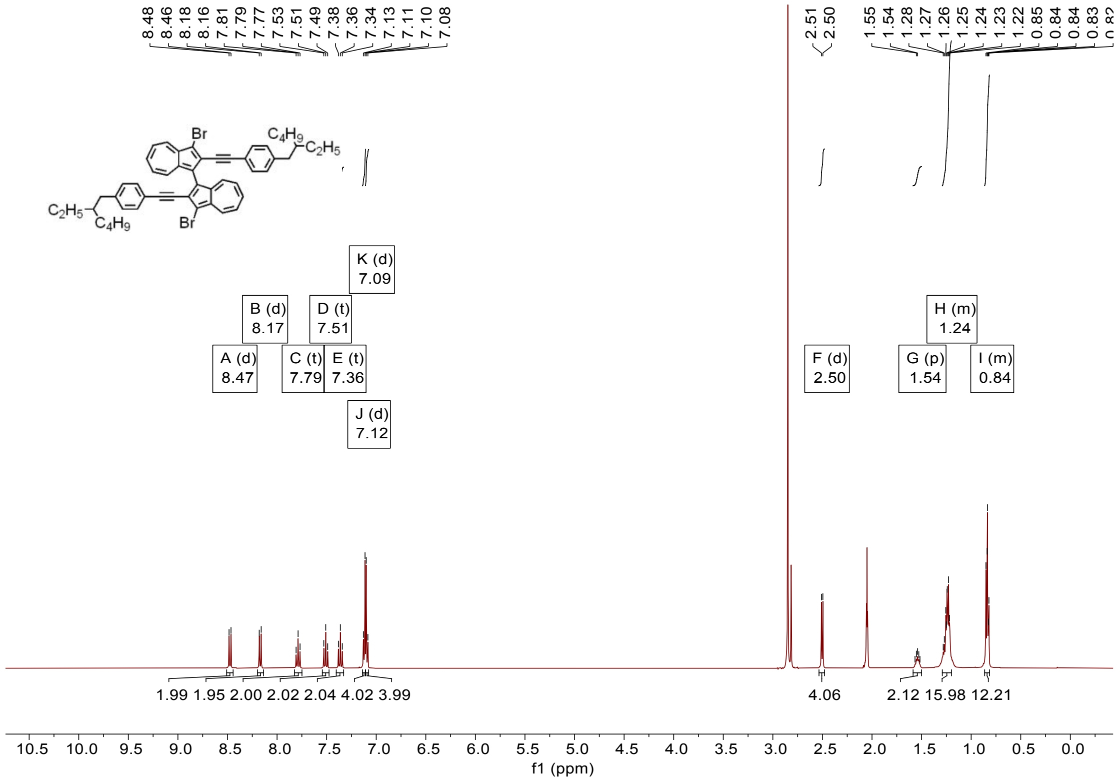

Figure S22. ^{1}H NMR of compound **4-VII** in Acetone-d_6 at room temperature.

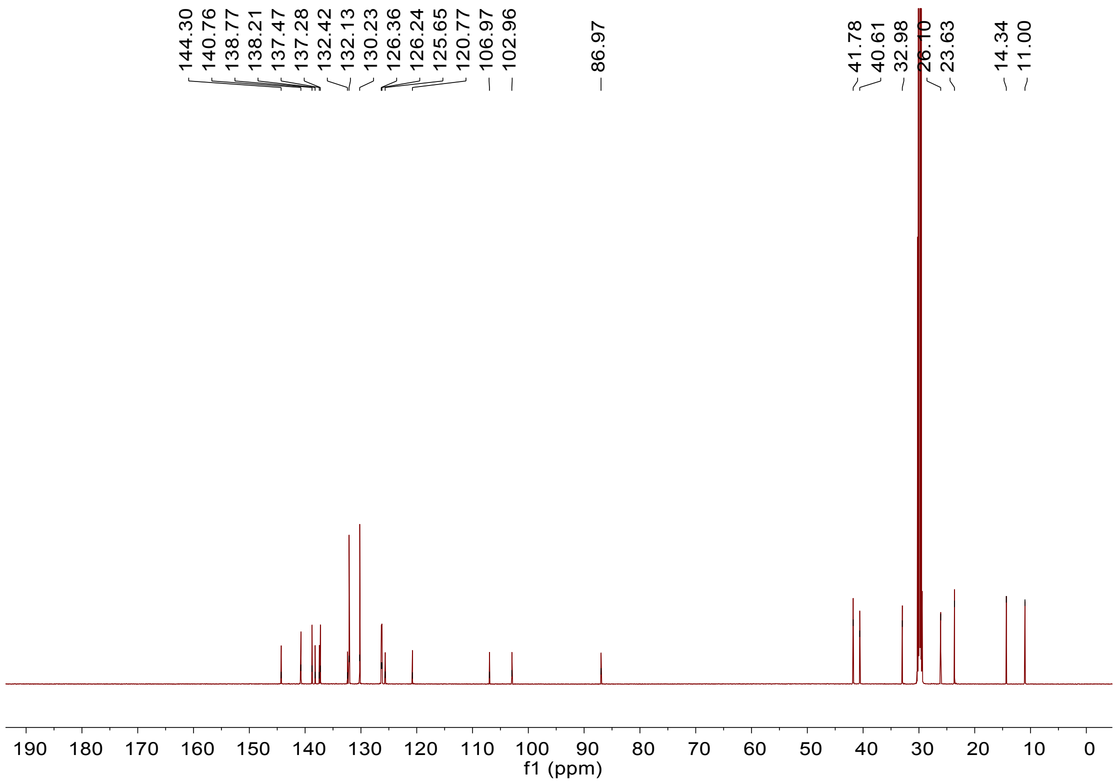

Figure S23. ^{13}C NMR of compound **4-VII** in Acetone-d_6 at room temperature.

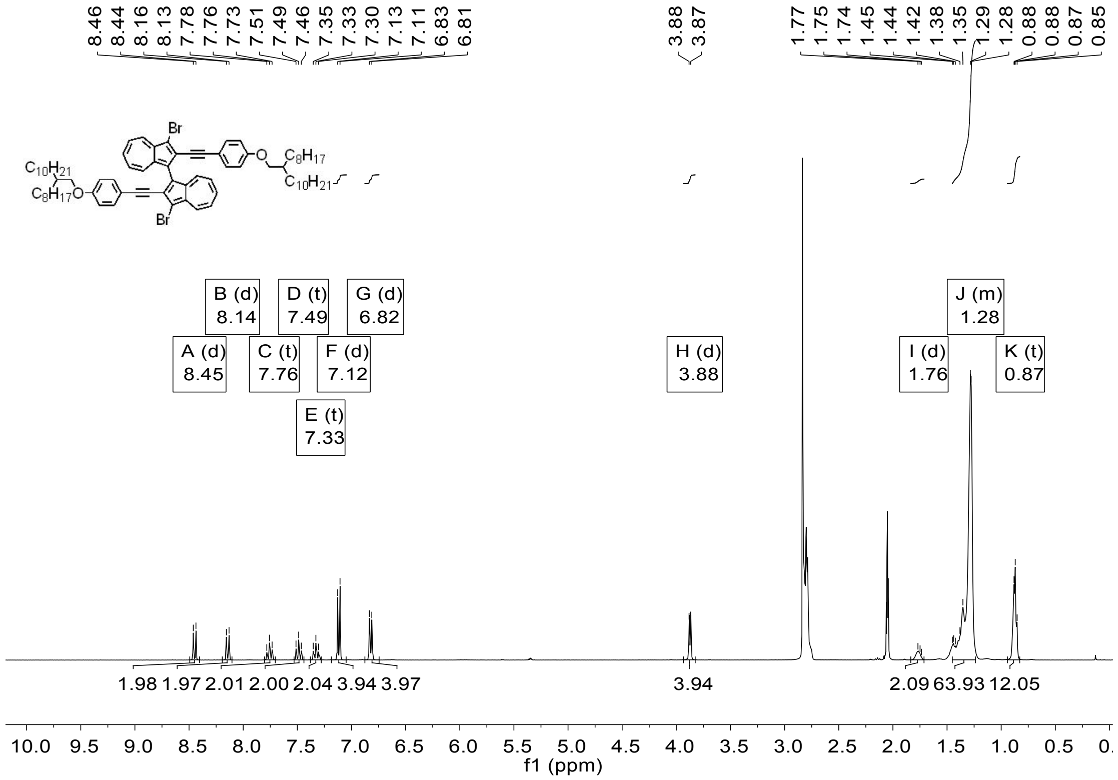

Figure S24. ^{1}H NMR of compound **4-VIII** in Acetone-d_6/CS_2 (1/1) at room temperature.

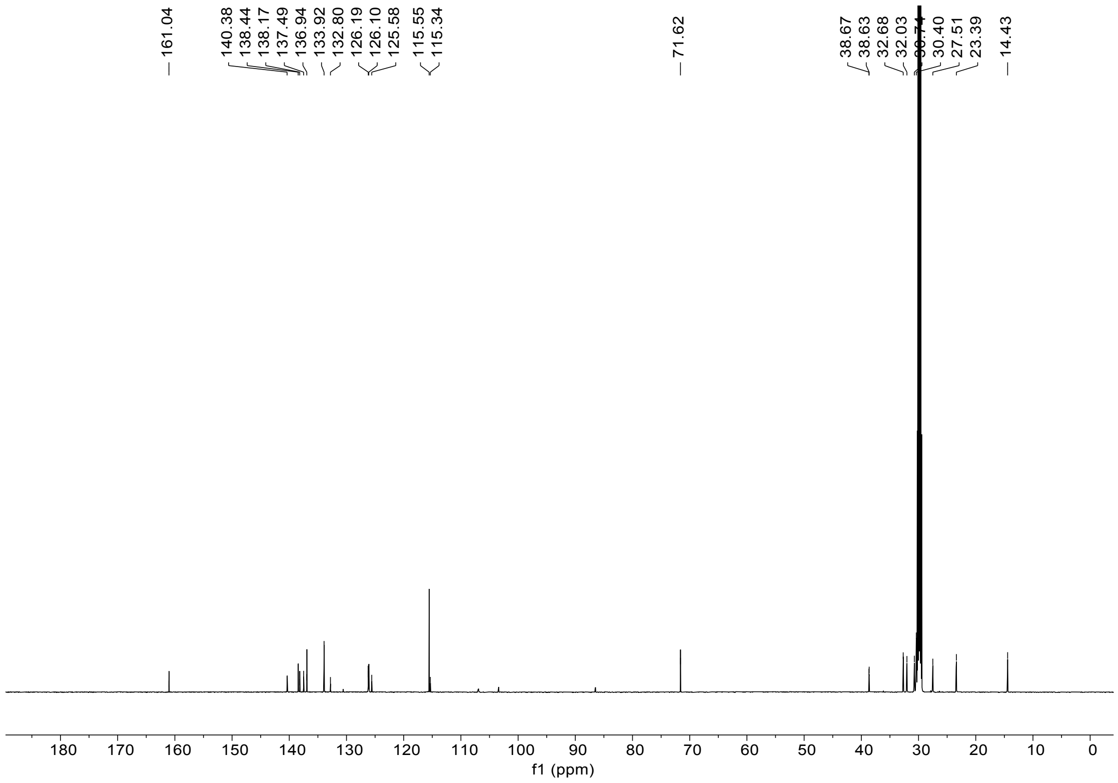

Figure S25. ^{13}C NMR of compound **4-VIII** in Acetone-d_6/CS_2 (1/1) at room temperature.

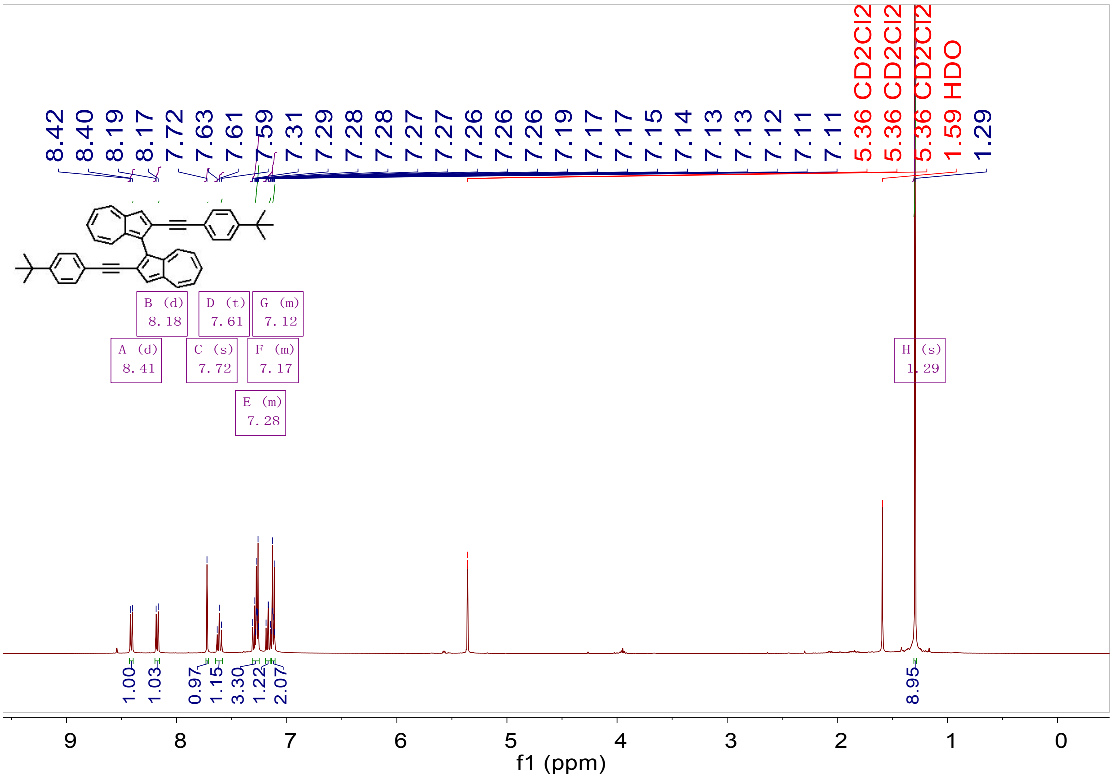

Figure S26. ^{1}H NMR of compound **4-Ib** in CD_2Cl_2 at room temperature.

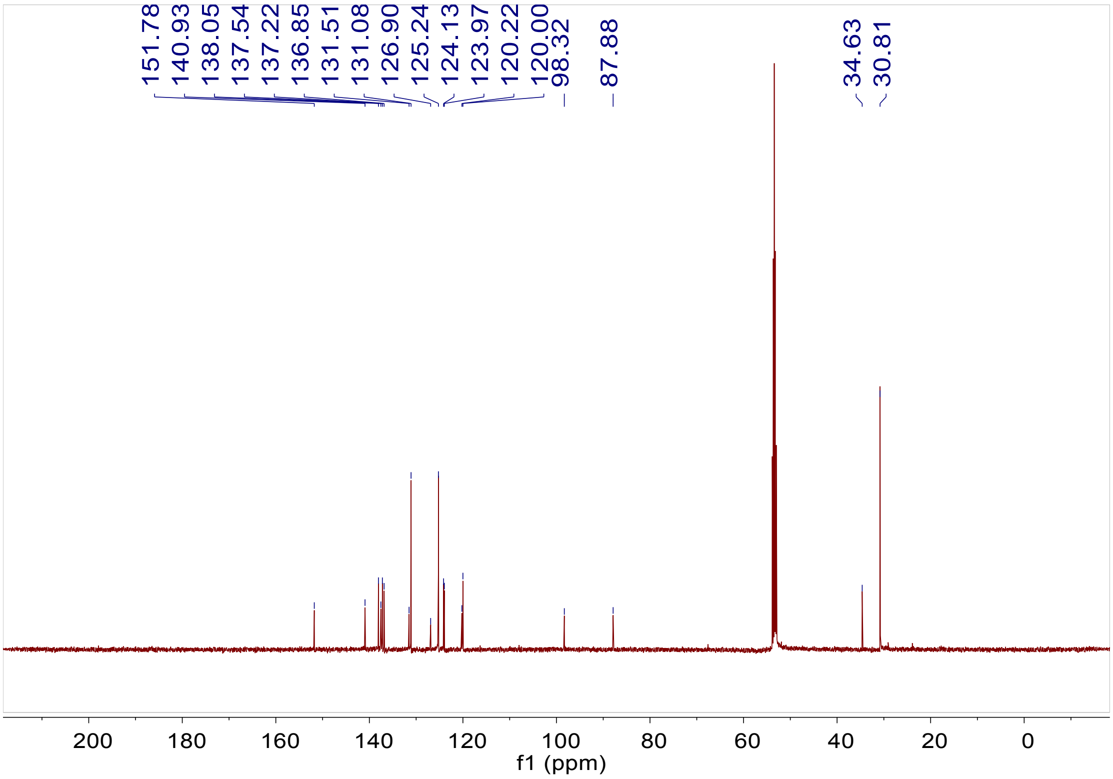

Figure S27. ^{13}C NMR of compound **4-Ib** in CD_2Cl_2 at room temperature.

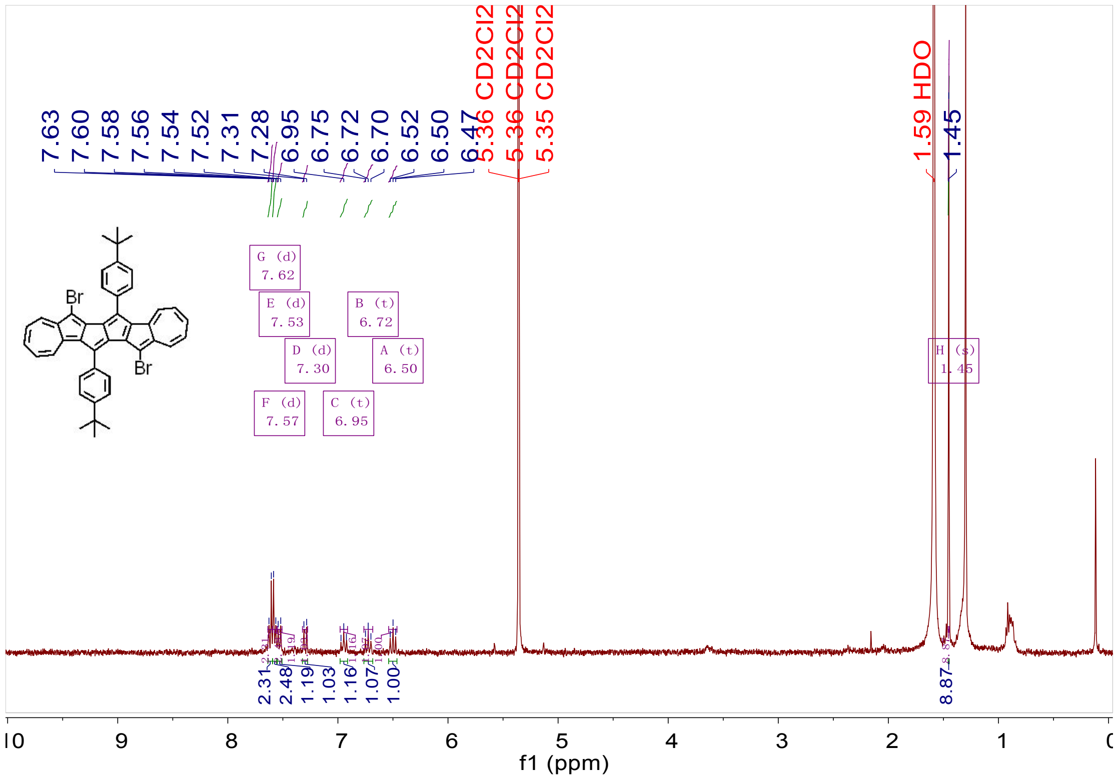

Figure S28. ^{1}H NMR of compound **1-I** in CD_2Cl_2 at room temperature.

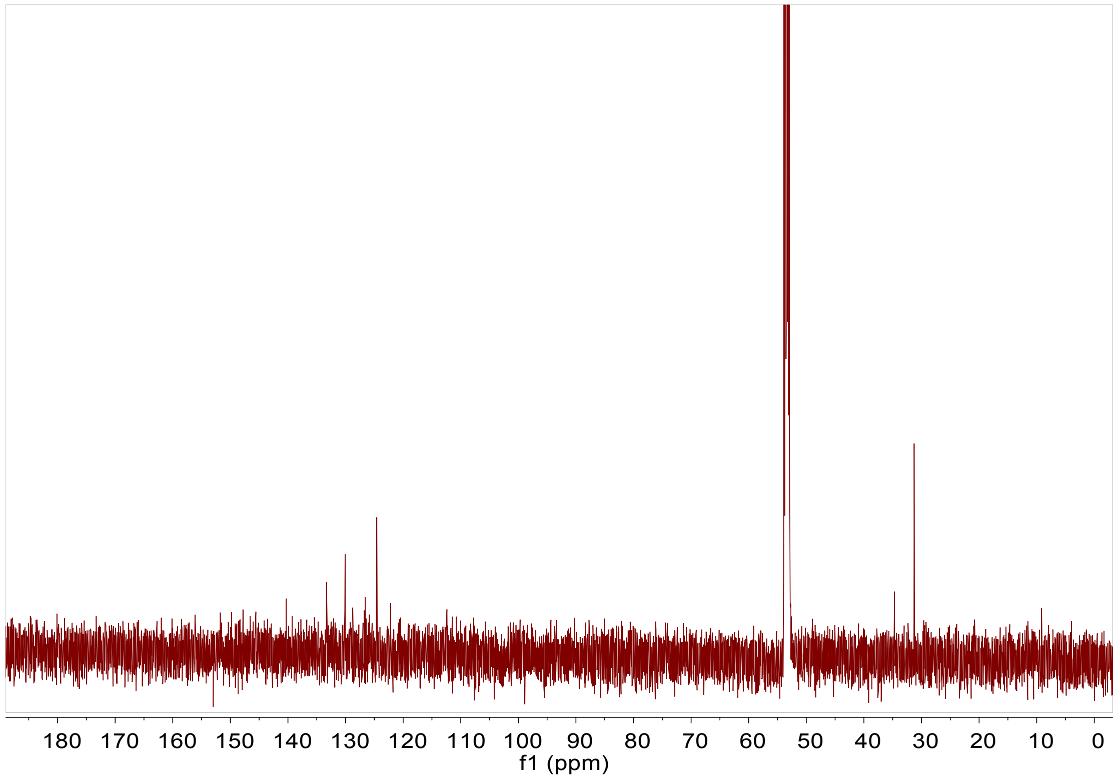

Figure S29. ^{13}C NMR of compound **1-I** in CD_2Cl_2 at room temperature.

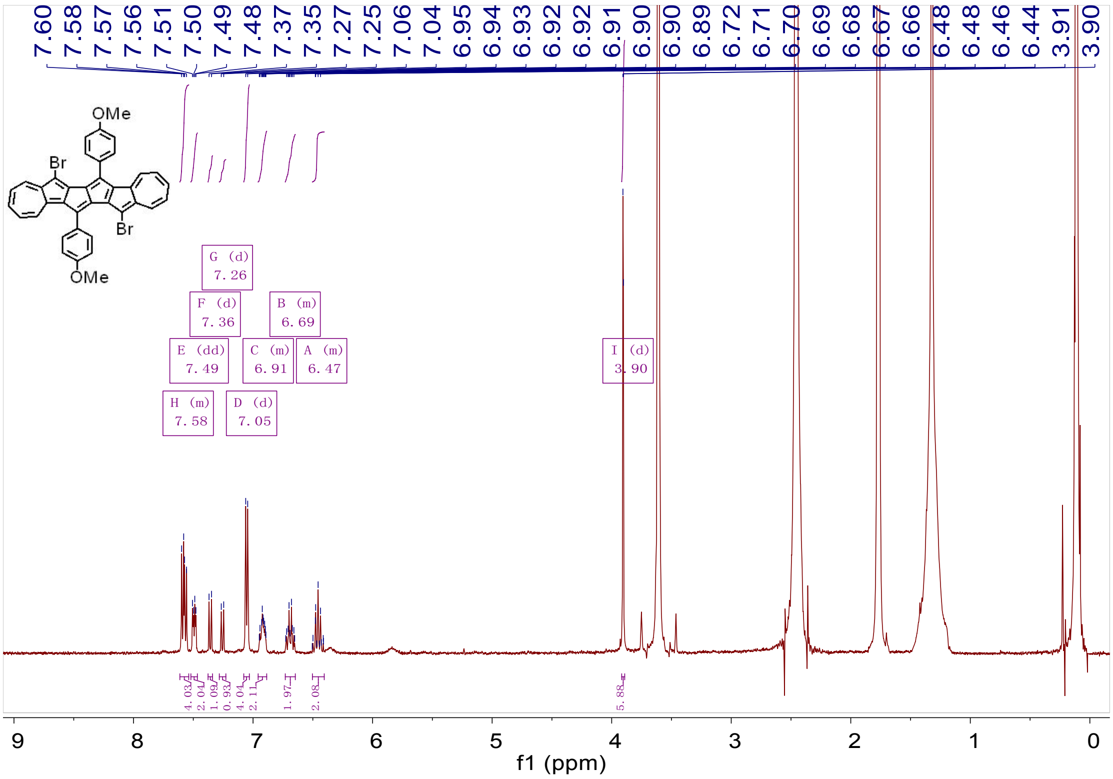

Figure S30. ^{1}H NMR of compound **1-II** in THF-d_8 at room temperature.

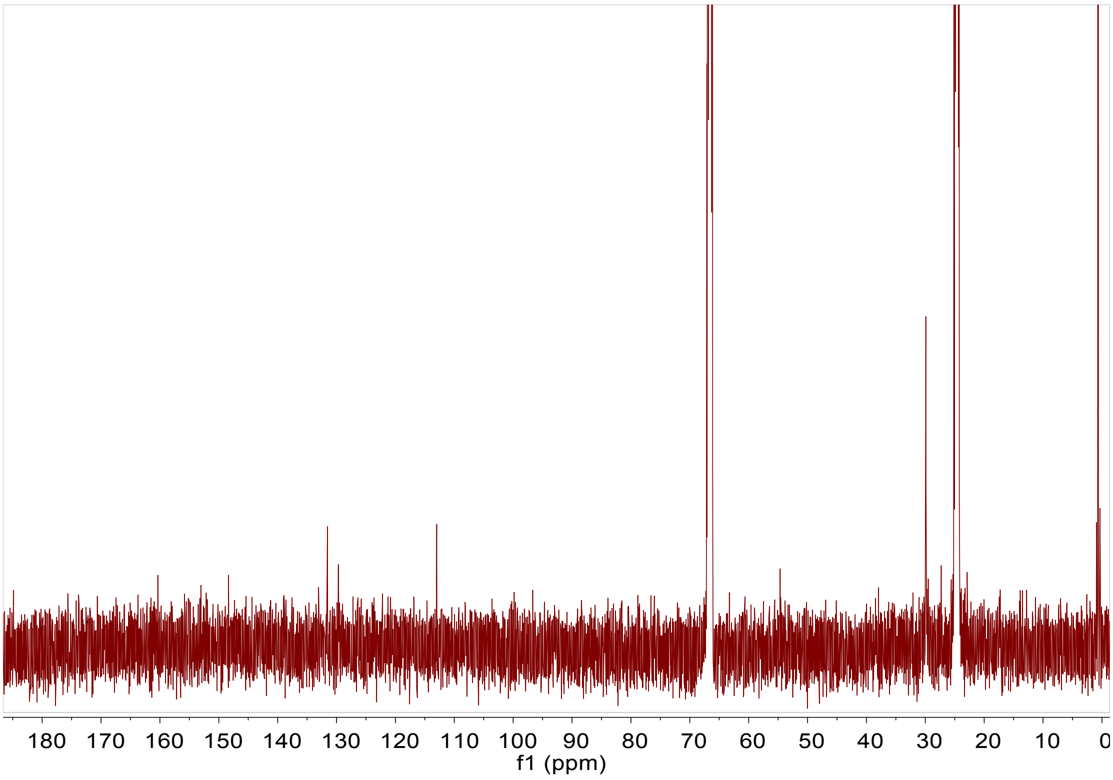

Figure S31. ^{13}C NMR of compound **1-II** in THF-d_8 at room temperature.

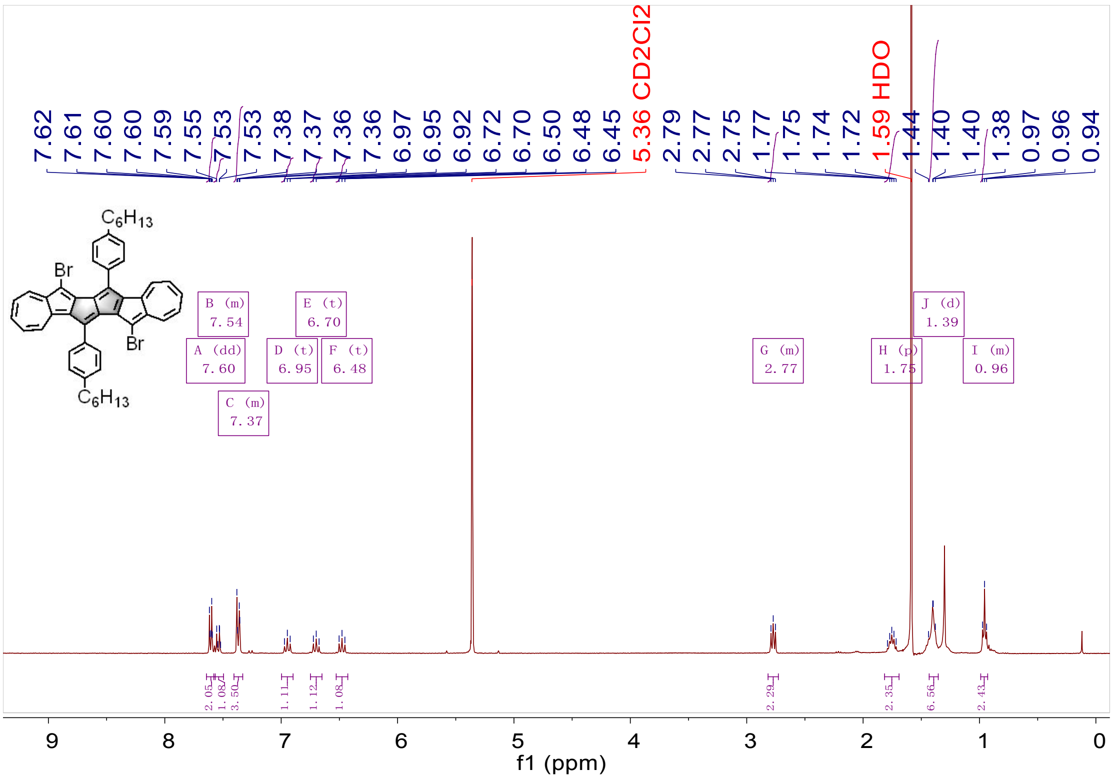

Figure S32. ^{1}H NMR of compound **1-III** in CD_2Cl_2 at room temperature.

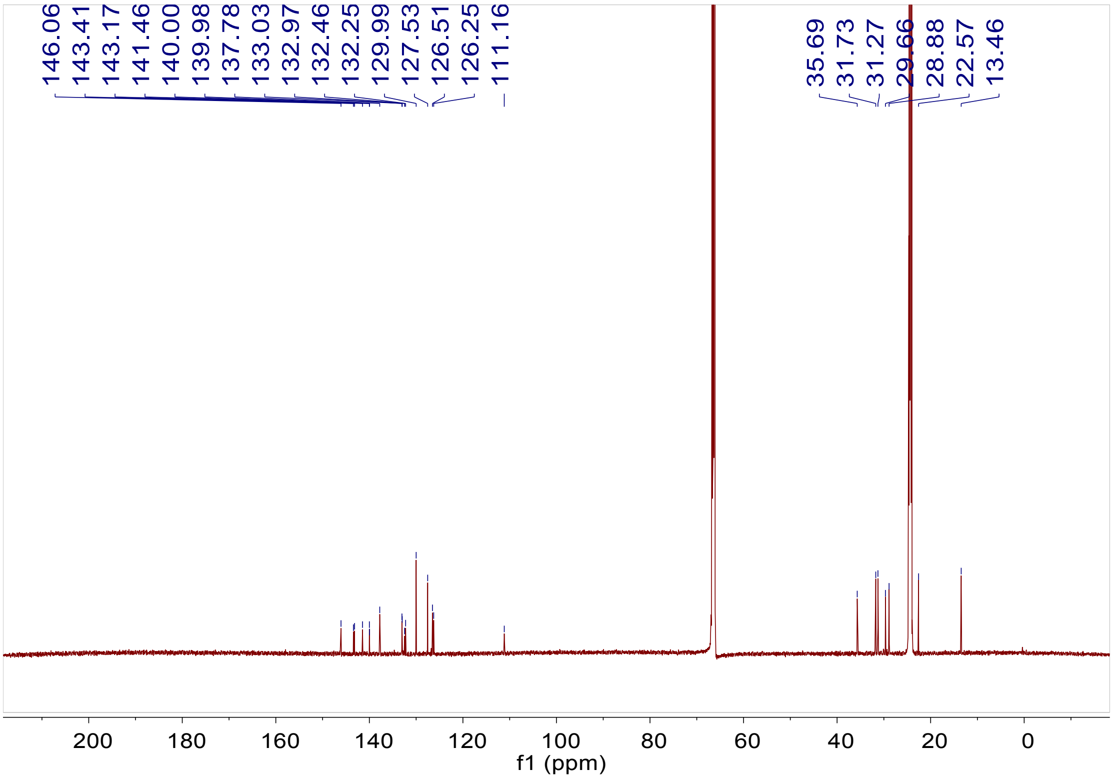

Figure S33. ^{13}C NMR of compound **1-III** in THF-d_8 at room temperature.

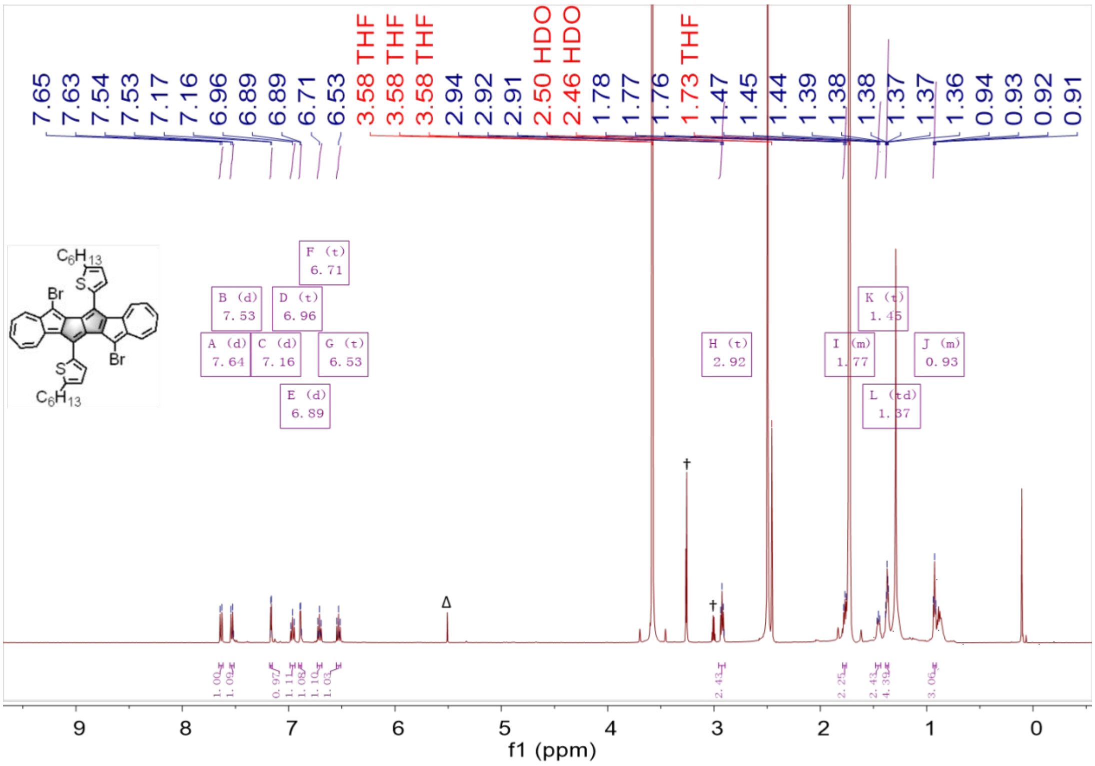

Figure S34. ^{1}H NMR of compound **1-IV** in THF-d_8 at room temperature. (Solvent peak: Δ CH_2Cl_2 5.51 ppm, †CH_3OH 3.27, 3.02 ppm).

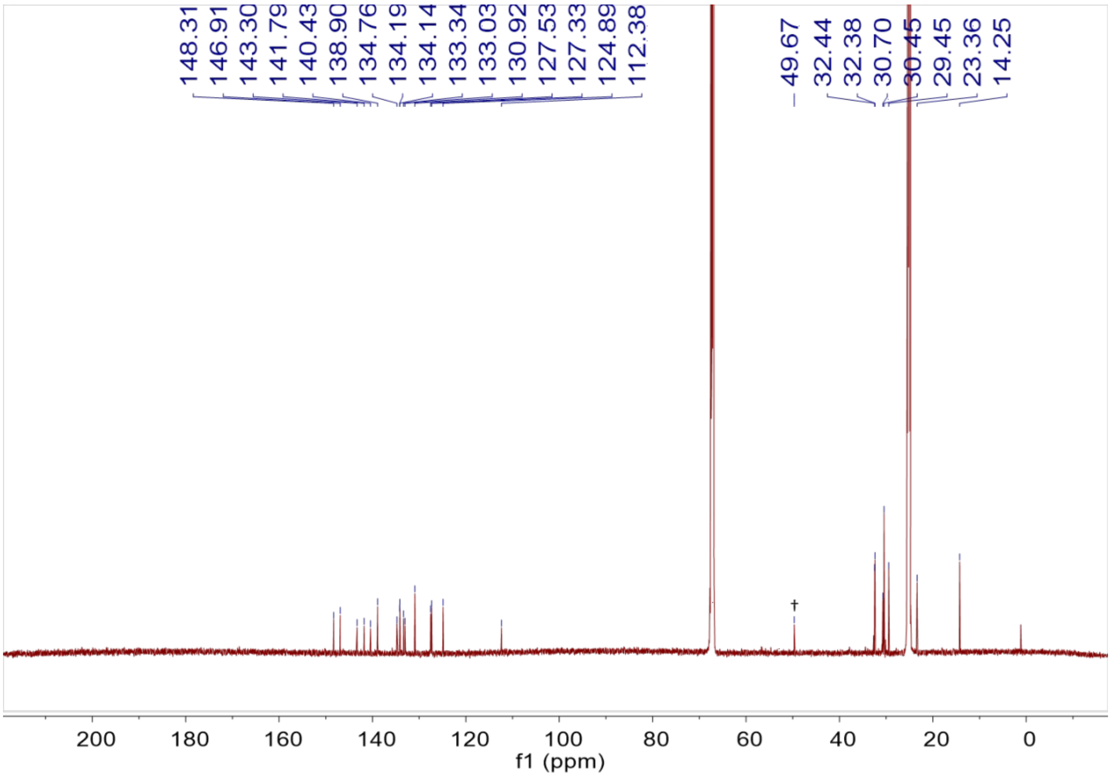

Figure S35. ^{13}C NMR of compound **1-IV** in THF-d_8 at room temperature. (Solvent peak: †CH_3OH 49.64 ppm).

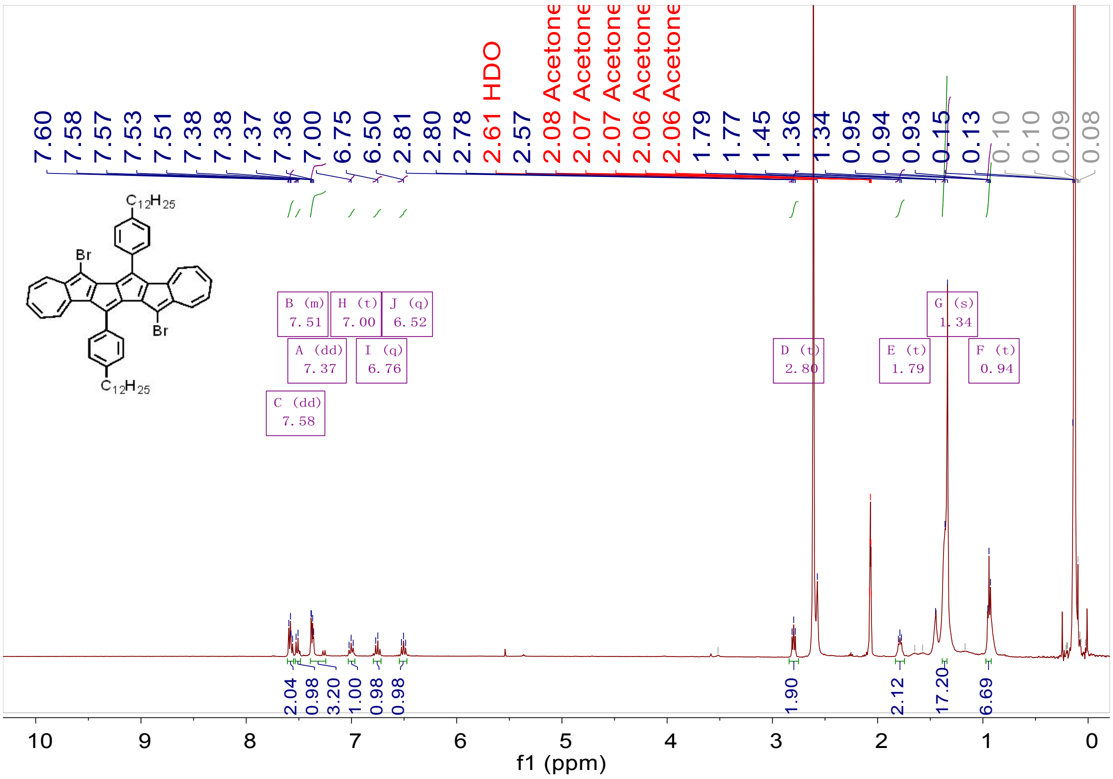

Figure S36. ^{1}H NMR of compound **1-V** in Acetone-d_6/CS_2 (1/1) at room temperature.

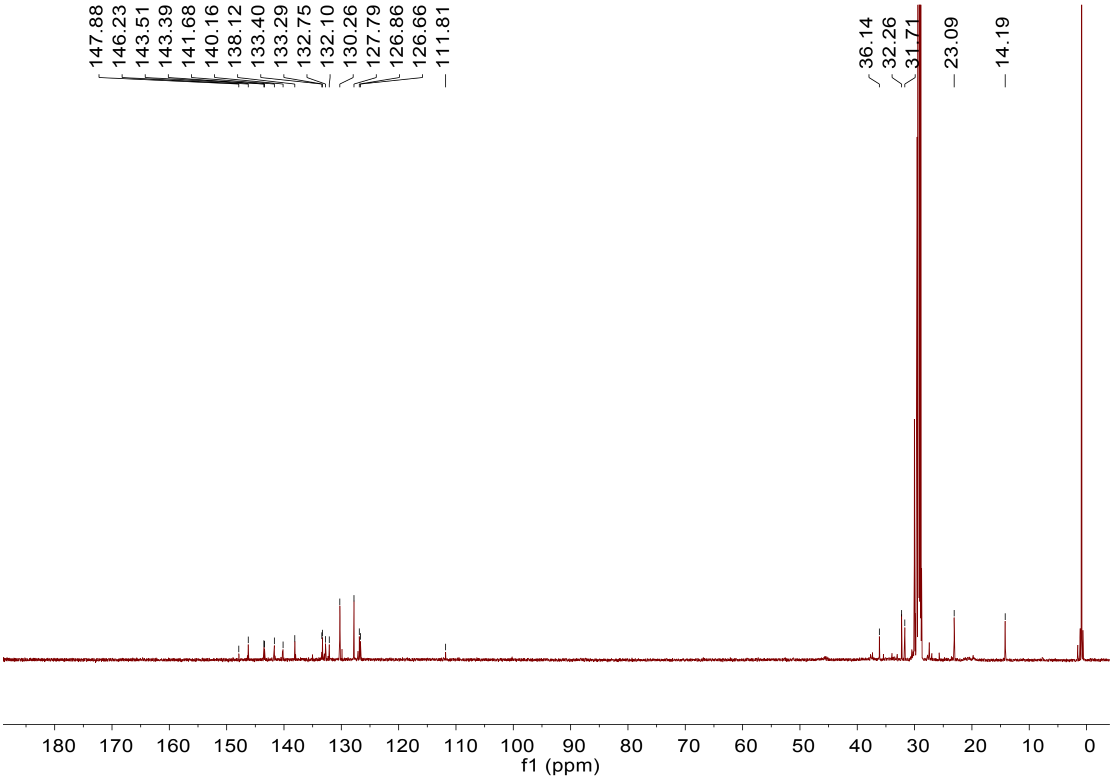

Figure S37. ^{13}C NMR of compound **1-V** in Acetone-d_6/CS_2 (1/1) at room temperature.

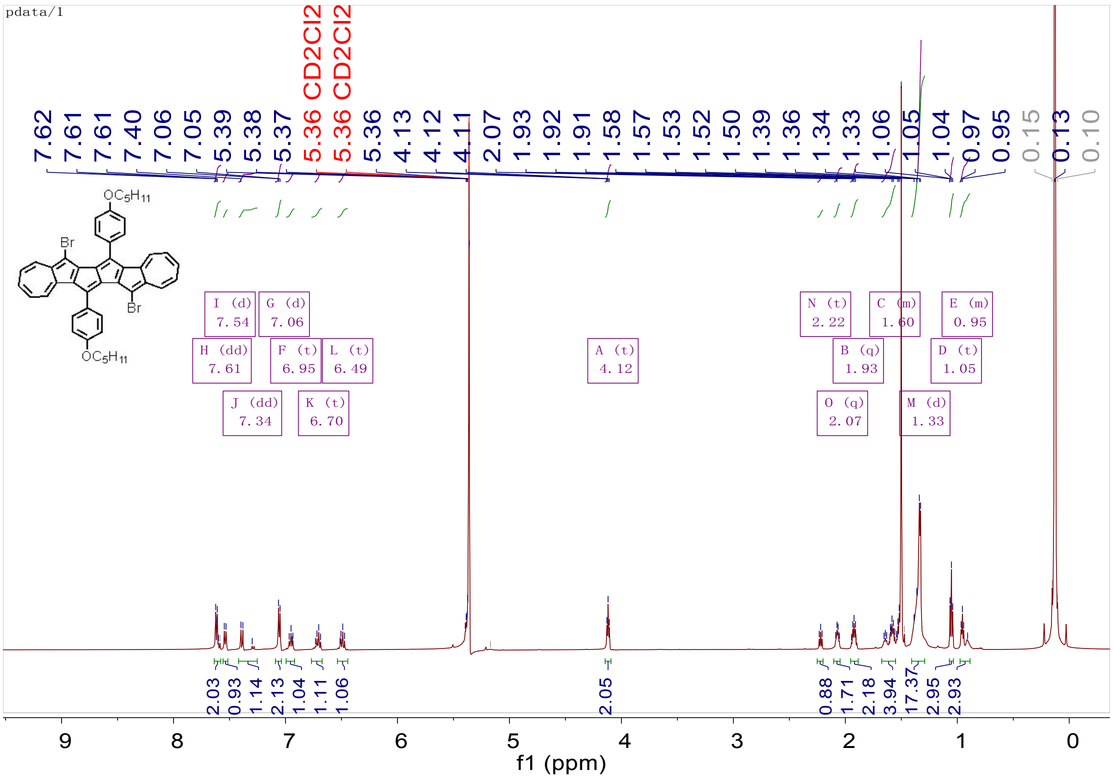

Figure S38. ^{1}H NMR of compound **1-VI** in CD_2Cl_2 at room temperature.

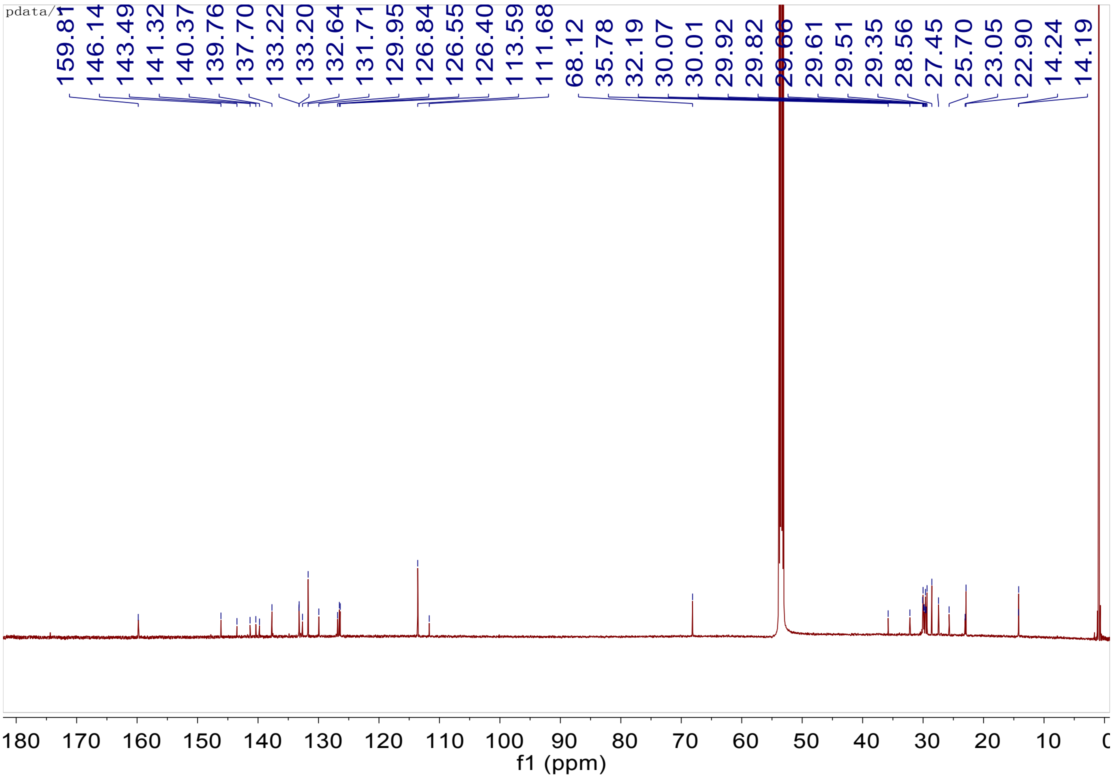

Figure S39. ^{13}C NMR of compound **1-VI** in CD_2Cl_2 at room temperature.

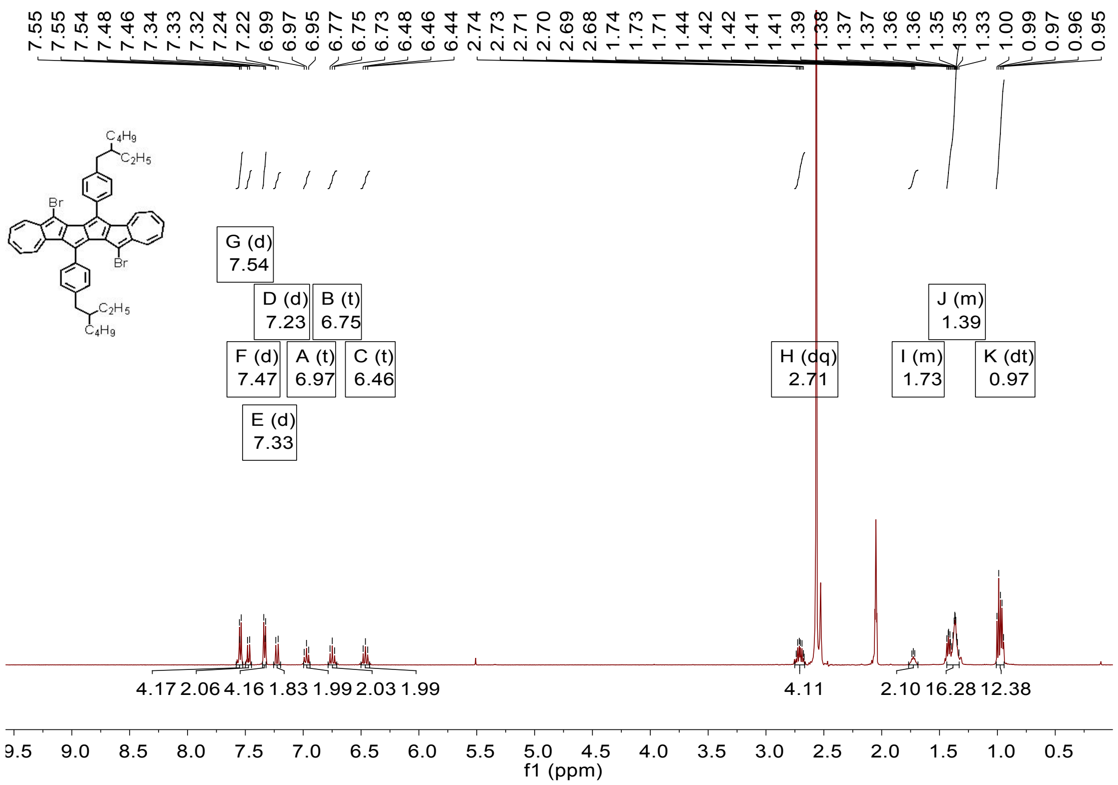

Figure S40. ^{1}H NMR of compound **1-VII** in Acetone-d_6/CS_2 (1/1) at room temperature.

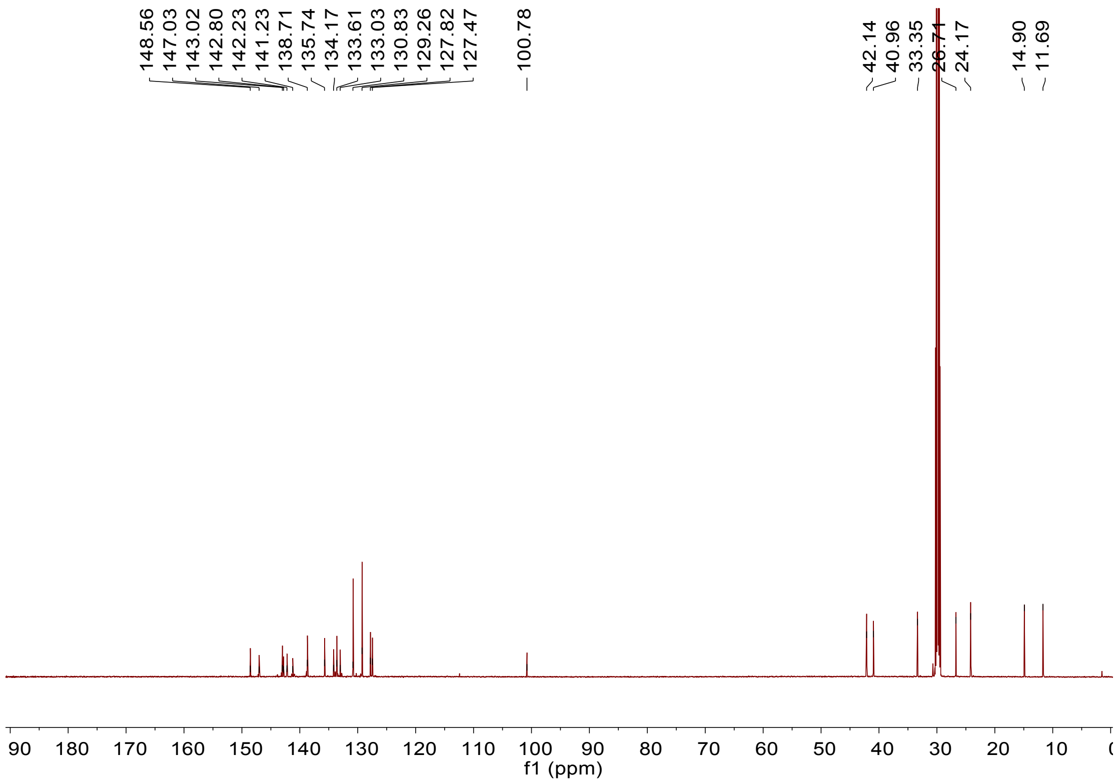

Figure S41. ^{13}C NMR of compound **1-VII** in Acetone-d_6/CS_2 (1/1) at room temperature.

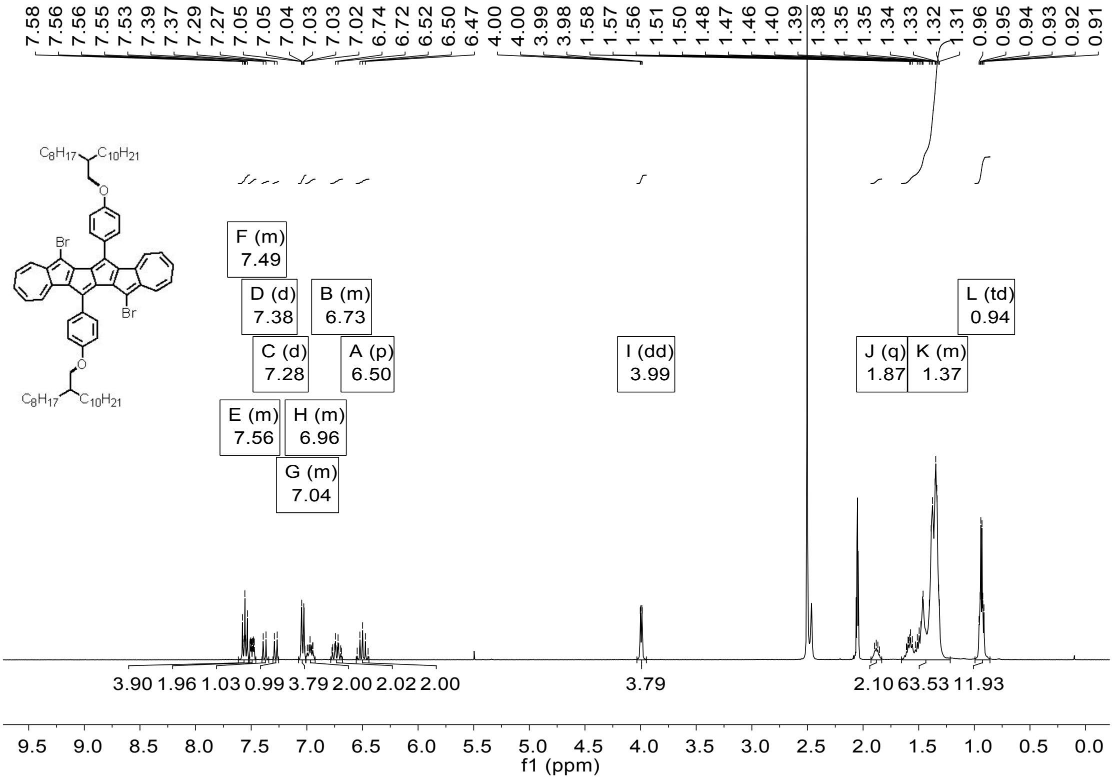

Figure S42. ^{1}H NMR of compound **1-VIII** in Acetone-d_6/CS_2 (1/1) at room temperature.

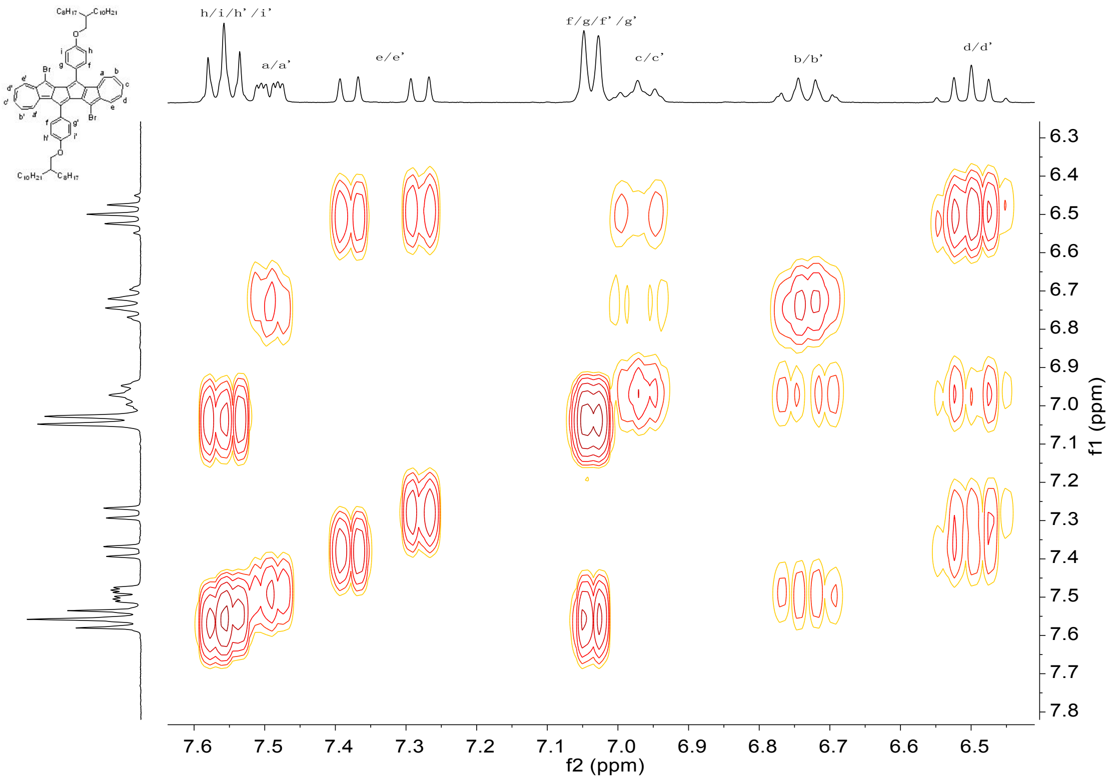

Figure S43. ^{1}H NMR (2D-COSY) of compound **1-VIII** in Acetone-d_6/CS_2 (1/1) at room temperature.

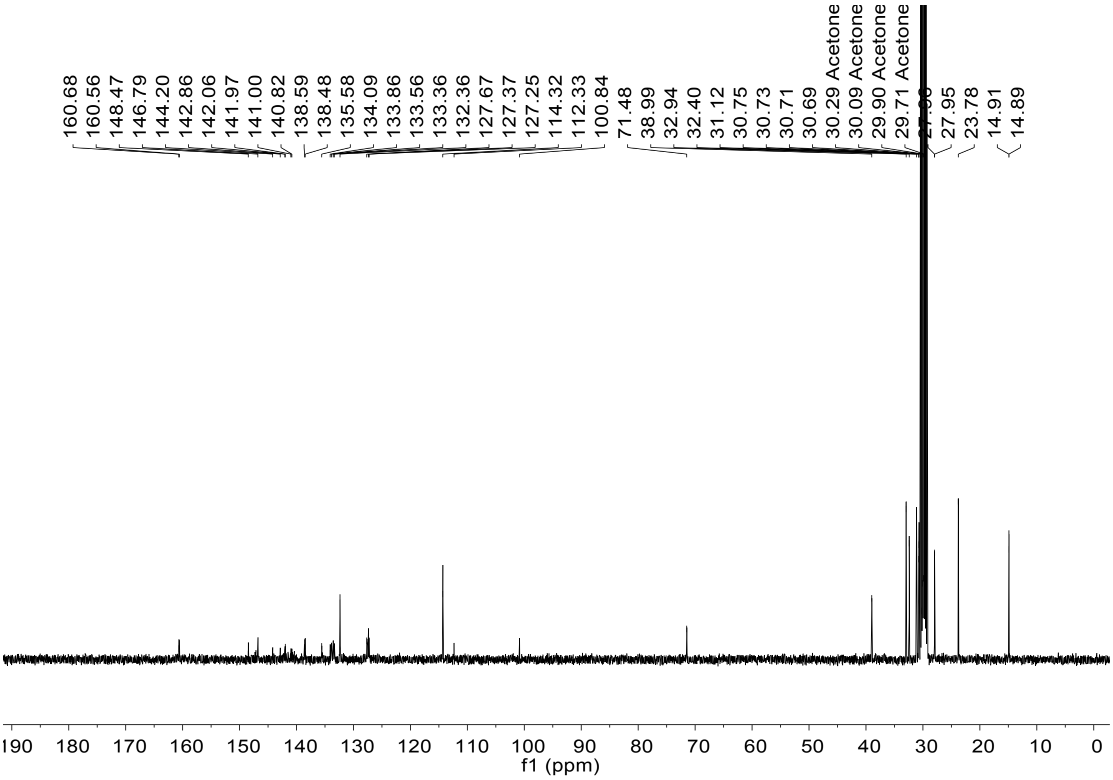

Figure S44. ^{13}C NMR of compound **1-VIII** in Acetone-d_6/CS_2 (1/1) at room temperature.

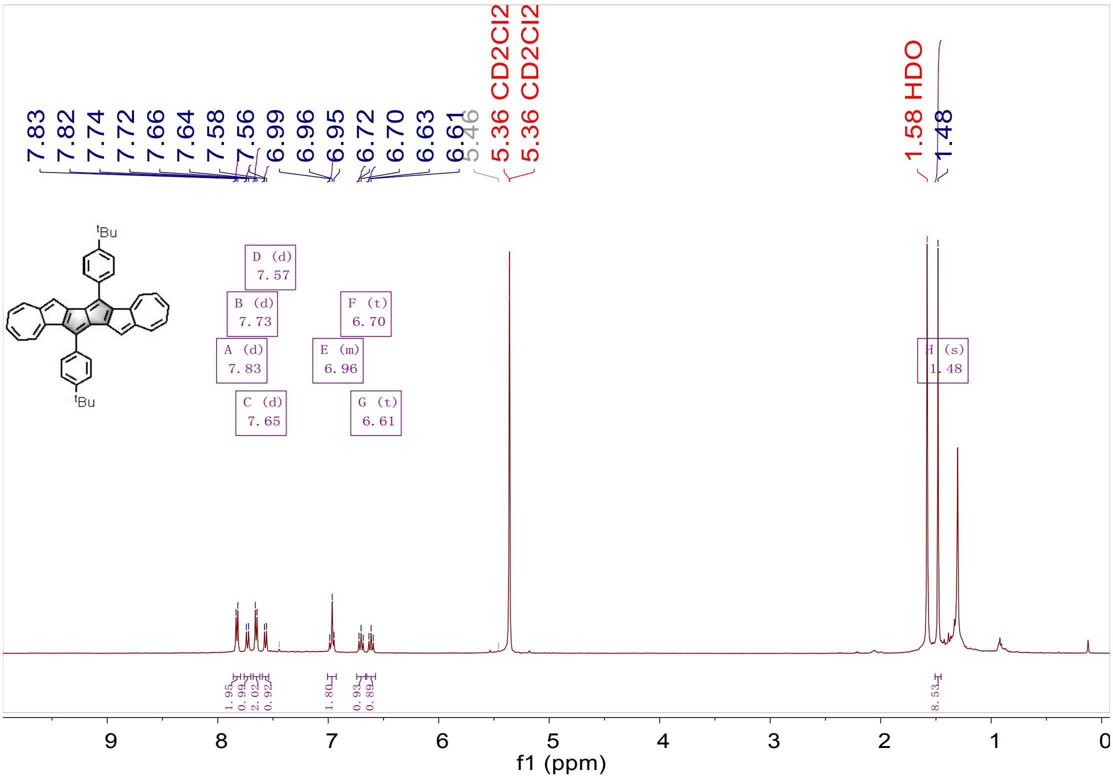

Figure S45. ^{1}H NMR of compound **1-Ib** in CD_2Cl_2 at room temperature.

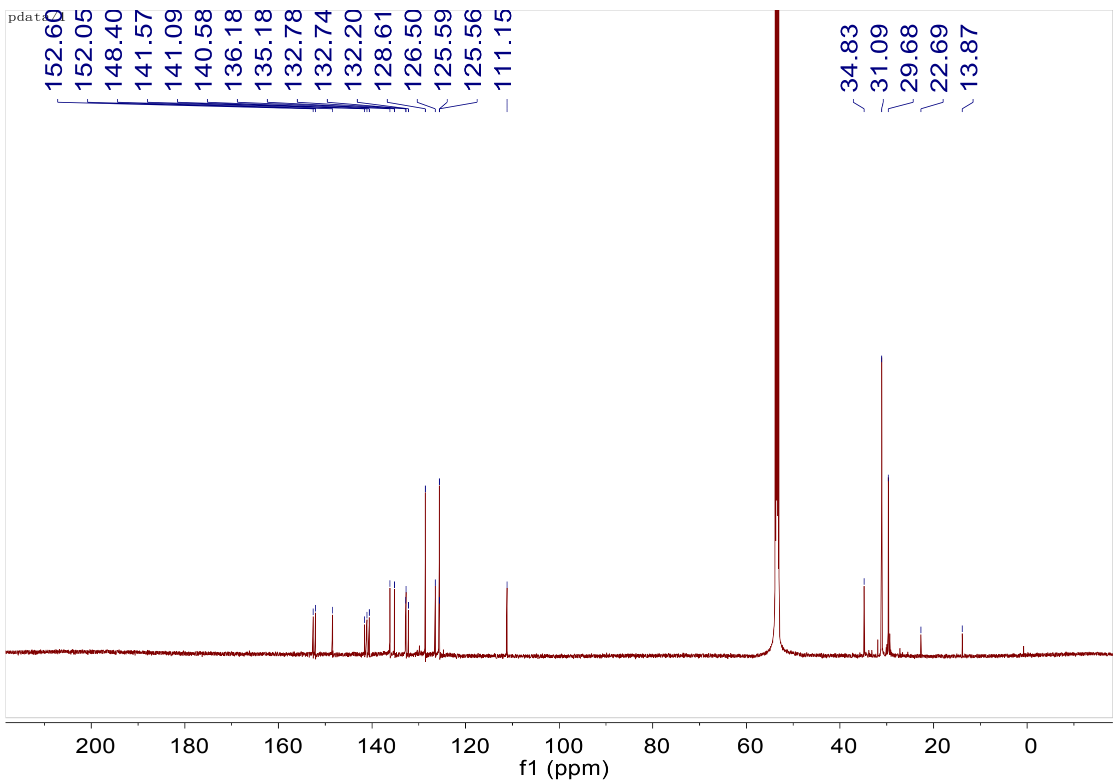

Figure S46. ^{13}C NMR of compound **1-Ib** in CD_2Cl_2 at room temperature.

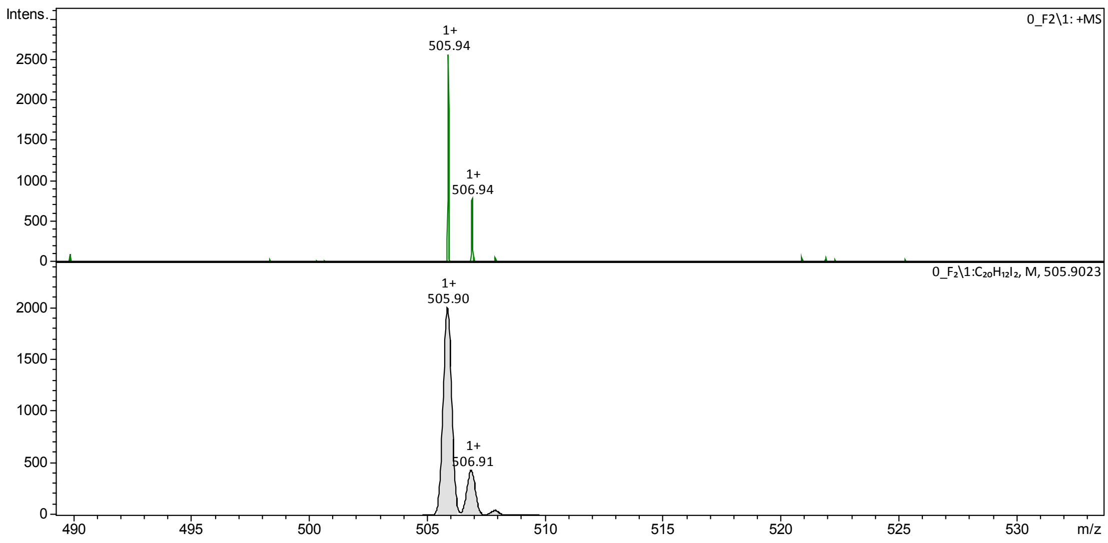

Figure S47. MS spectrum of compound **2** (up: experimental; down: simulated).

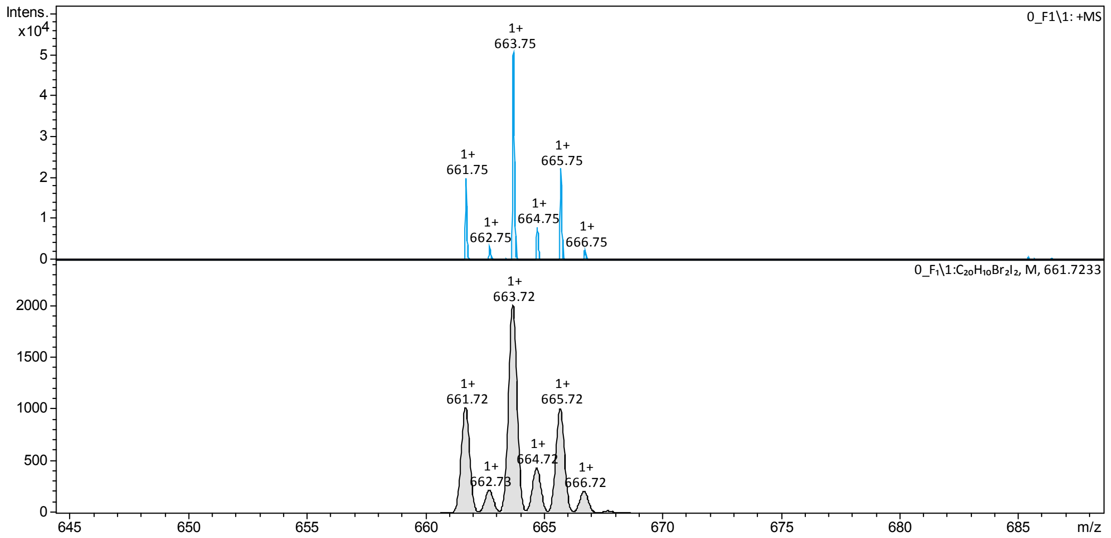

Figure S48. MS spectrum of compound **3** (up: experimental; down: simulated).

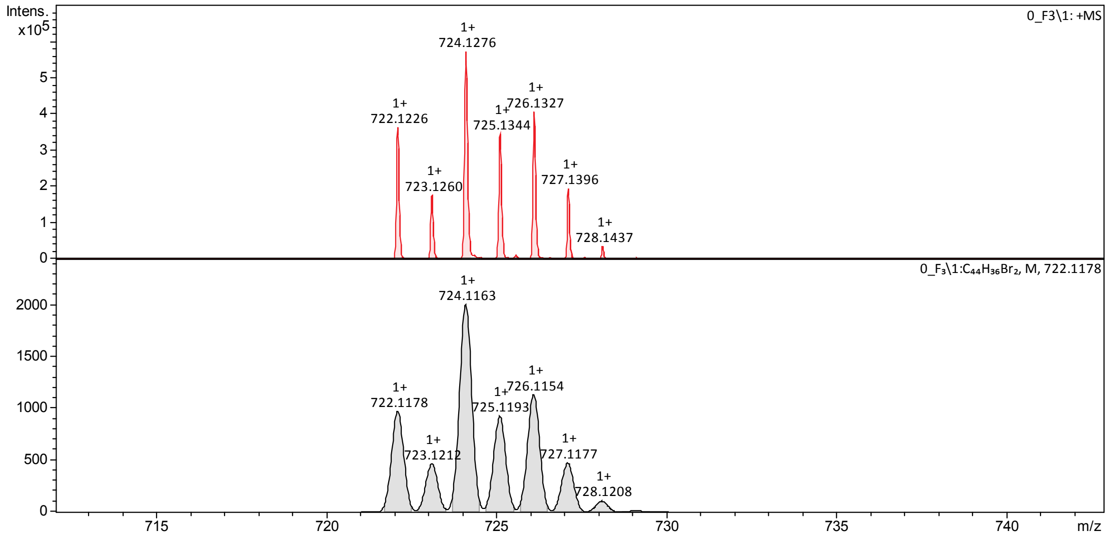

Figure S49. HR-MS spectrum of compound **4-I** (up: experimental; down: simulated).

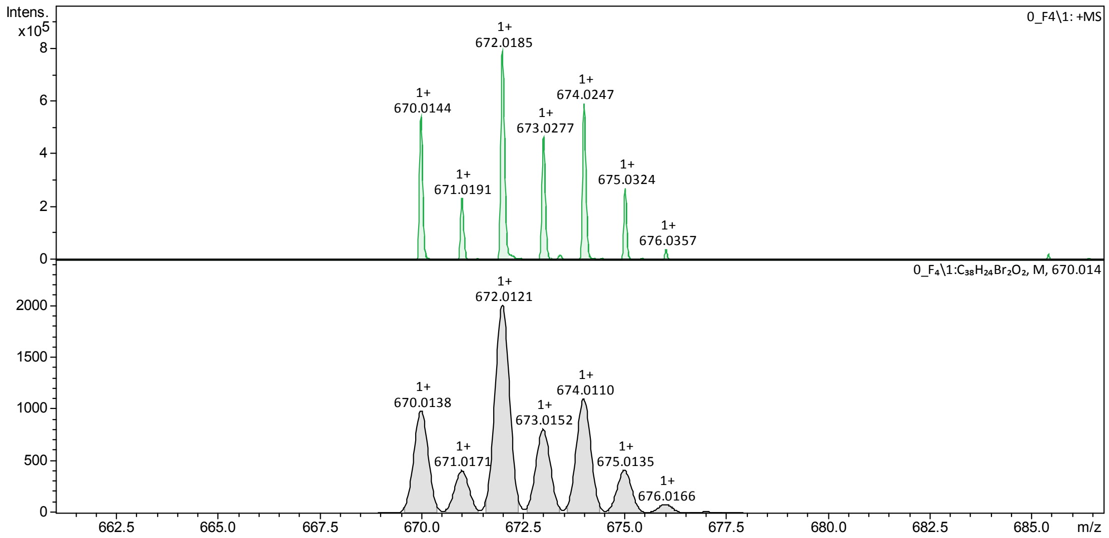

Figure S50. HR-MS spectrum of compound **4-II** (up: experimental; down: simulated).

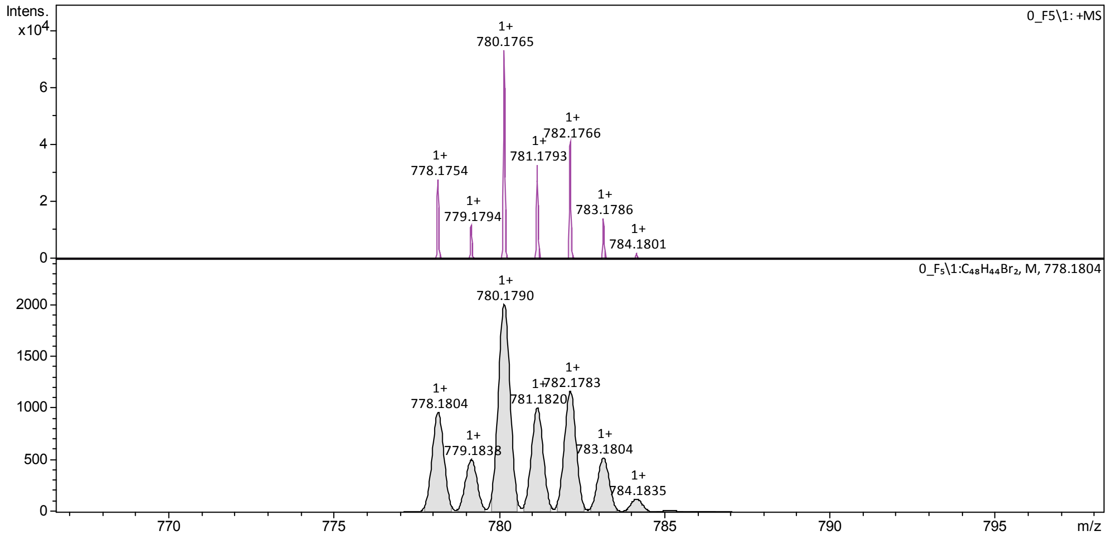

Figure S51. HR-MS spectrum of compound **4-III** (up: experimental; down: simulated).

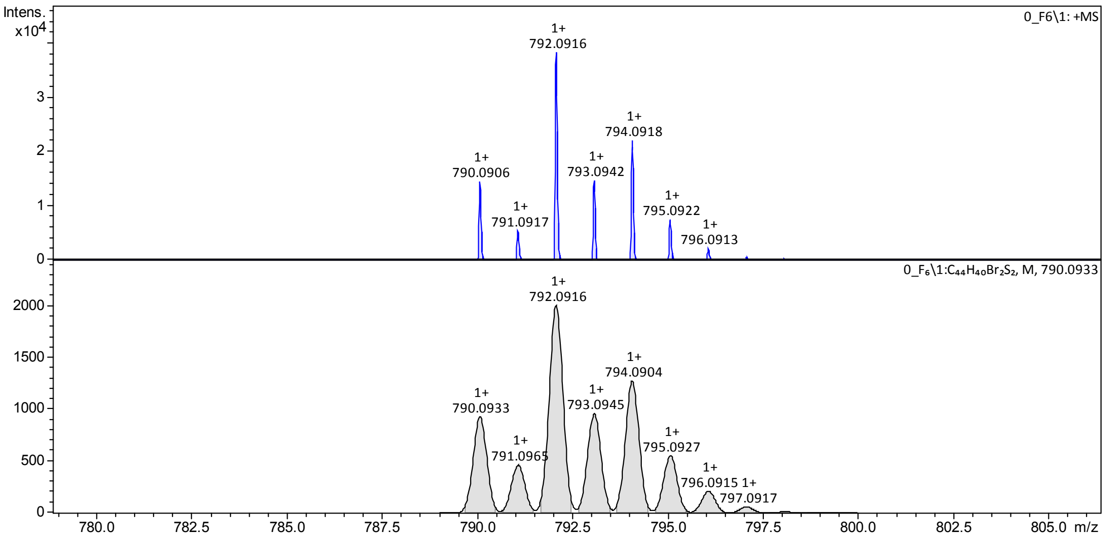

Figure S52. HR-MS spectrum of compound **4-IV** (up: experimental; down: simulated).

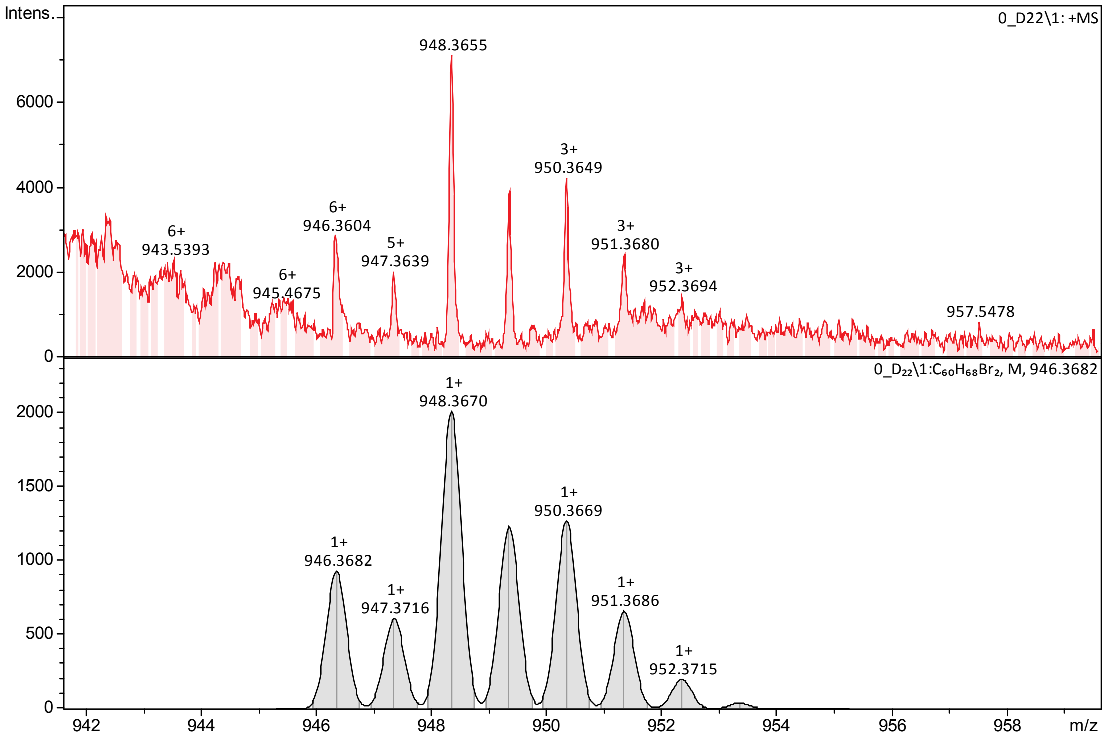

Figure S53. HR-MS spectrum of compound **4-V** (up: experimental; down: simulated).

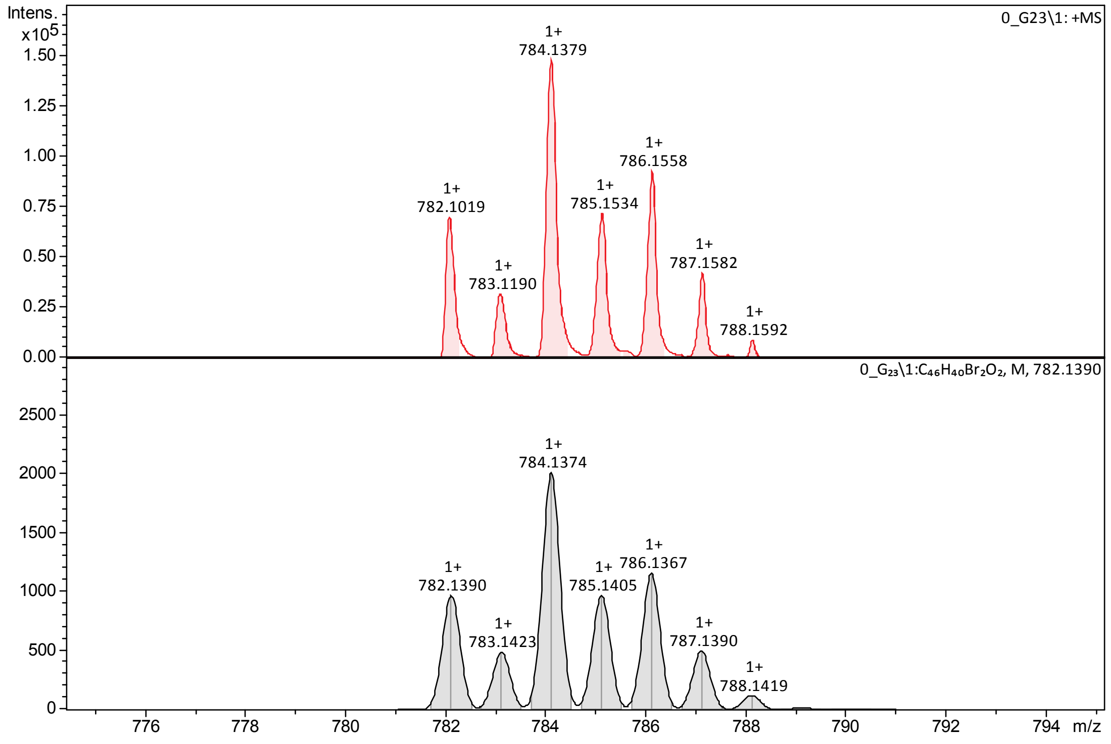

Figure S54. HR-MS spectrum of compound **4-VI** (up: experimental; down: simulated).

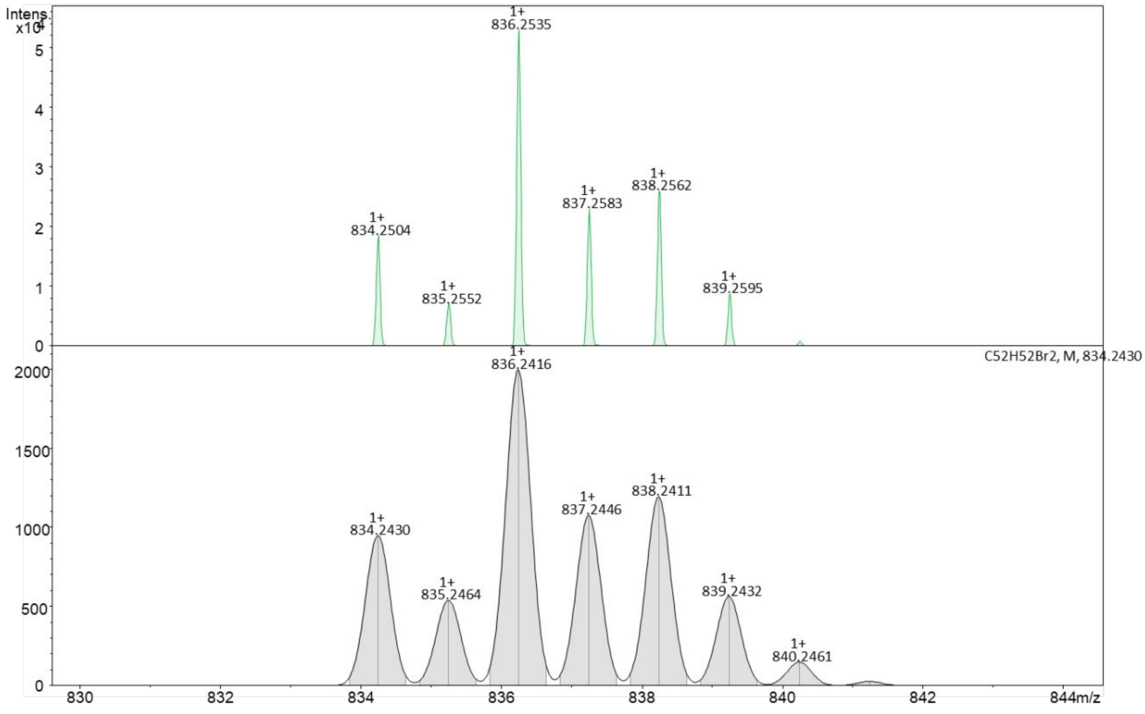

Figure S55. HR-MS spectrum of compound **4-VII** (up: experimental; down: simulated).

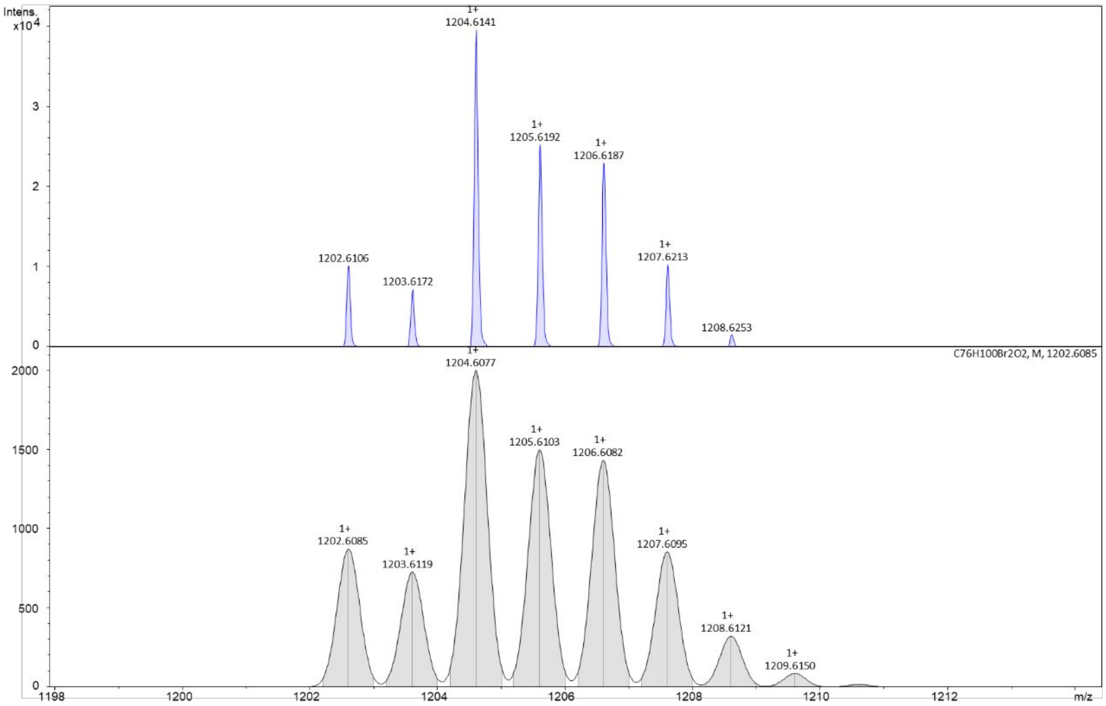

Figure S56. HR-MS spectrum of compound **4-VIII** (up: experimental; down: simulated).

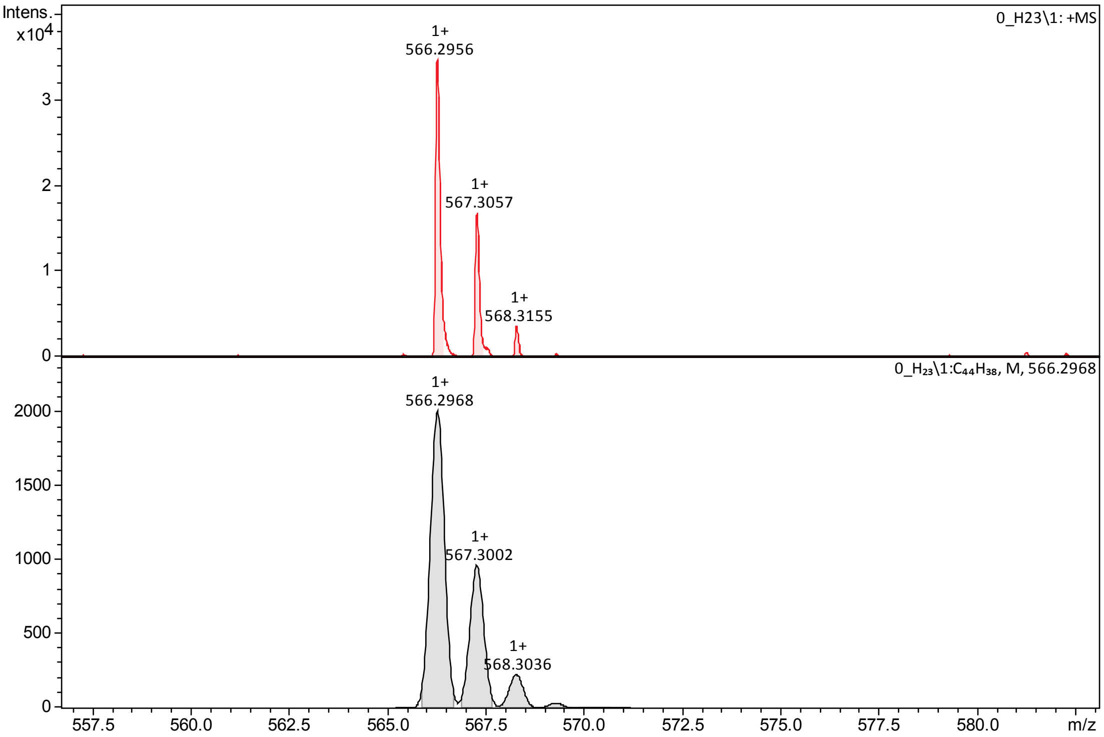

Figure S57. HR-MS spectrum of compound **4-Ib** (up: experimental; down: simulated).

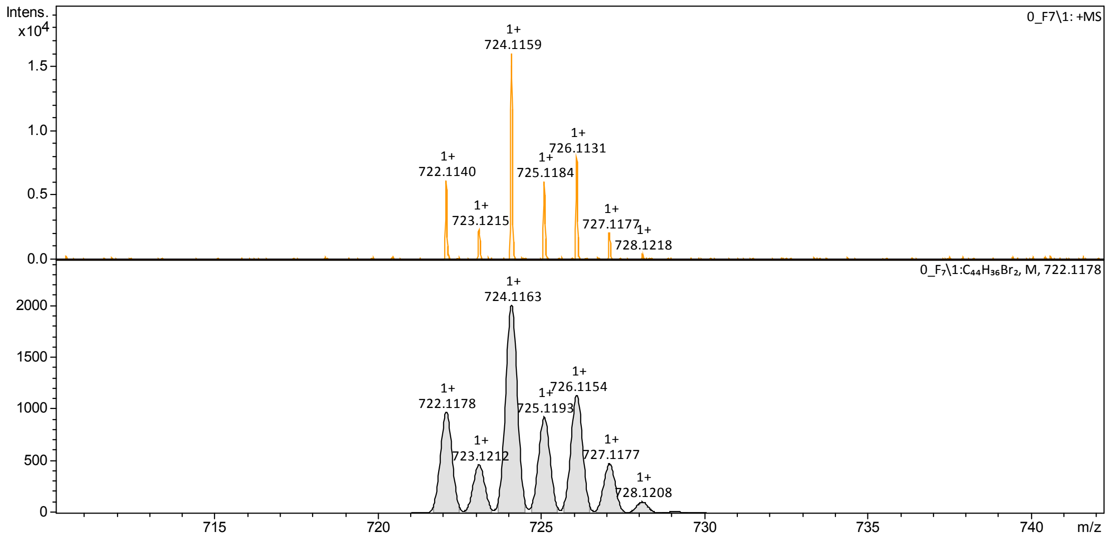

Figure S58. HR-MS spectrum of compound **1-I** (up: experimental; down: simulated).

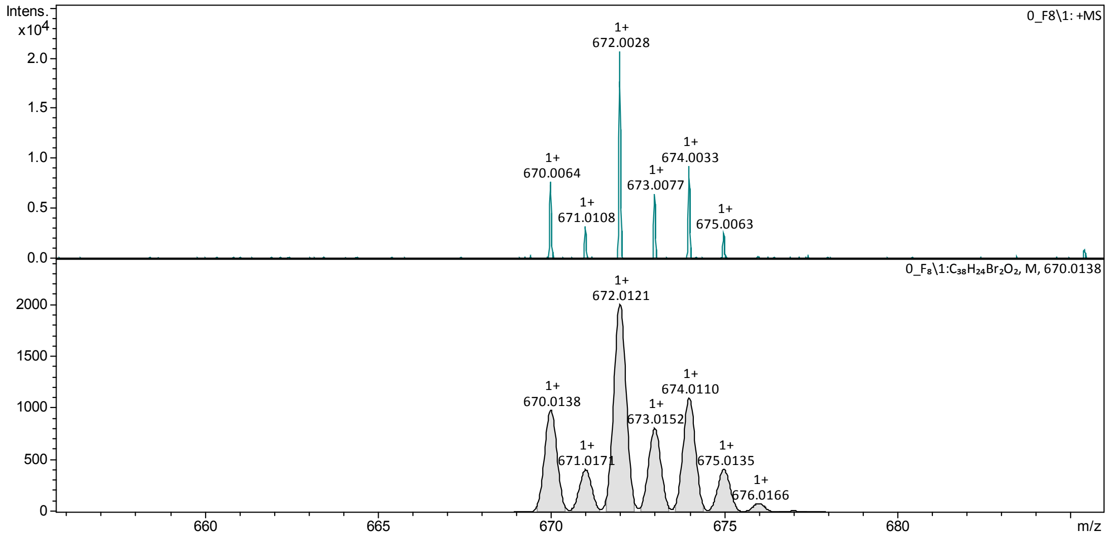

Figure S59. HR-MS spectrum of compound **1-II** (up: experimental; down: simulated).

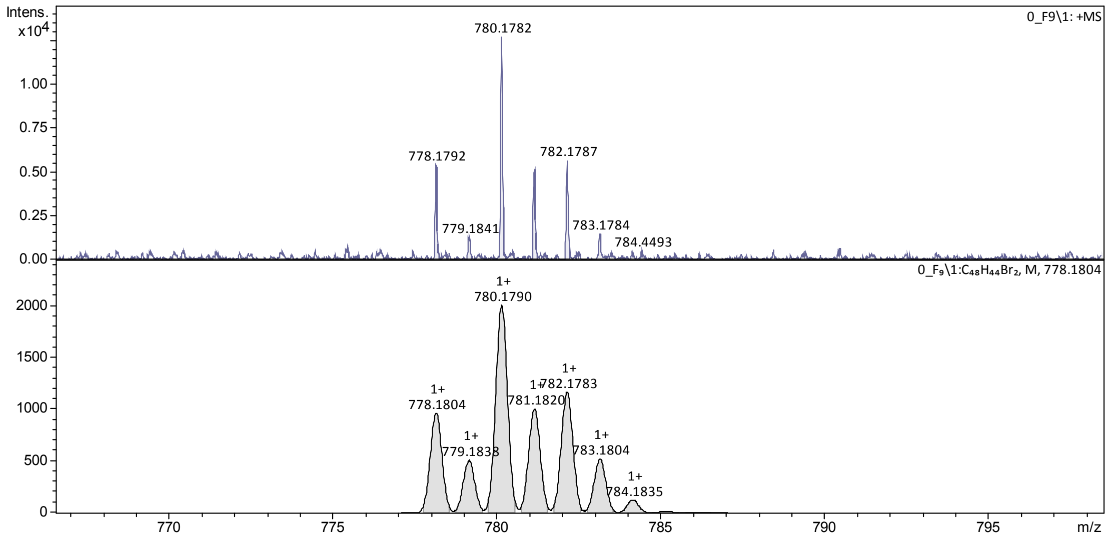

Figure S60. HR-MS spectrum of compound **1-III** (up: experimental; down: simulated).

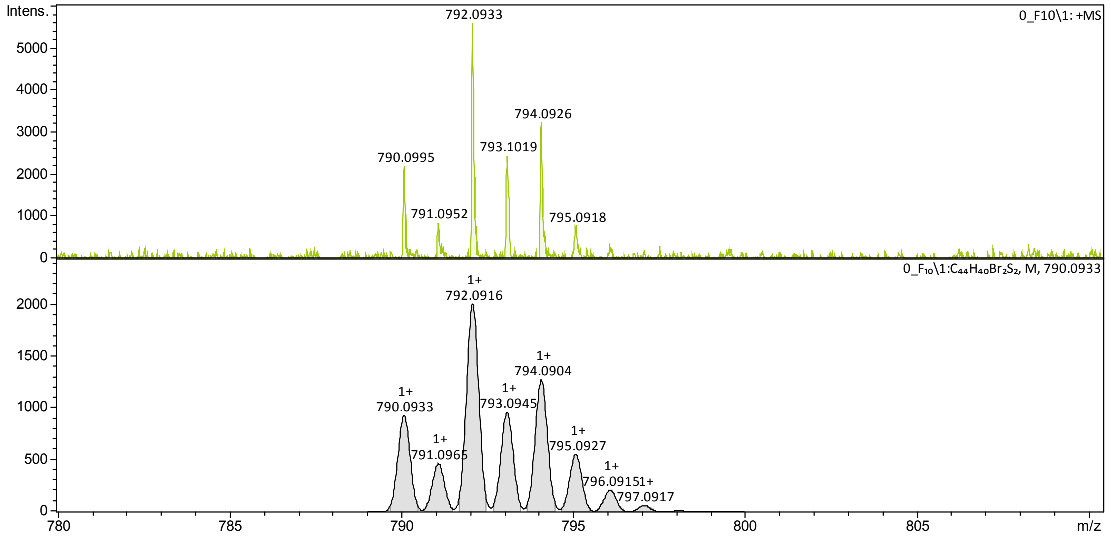

Figure S61. HR-MS spectrum of compound **1-IV** (up: experimental; down: simulated).

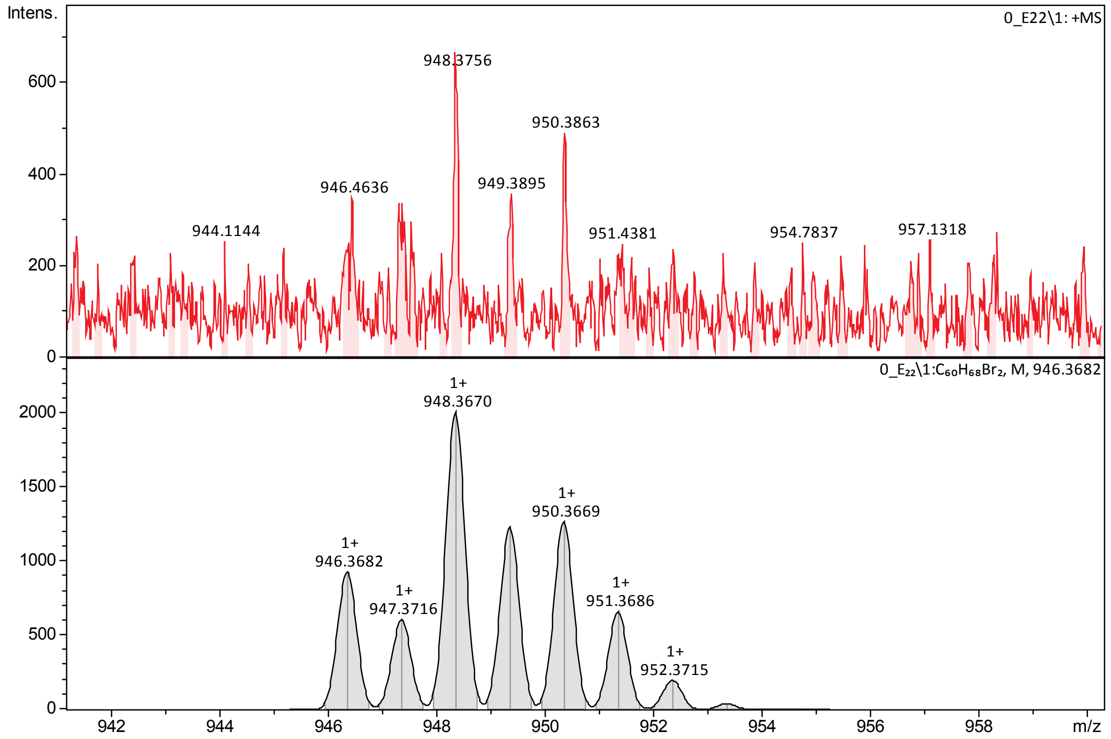

Figure S62. HR-MS spectrum of compound **1-V** (up: experimental; down: simulated).

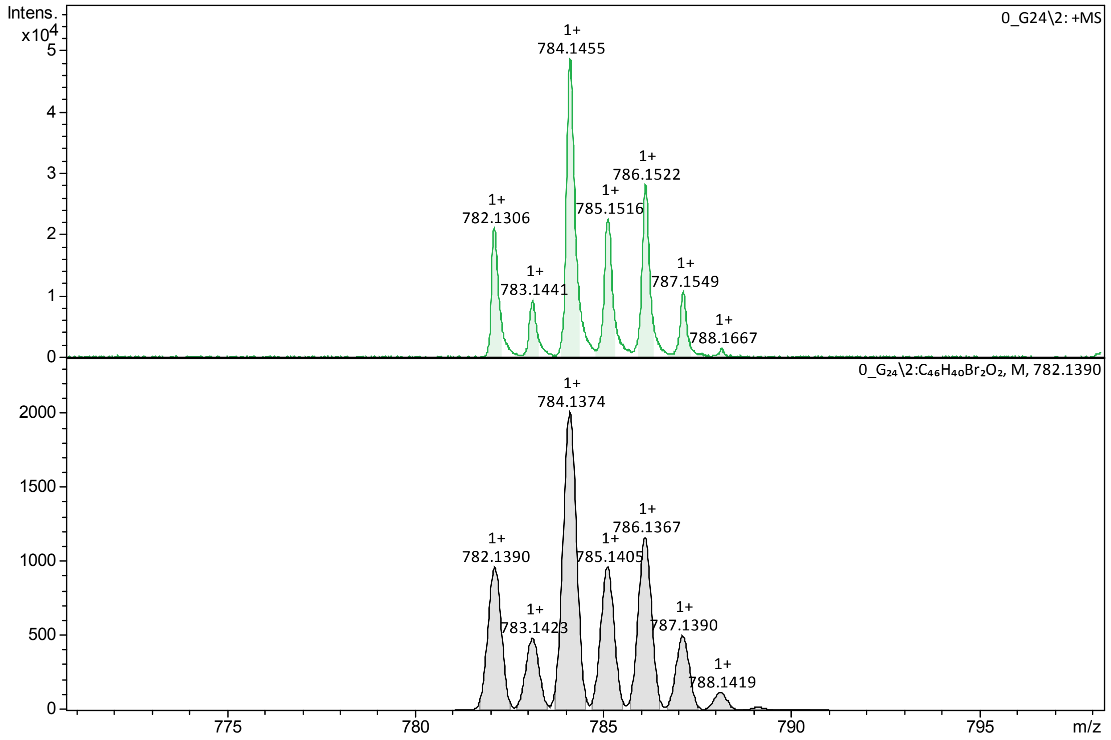

Figure S63. HR-MS spectrum of compound **1-VI** (up: experimental; down: simulated).

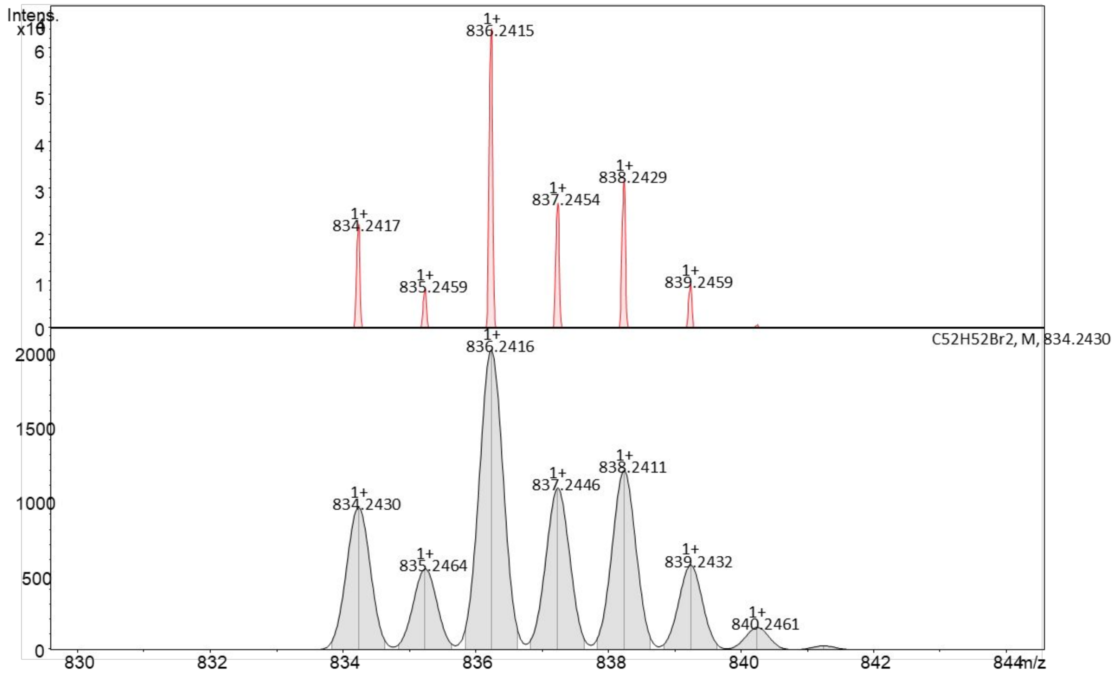

Figure S64. HR-MS spectrum of compound **1-VII** (up: experimental; down: simulated).

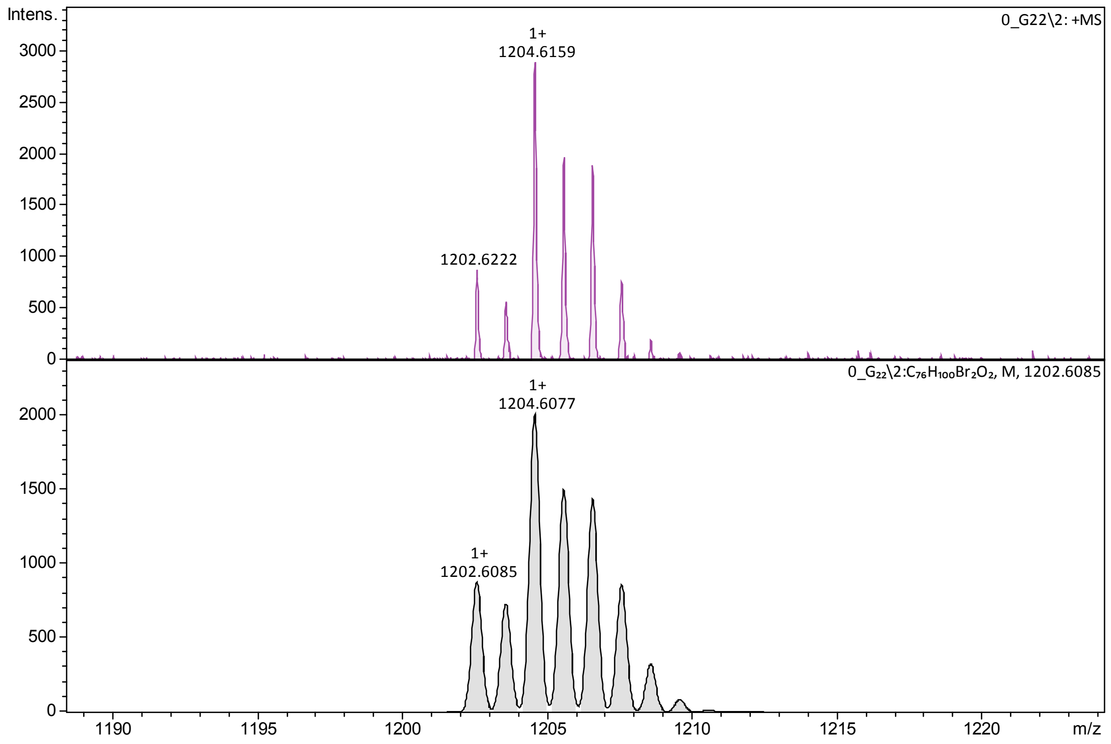

Figure S65. HR-MS spectrum of compound **1-VIII** (up: experimental; down: simulated).

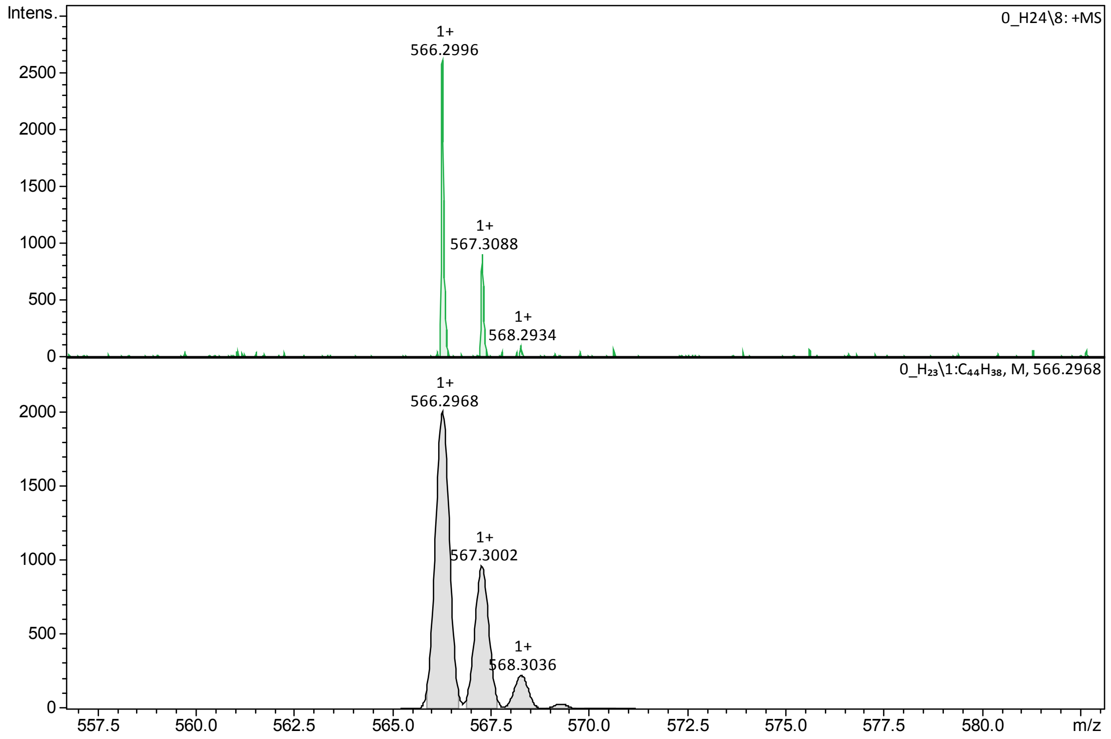

Figure S66. HR-MS spectrum of compound **1-Ib** (up: experimental; down: simulated).

6. Theoretical calculation

All density functional theory (DFT) calculation was performed using the Gaussian 16 program[4] The B3LYP functional was used for geometry optimization in the ground state. The 6-311G(d,p) basis set was used. All geometry optimization was done in the gas phase and based on the single crystal structures. Anisotropy of the induced current density (ACID) plots were calculated by Herges's method.[5] Nucleus independent chemical shifts (NICS) values were calculated using the standard gauge invariant atomic orbital (GIAO)[6] method at B3LYP functional. The 6-311+G(d,2p) basis set was used for the C, Br and H atoms. All NICS values were averaged by two positions (above and below the plane) of each molecule. The following part displays cartesian coordinates of optimized compounds.

For mechanical investigation, the optimizations employed the M06 hybrid functional developed by Truhlar and Zhao,[7] with solvent effects modeled using the SMD solvation model[8] (dichloromethane as solvent). We used the def2-SVP basis set (referred to as BS1) for all atoms.[9] Vibrational frequency calculations were performed at the same level of theory as the structural optimizations. We located transition structures using the Berny algorithm[10] and confirmed their connectivity to minima through intrinsic reaction coordinate (IRC) calculations.[11] To improve accuracy, we performed single-point energy calculations using the M06 functional with the larger def2-TZVP basis set (BS2) for all structures.[9] These calculations used tight convergence criteria and an ultrafine integral grid.

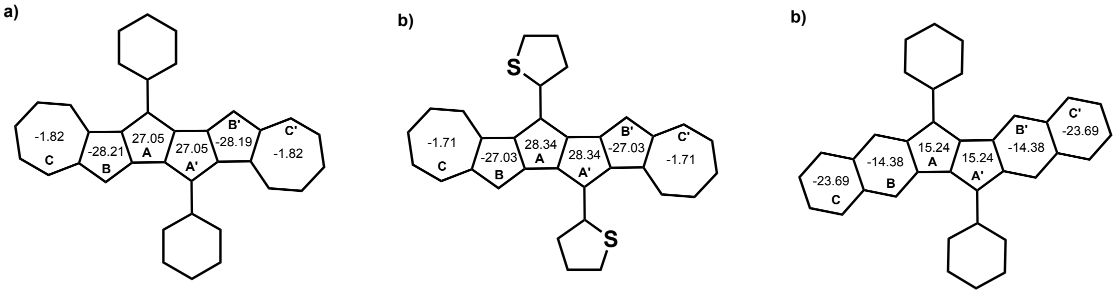

Figure S67. NICS(1)zz of **1-I** (a), **1-IV** (b) and **5** (c), alkyl chain was removed to simplify the calculation.

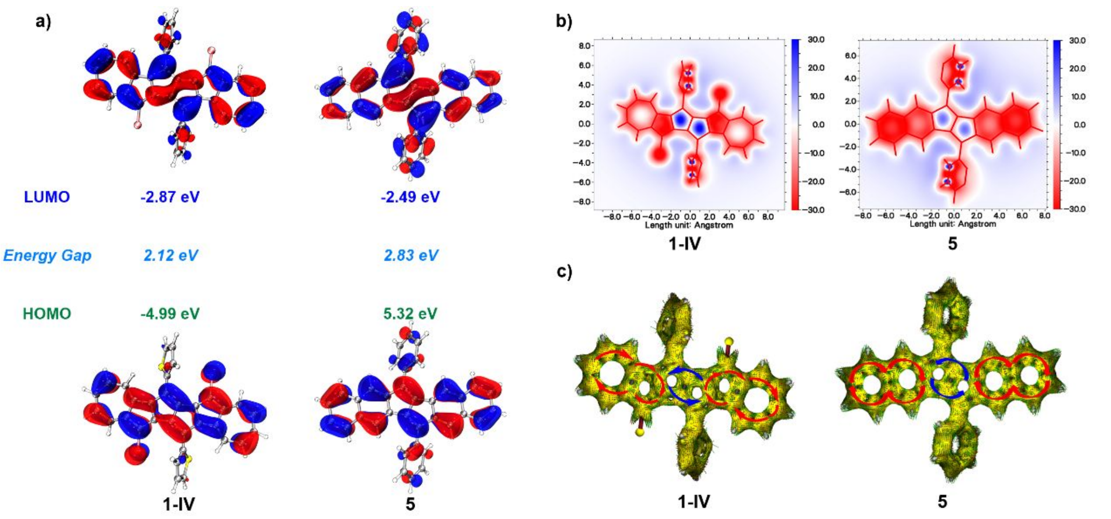

Figure S68. a) Frontier molecular orbital profiles and energy gaps of **1-IV** and **5** (isovalue = 0.02) at the B3LYP/6-311G(d,p) level of theory, alkyl chain was removed to simplify the calculation. b) 2D $NICS_{zz}$ of **1-IV** and **5** at the GIAO-B3LYP/6-311+G(2d,p) level of theory. c) ACID plot of **1-IV** and **5** at the CSGT-B3LYP/6-311G* level of theory. Diamagnetic (clockwise) and paramagnetic (counterclockwise) ring currents under the magnetic field parallel to the z-axis are highlighted by red and blue arrows, respectively.

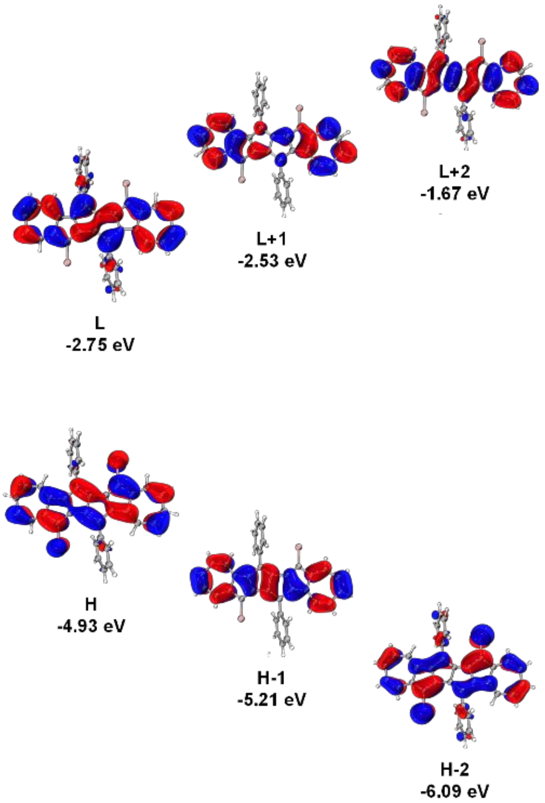

Figure S69. Additional frontier molecular orbital profiles and energy diagram of **1-I** (alkyl chain was removed to simplify the calculation) obtained by B3LYP/6-311G(d,p) level calculation.

Table S4. Vertical excitations (ΔE), oscillation strengths (*f*) and orbital composition of low-lying states of **1-I** (alkyl chain was removed to simplify the calculation) at the Franck-Condon region computed at the PBE0/6-311G(d,p) level (solvent: DCM). (H0, H1, H2, H3 present HOMO, HOMO-1, HOMO-2, HOMO-3 orbitals, respectively. L0, L1, L2, L3, L4 are corresponding to LUMO, LUMO+1, LUMO+2, LUMO+3, LUMO+4 orbitals, respectively.)

State	ΔE_{ex} [eV]	Composition	*f*
S_1	2.5311	$H_1 \rightarrow L_1$ (3 %)<br>$H_0 \rightarrow L_0$ (96 %)	0.6162
S_2	2.6170	$H_1 \rightarrow L_0$ (98 %)	0.0000
S_3	3.2990	$H_2 \rightarrow L_0$ (13 %); $H_0 \rightarrow L_1$ (84 %)	0.0002
S_4	3.4209	$H_2 \rightarrow L_0$ (80 %); $H_1 \rightarrow L_2$ (3 %); $H_0 \rightarrow L_1$ (14 %)	0.0000
S_5	3.6670	$H_3 \rightarrow L_0$ (57 %); $H_1 \rightarrow L_1$ (38 %)	0.5722
S_6	3.8055	$H_4 \rightarrow L_0$ (3 %); $H_3 \rightarrow L_0$ (35 %); $H_1 \rightarrow L_1$ (47 %); $H_0 \rightarrow L_2$ (11 %)	1.0672
S_7	4.0289	$H_4 \rightarrow L_0$ (52 %); $H_3 \rightarrow L_0$ (5 %); $H_2 \rightarrow L_1$ (6 %); $H_1 \rightarrow L_1$ (5 %); $H_0 \rightarrow L_2$ (29 %)	0.4864
S_8	4.0826	$H_4 \rightarrow L_0$ (33 %); $H_2 \rightarrow L_1$ (3 %); $H_1 \rightarrow L_1$ (2 %); $H_0 \rightarrow L_2$ (57 %)	0.2695
S_9	4.1114	$H_7 \rightarrow L_0$ (31 %); $H_5 \rightarrow L_1$ (59 %); $H_1 \rightarrow L_2$ (5 %)	0.0014
S_{10}	4.1492	$H_6 \rightarrow L_0$ (95 %)	0.0148

Table S5. Optimized coordinates of the ground state geometry for **1-I**, alkyl chain was removed to simplify the calculation.

C	-1.96601100	0.95908200	0.01723700
C	-1.75306400	-0.47333000	0.03236600

C	-0.30748900	-0.66397900	0.07091500
C	0.30750800	0.66399400	0.07067900
C	-0.65294700	1.63952400	0.03905000
C	0.65295800	-1.63951600	0.03925300
C	1.96601600	-0.95907800	0.01692800
C	1.75307300	0.47333800	0.03178200
C	-3.23663100	1.47293000	0.01096000
C	-4.36066500	0.59622800	-0.01491000
C	-4.14935600	-0.82108400	-0.02791200
C	-2.81893200	-1.33309000	0.00403700
C	3.23663400	-1.47292800	0.01042600
C	4.36066000	-0.59623100	-0.01595200
C	4.14934800	0.82107800	-0.02922500
C	2.81893300	1.33309100	0.00296600
C	-5.68870600	1.08642100	-0.03051500
C	-6.76347000	0.22582400	-0.06266300
C	-6.55526900	-1.16916900	-0.08010500
C	-5.27557700	-1.67797300	-0.06238900
C	5.68869700	-1.08642800	-0.03180500
C	6.76345200	-0.22583800	-0.06444400
C	6.55524700	1.16915100	-0.08215200
C	5.27556000	1.67795900	-0.06420700
C	0.46702500	-3.10003700	0.02918500

C	-0.46701700	3.10004400	0.02859300
C	0.37588600	3.71999600	0.96399000
C	0.55512000	5.09987000	0.95729500
C	-0.10080200	5.88813100	0.01331600
C	-0.94101500	5.28599600	-0.92119800
C	-1.12833800	3.90698800	-0.91102400
C	-0.37563200	-3.71977200	0.96494800
C	-0.55486700	-5.09964800	0.95862100
C	0.10080600	-5.88812900	0.01465300
C	0.94077400	-5.28621100	-0.92022200
C	1.12809900	-3.90720000	-0.91041800
H	-3.40786000	2.54350000	0.02693000
H	-2.67205300	-2.40685100	0.00720500
H	3.40786800	-2.54349400	0.02659900
H	2.67205400	2.40685300	0.00592500
H	-5.84835900	2.15972600	-0.01740700
H	-7.77376800	0.61900300	-0.07491000
H	-7.40655500	-1.83995100	-0.10678200
H	-5.11369000	-2.75093200	-0.07473400
H	5.84835400	-2.15972900	-0.01849100
H	7.77374700	-0.61902000	-0.07687500
H	7.40652500	1.83992700	-0.10921700
H	5.11366900	2.75091500	-0.07675600

H	0.87336700	3.11217700	1.71076600
H	1.20331800	5.56087500	1.69432500
H	0.04021500	6.96298200	0.00732800
H	-1.45002100	5.89112500	-1.66317200
H	-1.76981200	3.44471700	-1.65191200
H	-0.87291600	-3.11177900	1.71171300
H	-1.20287100	-5.56048100	1.69592800
H	-0.04021200	-6.96298100	0.00895200
H	1.44958400	-5.89151300	-1.66218900
H	1.76937700	-3.44510200	-1.65158300

We have calculated the S1 and S2 state geometries of **1-I** (without alkyl chain) and thereby obtained its emission properties. As shown in Figures S70a and S70b, the oscillator strengths for both S1 and S2 state are 0.000, while the transition energy for each state is 1.189 eV. Notably, for the S2 state, the computed energy of S0→S1 is larger than S0→S2. This result presents an anomaly that violates the fundamental principle of increasing energy with higher excited states. After investigating the excitation energy of each optimization step, we find that there is an intersection between S1 and S2 potential energy surface (PES) (as shown in Figure S70d). This intersection facilitates an internal conversion from S2 to S1. Notably, the S1 state of **1-I** is a dark state, which is consistent with this non-radiative relaxation pathway.

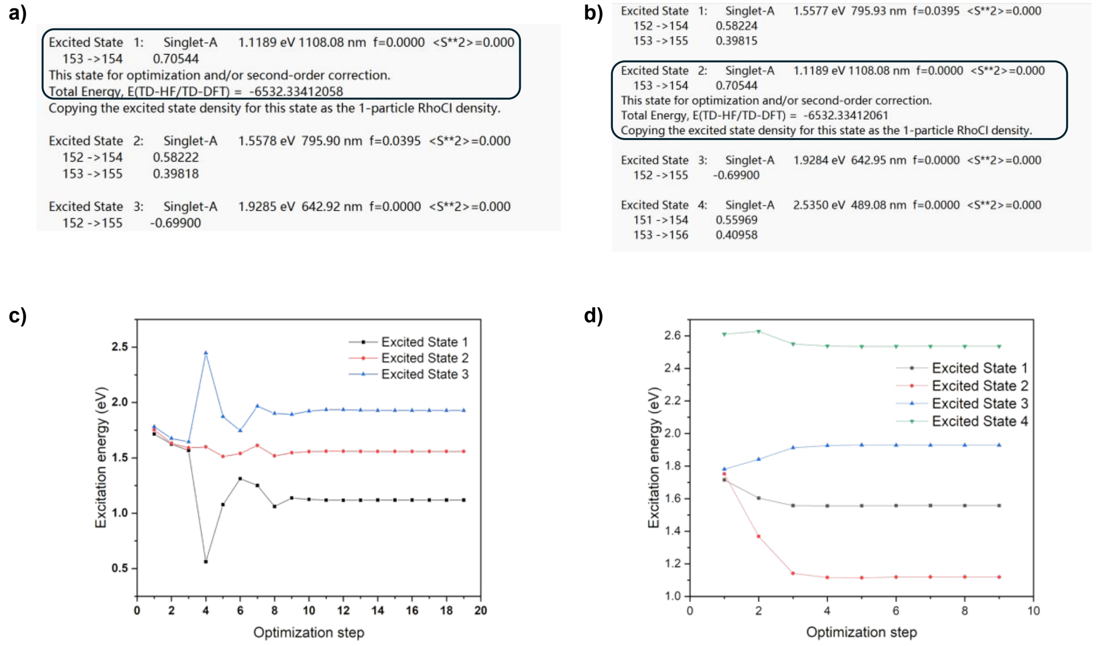

Figure S70. Computational summary of **1-I**. The excited state information of optimized S1 geometry (a) and S2 geometry (b). The excitation energy of S1 state (c) and S2 state (d) during geometry optimization.

7. Others

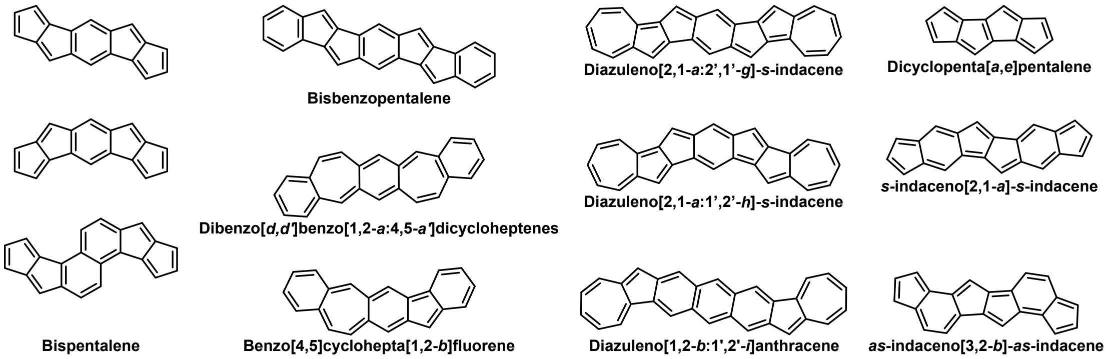

Figure S71. Examples of other π-conjugated systems containing nonhexagons.

8. References

1. S. Budavari, M.J. O'Neil, A. Smith, P.E. Heckelman, The Merck Index, an Encyclopedia of Chemicals, Drugs, and Biologicals-Eleventh Edition, Merck Co., Inc. Rahway, NJ, **1989**.

2. B. Albert, H. Peter, C.Ceciel, S.Jorgen, S. Herman, S. Sepas, H.Willie, M. Christian, L.Dago, V.Josue, B. Cees, B. Dirk, P. Andreea, and M. Stefan *J. Am. Chem. Soc.* **2006**, *128*, 2336-2345.

3. T. Dingemans, D. J. Photinos, E. T. Samulski, A. F. Terzis, C. Wutz *J. Chem. Phys.* **2003** *118,* 7046 - 7061.

4. Gaussian 16, RevisionA.01, M. J. Frisch, G. W. Trucks, H. B. Schlegel, G. E. Scuseria, M. A. Robb, J. R. Cheeseman, G. Scalmani, V. Barone, G. A. Petersson, H. Nakatsuji, X. Li, M. Caricato, A. V. Marenich, J. Bloino, B. G. Janesko, R. Gomperts, B. Mennucci, H. P. Hratchian, J. V. Ortiz, A. F. Izmaylov, J. L. Sonnenberg, D. Williams-Young, F. Ding, F. Lipparini, F. Egidi, J. Goings, B. Peng, A. Petrone, T. Henderson, D. Ranasinghe, V. G. Zakrzewski, J. Gao, N. Rega, G. Zheng, W. Liang, M. Hada, M. Ehara, K. Toyota, R. Fukuda, J. Hasegawa, M. Ishida, T. Nakajima, Y. Honda, O. Kitao, H. Nakai, T. Vreven, K. Throssell, J. A. Montgomery, Jr., J. E. Peralta, F. Ogliaro, M. J. Bearpark, J. J. Heyd, E. N. Brothers, K. N. Kudin, V. N. Staroverov, T. A. Keith, R. Kobayashi, J. Normand, K. Raghavachari, A. P. Rendell, J. C. Burant, S. S. Iyengar, J. Tomasi, M. Cossi, J. M. Millam, M. Klene, C. Adamo, R. Cammi, J. W. Ochterski, R. L. Martin, K. Morokuma, O. Farkas, J. B. Foresman, and D. J. Fox, Gaussian, Inc., Wallingford CT, **2016**.

5. D. Geuenich, K. Hess, F. Köhler, R. Herges, *Chem. Rev.* **2005**, *105*, 3758-3772.

6. (a) Z. Chen, C. S. Wannere, C. Corminboeuf, R. Puchta, P. R. Schleyer, *Chem. Rev.* **2005**, *105*, 3842-3888. (b) P. R. Schleyer, C. Maerker, A. Dransfeld, H. Jiao, N. J. R. E. Hommes, *J. Am. Chem. Soc.* **1996**, *118*, 6317-6318. (c) K. Wolinski, J. F. Hinton, P. Pulay, *J. Am. Chem. Soc.* **1990**, *112*, 8251-8260.

7. Zhao, Y.; Truhlar, D. G. The M06 suite of density functionals for main group thermochemistry, thermochemical kinetics, noncovalent interactions, excited states, and transition elements: two new functionals and systematic testing of four M06-class functionals and 12 other functionals. *Theor. Chem. Acc.* **2008**, *120*, 215-241.

8. Marenich, A. V.; Cramer, C. J.; Truhlar, D. G. Generalized born solvation model SM12. *J. Chem. Theory Comput.* **2013**, *9* (1), 609-620.

9. a) Weigend, F.; Ahlrichs, R. Balanced basis sets of split valence, triple zeta valence and quadruple zeta valence quality for H to Rn: Design and assessment of accuracy. *Physical Chemistry Chemical Physics* **2005**, *7* (18), 3297-3305. b) Weigend, F. Accurate Coulomb-fitting basis sets for H to Rn. *Physical chemistry chemical physics* **2006**, *8* (9), 1057-1065. c) Schäfer, A.; Horn, H.; Ahlrichs, R. Fully optimized

contracted Gaussian basis sets for atoms Li to Kr. *J. Chem. Phys.* **1992**, *97* (4), 2571-2577. d) Schäfer, A.; Huber, C.; Ahlrichs, R. Fully optimized contracted Gaussian basis sets of triple zeta valence quality for atoms Li to Kr. *J. Chem. Phys.* **1994**, *100* (8), 5829-5835.

10. Li, X.; Frisch, M. J. Energy-represented direct inversion in the iterative subspace within a hybrid geometry optimization method. *J. Chem. Theory Comput.* **2006**, *2* (3), 835-839.

11. Fukui, K. The path of chemical reactions-the IRC approach. *Acc. Chem. Res.* **1981**, *14* (12), 363-368.
